# ﻿Scratching the tip of the iceberg: integrative taxonomy reveals 30 new species records of Microgastrinae (Braconidae) parasitoid wasps for Germany, including new Holarctic distributions

**DOI:** 10.3897/zookeys.1188.112516

**Published:** 2024-01-11

**Authors:** Amelie Höcherl, Mark R. Shaw, Caroline Boudreault, Dominik Rabl, Gerhard Haszprunar, Michael J. Raupach, Stefan Schmidt, Viktor Baranov, José Fernández-Triana

**Affiliations:** 1 SNSB-Zoologische Staatssammlung München, Münchhausenstr. 21, 81247 München, Germany SNSB-Zoologische Staatssammlung München München Germany; 2 National Museums of Scotland, Chambers Street, Edinburgh EH1 1JF, UK National Museums of Scotland Edinburgh United Kingdom; 3 Canadian National Collection of Insects, Arachnids and Nematodes, 960 Carling Ave., Ottawa, K1A0C6, Canada Canadian National Collection of Insects, Arachnids and Nematodes Ottawa Canada; 4 Field Station Fabrikschleichach, Department of Animal Ecology and Tropical Biology, Biocenter, University of Würzburg, Glashüttenstr. 5, Würzburg, 96181 Rauhenebrach, Germany University of Würzburg Würzburg Germany; 5 Department Biology II, Ludwig-Maximilians-Universität München (LMU), Großhaderner Str. 2, Martinsried, 82152 Planegg, Germany Ludwig-Maximilians-Universität München Planegg Germany; 6 Estación Biológica de Doñana-CSIC/Doñana Biological Station-CSIC, Seville, Spain Estación Biológica de Doñana-CSIC Seville Spain

**Keywords:** Dark taxa, DNA barcoding, faunistics, host-parasitoid associations, morphology, parasitoid biology

## Abstract

Substantial parts of the European and German insect fauna still remain largely unexplored, the so-called “dark taxa”. In particular, midges (Diptera) and parasitoid wasps (Hymenoptera) are abundant and species-rich throughout Europe, yet are often neglected in biodiversity research. One such dark taxon is Microgastrinae wasps (Hymenoptera: Braconidae), a group of parasitoids of lepidopteran caterpillars with 252 species reported in Germany so far. As part of the German Barcode of Life Project GBOL III: Dark Taxa, reverse DNA barcoding and integrative taxonomic approaches were used to shed some light on the German Fauna of Microgastrinae wasps. In our workflow, DNA barcoding was used for molecular clustering of our specimens in a first step, morphological examination of the voucher specimens in a second step, and host data compared in a third step. Here, 30 species are reported for the first time in Germany, adding more than 10% to the known German fauna. Information for four species is provided in a new Holarctic context, reporting them for the Nearctic or, respectively, Palaearctic region, and 26 additional country records are added from sequenced material available in the collections accessible to us. Molecular clusters that show signs of discrepancies are discussed. Results show that we are just scratching the tip of the iceberg of the unexplored Microgastrinae diversity in Germany.

## ﻿Introduction

With approximately 105,000 insect species documented (Leandro et al. 2017), the Central European fauna is one of the most comprehensively studied in the world (Hausmann et al. 2020; Ronquist et al. 2020; Wagner 2020). Therefore, many studies on insect decline focus their research on this region (Thomas et al. 2004; Hallmann et al. 2017; Seibold et al. 2019; Pilotto et al. 2020). In Germany, in particular, where more than 33,305 insect species have been recorded (Völkl and Blick 2004), there is an exceptionally long history of insect collection, monitoring, and taxonomy (Habel et al. 2016; Hallmann et al. 2017; Jähnig et al. 2021).

However, substantial parts of the German insect fauna remain largely unexplored: the so-called “dark taxa” (Hausmann et al. 2020; Chimeno et al. 2022). This term was first used for DNA sequences with no links to previous information, such as species names, which started to accumulate in public nucleotide databases like NCBI (Page 2016). More recently, “dark taxa” has now been applied to less emblematic, hyperdiverse, and neglected taxa that are especially common in insects (Hausmann et al. 2020; Chimeno et al. 2022; Hartop et al. 2022), nematodes, or chelicerates (e.g., mites). The concept includes various groups of Diptera (flies) and Hymenoptera (bees, wasps, ants, and sawflies) – here, in particular, parasitoid wasps (Hausmann et al. 2020). Although representatives of these taxa are often very small in size, they can make up more than half of the specimens of a Malaise trap sample (Brown 2005; Karlsson et al. 2020; Wührl et al. 2022). Several factors contribute to the neglect of a taxon: First, many species descriptions in the historical literature are short, only providing inadequate or low-quality figures, and types may be lost or damaged. Second, the identification of many species relies on a combination of very subtle morphological characteristics, which are often difficult to interpret and assess, especially in closely related species or those forming part of morphologically cryptic species complexes. Third, only a comparatively small number of taxonomists focus on these groups.

As part of the German Barcode of Life Initiative, GBOL III: Dark Taxa project, we aim to change this situation by applying a reverse DNA barcoding and integrative taxonomic approach. In contrast to traditional DNA barcoding workflows, we first performed molecular analyses, tested these results via morphological comparison in a second step, and compared available host data in a third step. Our molecular work relies on DNA barcoding (Hebert et al. 2003a), a technique that uses short, standardised genetic markers such as the cytochrome *c* oxidase subunit I (COI) gene of the mitochondrial genome for molecular species identification (Hebert et al. 2003b). DNA barcoding has proven to be a powerful tool for valid species identification (Hebert et al. 2003a), including for many German taxa (e.g., Hausmann et al. 2011a; Raupach et al. 2014; Hendrich et al. 2015; Morinière et al. 2017). However, there are some constraints to DNA barcoding, such as introgression of mitochondrial DNA into the nuclear genome (numts) (e.g., Hebert et al. 2023), incomplete lineage sorting, recent or ongoing hybridisation events (e.g., Mutanen et al. 2016), and effects of *Wolbachia* infections (e.g., Smith et al. 2012). We aim to mitigate these by using an integrative approach that includes morphology and host information in addition to molecular evidence to support our species concepts. Especially in this dark taxa context, a reverse DNA barcoding approach has major advantages: (1) Species identification is facilitated, though still limited by significant gaps in reference libraries for dark taxa; (2) Clustering large numbers of specimens and thereby increasing the efficiency of taxonomic workflows (Brown et al. 2018; Fernandez-Triana 2022; Hartop et al. 2022); and (3) Enabling species discovery by uncovering morphologically cryptic diversity through DNA barcoding (Fernandez-Triana et al. 2014c; Smith et al. 2015; Lazarević et al. 2023).

In the last two decades, DNA barcoding and integrative taxonomy have revolutionised the study of Microgastrinae parasitoid wasps (e.g., Smith et al. 2013; Fernandez-Triana et al. 2014c; Fagan-Jeffries et al. 2018). Microgastrinae (Hymenoptera: Braconidae) is a common and highly diverse group of parasitoids of lepidopteran caterpillars, with more than 3,200 species described worldwide, but a projected diversity estimated up to 40,000–50,000 species (Fernandez-Triana et al. 2020). A total of 252 species of Microgastrinae has been recorded in Germany until now (Belokobylskij et al. 2003; Fernandez-Triana et al. 2020; Papp 1981b; Shaw 2020, 2022). Based on known host/parasitoid ratios and a host diversity of currently 3,688 established Lepidoptera species recorded from Germany (Rennwald et al. 2023), it is very likely that the officially recorded Microgastrinae fauna in the country is substantially underrepresenting the actual species-richness of this group in Germany. As a result of our approach, we report 30 species in Germany for the first time, adding more than 10% to the known German fauna. In ten cases, we link sequences to a species name for the first time and place four species in a new Holarctic context, reporting them for the Nearctic or Palaearctic regions for the first time.

## ﻿Materials and methods

**Figure 1.**
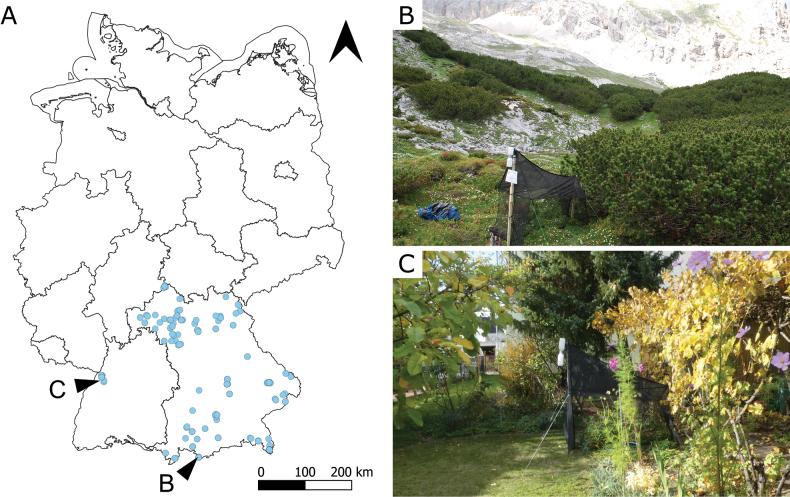
**A** Map showing our sampling locations in Germany, mostly in Bavaria (GeoBasis-DE / BKG 2022). The sampled habitats include a large variety from **B** the highest mountain in Germany, Zugspitze (photograph: J. Voith) to **C** an urban garden (photograph: D. Dozckal).

Specimens were collected in southern Germany (Fig. 1A) using mostly Malaise traps, but also canopy fogging. We have sampled a variety of localities and habitats from urban gardens, nature reserves and former military shooting ranges to the highest mountain in Germany, Zugspitze (Fig. 1B, C). Our dataset contains a total of 5455 specimens from Germany, of which 5364 yielded COI sequences. We also studied reared specimens from the National Museums of Scotland (**NMS**), Edinburgh, the Zoologische Staatssammlung München (**ZSM**), as well as specimens from the Canadian National Collection of Insects and Arachnids (**CNC**). We downloaded additional sequences and distribution data from the public and private data available to us in the Barcode of Life Data System (BOLD) (Ratnasingham and Hebert 2007). In accordance with our reverse DNA barcoding approach, we first used a molecular workflow for clustering, then a morphological workflow and additionally looked at host data to establish our integrative species concepts. This order of different approaches represents our workflow and does not indicate that we favoured any single one of these methods. We used molecular information as well as morphology for every single species and additionally considered biological information if available. Detailed information about these integrative species concepts is provided in the notes section of the species.

### ﻿Molecular workflow

We manually size-fractioned Malaise trap bulk samples by sieving and then sorted into first-glance morphotypes, of which subsamples were chosen for sequencing. We used legs as tissue samples, depending on size of the specimen one to three legs. COI-sequencing was done at the CCDB (Canadian Centre for DNA Barcoding) using their at-the-time standard sequencing protocols and primers, which can be reviewed for each sequence in the BOLD database (www.boldsystems.org). Sequences were analysed and clustered using the BOLD workbench and database (Ratnasingham and Hebert 2007). We used the Barcode Index Numbers (BINs) approach (Ratnasingham and Hebert 2013) to define molecular operational taxonomic units (MOTUs) for establishing our molecular species hypotheses. We used BIN distances from the BOLD database to identify possible BIN discrepancies and in some cases calculated distance matrices in BOLD or MEGA for intra- or interspecific distances (Kimura 2-parameter pairwise distances). For those BINs where we found possible BIN-discrepancies, we performed TCS haplotype analysis using PopART (Snell et al. 2002; Leigh and Bryant 2015) and performed clustering using ASAP (Puillandre et al. 2021). The sequences used for our analyses were selected based on sequence length and number of ambiguous characters. For haplotype network analysis, the sequence length is indicated in the description of the figures depicting the haplotype networks (Figs 14, 34, 36), sequence alignments and traits are attached in the Suppl. materials, and the ASAP partitions can be reviewed in Suppl. materials 14 and 15. BINs and BIN assignments are not static and may change as new data is added to BOLD (Ratnasingham and Hebert 2013). Our BIN assignments refer to the latest download of our dataset DS-MCGNRECG on 9 Aug 2023. All COI sequences are attached in Suppl. material 2.

### ﻿Morphological workflow

We chemically dried our voucher specimens from Germany using a modified Hexamethyldisilazane (HMDS) protocol (Heraty and Hawks 1998; Rumph and Turner 1998) and glued specimens to points using shellac gel or white glue. Historical specimens may have been processed differently. For morphological identification we used various identification keys by Gilbert E. J. Nixon (1965, 1968, 1970, 1972, 1973, 1974, 1976), Jenő Papp (1976a, b, 1978, 1979, 1980, 1981a, 1982, 1983, 1984a, b, 1986a, 1987, 1988, 1990), and Vladimir I. Tobias and Anatoly G. Kotenko (Tobias 1986). Material was also compared to original descriptions and to type material or authoritatively identified material stored in the collections of the CNC, ZSM, or NMS. If photographs of types or authenticated material were available due to previous work done in other institutions, we compared our specimens to these. We provide information about literature and material used for our identifications in the notes for each species. Terminology and measurements used here are explained in detail in Fernandez-Triana et al. (2014c).

### ﻿Biology (host information)

We checked original descriptions for collecting any host information related to type material. The supposed host data compiled by literature abstraction, such as in the database Taxapad 2016 (Yu et al. 2016), was generally ignored due to its inherently low reliability caused by uncritically citing host associations from literature (Shaw 1994). However, we used this database to selectively track literature on host information, checked the original literature in every single case, and interpreted this information critically. Most importantly, we checked any reared and barcoded material available to us between the collections of the CNC, NMS, and ZSM. We also had access to metabarcoding data from barcoding whole caterpillars as part of the GBOL project. Host information is discussed in the notes section for each species. Synonyms and current combinations for Lepidoptera hosts were checked in Lepiforum’s latest checklist for European Lepidoptera (Rennwald et al. 2023).

### ﻿Additional information, abbreviations, and terminology

New information is marked by an asterisk (*). All original descriptions of the species we report here are cited in the References section. Specimens were photographed using a Keyence VHX-6000 digital microscope and panorama stacks were computed using the built-in software of the microscope. Subsequent processing and construction of image plates and figures was done using Photoshop and Inkscape. Maps were done using QGIS and Inkscape. Voucher codes that are referred to in the notes and material examined sections refer to the “SampleID” in BOLD, more information about these specimens can be retrieved from the supplementary material or from BOLD. We have, however, added MS, MRS, or MRS_JFT voucher codes for barcoded specimens housed in NMS to facilitate retrieval for further examination. Distribution data of specimens is based on the annotated world checklist, as well as abbreviations and limits for the biogeographical regions used by Fernandez-Triana et al. (2020). Abbreviations for biogeographical regions are as follows: **NEO** Neotropical, **NEA** Nearctic, **PAL** Palaearctic, **OTL** Oriental, **AFR** Afrotropical, and **AUS** Australasian and Oceanian (combined following O’Hara et al. 2009). The Holarctic includes the Nearctic and Palaearctic regions. For subsequently described or recorded species, the distribution range is based on the data provided in the respective publication (Shaw 2020, 2022, 2023). Syntax for the Material examined section is as follows: “**Country**: Province: Exact Location, coordinates in decimal degrees, elevation, collection method/host, collection date, collector(s), voucher code”. If several samples were collected at the same collecting event, several voucher codes are listed consecutively and separated by semicolons. Extrapolated information is bracketed. Different collecting events from the same location are also grouped and are separated by a semicolon. Additional information, e.g., about the sex of a specimen or the storing institution can be reviewed in the Suppl. material 1 and, if any changes happen in the future, will be updated in the BOLD database. Malaise trap specimens were collected during a period of 1–4 weeks (depending on the season: in spring and autumn collecting bottles were left for up to four weeks, and in summer (especially May, June, July, August, and even September) usually not more than two weeks. The date indicated for the collection event represents the day the bottle was collected. For a number of species collected in the former Soviet Union we were able to translate the labels and found that they were collected in countries (Armenia, Moldova, and Ukraine) that were not then formally recorded for these species, but labelled from Russia (which we mitigate here).

## ﻿Results

### ﻿Species recorded for Germany and other regions for the first time

#### ﻿ Apanteles galleriae

Taxon classificationAnimaliaHymenopteraBraconidae

Wilkinson, 1932

3072DA47-AEF5-5C67-88F0-6BA0CAA5F995

##### Material examined.

**Germany**: Baden-Württemberg, Malsch, Hansjakobstr. 7, Urban Garden, 48.884, 8.32, 120 m, Malaise trap, 27.ix.2020, leg. D. Doczkal, ZSM-HYM-33154-A11; ZSM-HYM-33154-A12; Bavaria: Passau, Heining, 48.583, 13.391, 432 m, Malaise trap, 13.vii.2019, leg. J. Müller, ZSM-HYM-42384-E09; **Malta**: Zejtun, ex. *Achroiagrisella*, 15.viii.2013, leg. D. Mifsud, MRS_JFT0380; **United Kingdom**: England: Battle, Sussex, 50.917489, 0.483602, ex. beehive with *Galleriamellonella*, xii.2011, leg. J. Feltwell, CNCHYM45392.

##### Geographical distribution.

AFR, AUS, NEA, NEO, OTL, PAL.

AFR- Mauritius, Réunion; AUS- Hawaiian Islands, New Zealand; NEA- Canada (BC), United States (GA, NC, OH, SC); NEO- Argentina, Brazil (SP); OTL- China (FJ, GD, GX, GZ, HI, HN, JX, TW, ZJ), India, Pakistan; PAL- Armenia, Bulgaria, France, Greece, Germany*, Hungary, Iran, Italy, Japan, Malta, Romania, Russia (PRI), Spain, Turkey, United Kingdom.

##### Molecular data.

BIN: BOLD:AAG1400.

##### Host information.

Pyralidae: type reared from *Galleriamellonella* (Linnaeus, 1758) (Wilkinson 1932); also *Achroiagrisella* (Fabricius, 1794), *Achroiainnonata* (Walker, 1864), *Vitulaedmandsii* (Packard, 1865).

##### Notes.

**Figure 2.**
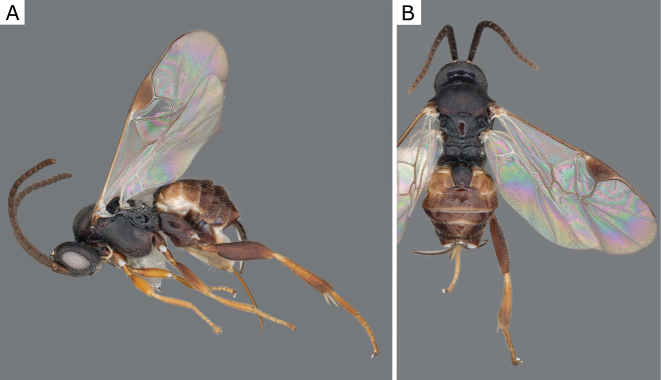
*Apantelesgalleriae* Wilkinson, 1932, female (ZSM-HYM-42384-E09) **A** lateral and **B** dorsal views. Length of the specimen: 2.6 mm.

Specimens in BIN BOLD:AAG1400 morphologically identified as *Apantelesgalleriae* were reared from both *Galleriamellonella* (CNCHYM45392=MRS_JFT 0107 ex. beehive with that species) and *Achroiagrisella* (MRS_JFT0380). Additional host species recorded for non-type specimens are based on literature (e.g., Shimamori 1987; Watanabe 1987; Okada 1988; Whitfield and Cameron 1993); they are considered to be accurate because of the detailed evidence provided in those papers (rearing details, photos, and images), the expertise of the researchers that identified the parasitoids, and the fact that those host species are all wax moths, related to *G.mellonella*. Additional provinces for China are from Liu et al. (2020). This species is illustrated in Fig. 2.

#### ﻿ Apanteles kubensis

Taxon classificationAnimaliaHymenopteraBraconidae

Abdinbekova, 1969

1BF393FA-455D-5DDD-82DB-541F5B8A6D6E

##### Material examined.

**Germany**: Baden-Württemberg: Malsch, Hansjakobstr. 7, Urban Garden, 48.884, 8.32, 120 m, Malaise trap, 16.viii.2020, leg. D. Doczkal, ZSM-HYM-33153-F03; Bavaria: Fabrikschleichach, close to Weilersbachtal, 49.917, 10.525, 408 m, Malaise trap, 12.vii.2019, leg. J. Müller, ZSM-HYM-42376-G08; **Korea**: Daejon-si, Wadong; Chungnam, 36.4006, 127.444, 6.x.2006, leg. P. Tripotin, CNCH2502; CNCH2522; CNCH2526; **Ukraine**: [translated and transcribed from Russian] Crimea, Angarskiy pass, forest, glades, 11.vii.1979, leg. A. Kotenko, CNCHYM 00136.

##### Geographical distribution.

PAL.

PAL- Azerbaijan, Germany*, Hungary, Iran, Korea, Moldova, Mongolia, Russia (NC, S), Turkey, Ukraine*.

##### Molecular data.

BIN: BOLD:AAH1340.

##### Host information.

Host of type unknown (Abdinbekova 1969); also Tortricidae: *Adoxophyesorana* (Fischer von Röslerstamm, 1834).

##### Notes.

**Figure 3.**
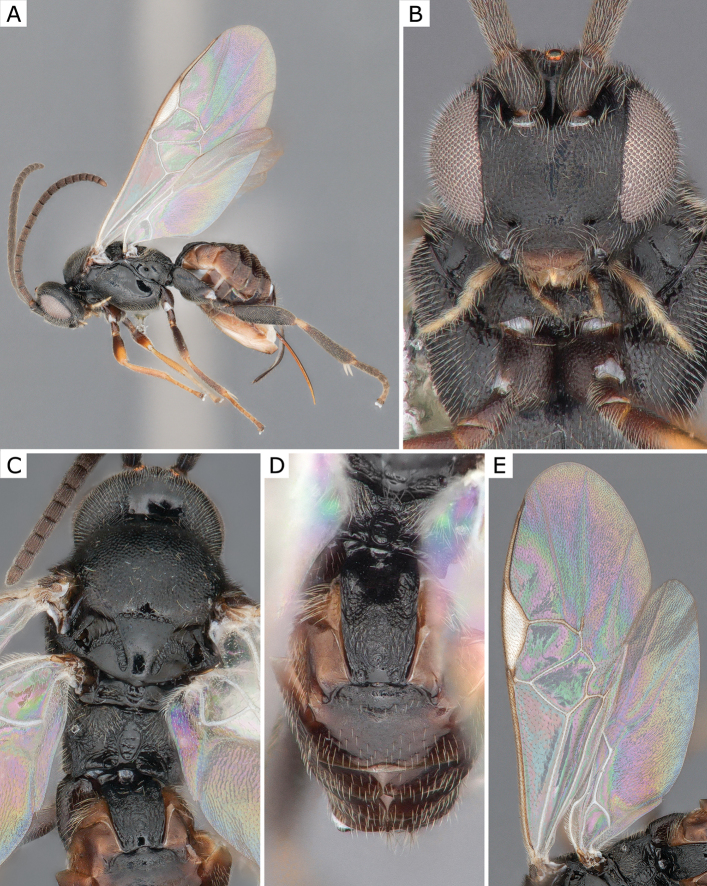
*Apanteleskubensis* Abdinbekova, 1969, female (ZSM-HYM-42376-G08) **A** lateral view **B** head frontal view **C** mesosoma **D** metasoma **E** wing. Length of the specimen: 3 mm.

**Figure 4.**
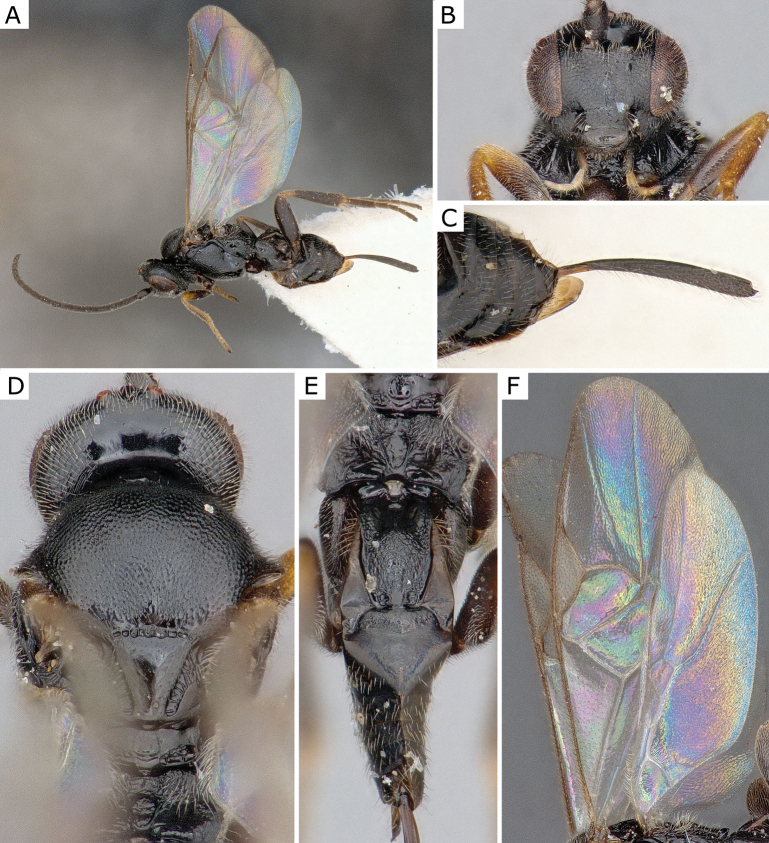
*Apanteleskubensis* Abdinbekova, 1969, female (CNCHYM 00136) **A** lateral view **B** head frontal view **C** hypopygium lateral view **D** mesosoma **E** metasoma **F** wing.

The German specimens were compared with a specimen from Ukraine (CNCHYM 00136=CNC280641) which had been identified by Kotenko in 1981 and donated to the CNC. We also checked the information in Tobias (1986) (the key to “Apanteles” sensu lato species in Tobias (1986) was written in collaboration with Kotenko) and also the key in Papp (1980). Additionally, we studied CNC specimens from South Korea (CNCH2502, CNCH2522, CNCH2526) with DNA barcodes that match the sequences from German specimens and the Ukrainian specimen sent to the CNC by Kotenko. The species was recorded from Korea by Ku et al. (2001), and one of the coauthors of that paper, the Braconidae expert Sergey Belokobylskij, works in the institution storing the type of *A.kubensis*. In BOLD there is also an additional specimen from the Primorskiy Kray, Russia (BIOUG27804-B05) that perfectly matches the German, Korean, and Ukrainian sequences; it most likely represents an additional record of the species for the Russian Far East, but we do not report it here because we could not examine the specimen. The only host reported for *A.kubensis* in the literature (Ku et al. 2001) is from Korea. The distribution in Iran was reported by Samin et al. (2020). This species is illustrated in Figs 3, 4.

#### ﻿ Choeras ciscaucasicus

Taxon classificationAnimaliaHymenopteraBraconidae

(Tobias, 1971)

122C3C85-D4AF-5FE4-8B00-13B5EFAB7F8B

##### Material examined.

**Czech Republic**: South Moravia, Obora Soutok, Lanzhot, 48.69, 16.945, 165 m, ex. *Sterrhopterixfusca*, 09.v.2014, leg. P. Drozd, BC-ZSM-HYM-23872-B02; **Germany**: Bavaria: Aub, 49.542, 10.053, 316 m, canopy fogging, 10.vii.2020, leg. B. Leroy, ZSM-HYM-42392-B10; Bad Windsheim, 49.482, 10.468, 382 m, canopy fogging, 3.vii.2019, leg. B. Leroy, ZSM-HYM-33158-H02; Iphofen, 49.646, 10.315, 355 m, canopy fogging, 2.vii.2019, leg. B. Leroy, ZSM-HYM-33158-H10; Theres, 49.997, 10.412, 275 m, canopy fogging, 2.vii.2019, leg. B. Leroy, ZSM-HYM-33159-A07.

##### Geographical distribution.

PAL.

PAL- Czech Republic*, Germany*, Lithuania, Russia (AD, PRI).

##### Molecular data.

BIN: BOLD:ACU3996.

##### Host information.

Host of type unknown; also Psychidae*: Sterrhopterixfusca* (Haworth, 1809).

##### Notes.

**Figure 5.**
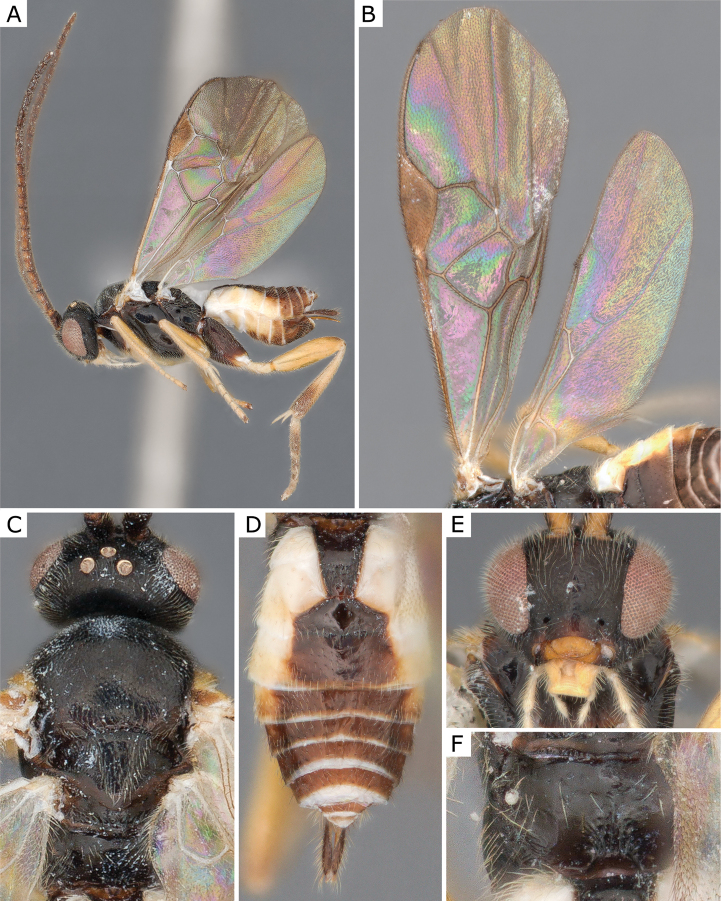
*Choerasciscaucasicus* (Tobias, 1971), female (ZSM-HYM-42392-B10) **A** lateral view **B** wing **C** mesosoma **D** metasoma **E** head frontal view **F** propodeum. Length of the specimen: 2.85 mm.

**Figure 6.**
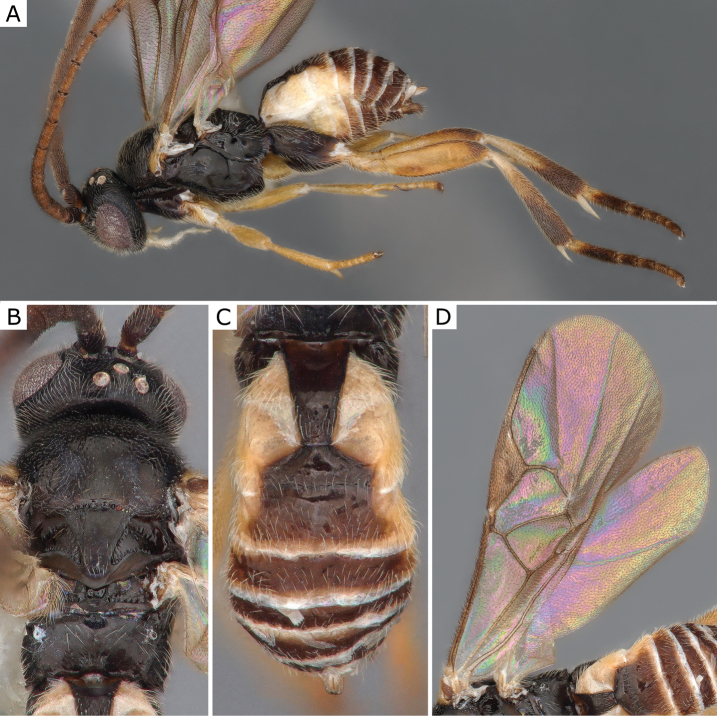
*Choerasciscaucasicus* (Tobias, 1971), male (ZSM-HYM-33159-A07) **A** lateral view **B** mesosoma **C** metasoma **D** wing. Length of the specimen: 2.65 mm.

This species is morphologically very distinct from all other Palaearctic species of *Choeras* and can be identified by the combination of the following characters: T1 strongly narrowing; ovipositor sheaths short, ~ ½ length of metatibia; propodeum smooth and shiny; T1 and T2 smooth, with only slight wrinkles on the posterior half of T1. Our German specimens keyed out as *Choerasciscaucasicus* in all keys that we used (Papp 1983; Tobias 1986; van Achterberg 2002; Kotenko 2007). One Czech Republic specimen stored at the ZSM (BC-ZSM-HYM-23872-B02) was reared from *Sterrhopterixfusca* (Psychidae) and represents the first host associated with *Choerasciscaucasicus*. This species is illustrated in Figs 5, 6.

#### ﻿ Choeras gnarus

Taxon classificationAnimaliaHymenopteraBraconidae

(Tobias & Kotenko, 1984)

D87D24A6-9FE3-5AFD-B3EF-11CA5877060E

##### Material examined.

**Germany**: Baden-Württemberg: Gaggenau, Michelbach, 48.821, 8.388, 340 m, Malaise trap, 9.vii.2011, leg. D. Doczkal, ZSM-HYM-42398-H04; Gaggenau, Sulzbach, Querbach, 48.797, 8.378, 375 m, Malaise trap, 21.viii.2011, leg. D. Doczkal, ZSM-HYM-42323-A10; Malsch, Hansjakobstr. 7, Urban Garden, 48.884, 8.32, 120 m, Malaise trap, 16.viii.2020, leg. D. Doczkal, ZSM-HYM-33153-F04; 19.vii.2020, leg. D. Doczkal, ZSM-HYM-33154-G09; 2.viii.2020, leg. D. Doczkal, ZSM-HYM-33154-H03; 21.vi.2020, leg. D. Doczkal, ZSM-HYM-33152-G01; 5.vii.2020, leg. D. Doczkal, ZSM-HYM-33154-E12; Malsch, Luderbusch, 48.913, 8.332, 117 m, Malaise trap, 16.viii.2020, leg. D. Doczkal, K. Grabow, ZSM-HYM-42389-C05; 26.vii.2020, leg. D. Doczkal, K. Grabow, ZSM-HYM-42388-G07; 9.viii.2020, leg. D. Doczkal, K. Grabow, ZSM-HYM-42389-B05; Bavaria: Ammergebirge Halblech, Im Laich, gravel bar, 47.606, 10.841, 901 m, Malaise trap, 17.viii.2016, leg. D. Doczkal, J. Voith, ZSM-HYM-33166-F09; 29.vii.2016, leg. D. Doczkal, J. Voith, ZSM-HYM-33166-E07; 904 m, Malaise trap, 17.viii.2016, leg. D. Doczkal, J. Voith, ZSM-HYM-33167-C07; 29.vii.2016, leg. D. Doczkal, J. Voith, ZSM-HYM-33167-A04; ZSM-HYM-33167-A05; ZSM-HYM-33167-A07; ZSM-HYM-33167-A08; Aub, 49.542, 10.053, 316 m, fogging, 10.vii.2020, leg. B. Leroy, ZSM-HYM-42392-D03; Bamberg, Naturwaldreservat Wolfsruhe, Bruderwald, 49.856, 10.899, 282 m, Malaise trap, 13.vii.2019, leg. J. Müller, ZSM-HYM-42382-B05; ZSM-HYM-42382-B06; Chiemgau Alps Ruhpolding, Fischbach, 47.709, 12.657, 720 m, Malaise trap, 16.viii.2016, leg. D. Doczkal, J. Voith, ZSM-HYM-33168-B02; Fabrikschleichach, close to Weilersbachtal, 49.917, 10.525, 408 m, Malaise trap, 12.vii.2019, leg. J. Müller, ZSM-HYM-42376-E07; Haselbach, Wald, 48.642, 11.019, 485 m, Malaise trap, 15.vii.2019, leg. J. Müller, ZSM-HYM-42383-A11; Jöslein, Forst Neustädtlein am Forst, 49.992, 11.482, 411 m, Malaise trap, 13.vii.2019, leg. J. Müller, ZSM-HYM-42385-F03; Lohr a. M., Romberg, 49.986, 9.59, 185 m, Malaise trap, 6.vii.2018, leg. D. Doczkal, ZSM-HYM-42323-F09; Marquartstein, close to Berg Torkopf, 47.767, 12.43, 786 m, Malaise trap, 19.vii.2019, leg. J. Müller, ZSM-HYM-42383-F04; Mauth, Naturpark Bayerischer Wald, 48.89, 13.563, 858 m, Malaise trap, 12.vii.2019, leg. J. Müller, ZSM-HYM-42381-C04; Moos, Isarmündung, 48.786, 12.959, 313 m, Malaise trap, 13.vii.2021, leg. GBOL3, R. Albrecht, ZSM-HYM-42394-F09; Moos, Isarmündung, *Molinia* meadow, 48.779, 12.95, 313 m, Malaise trap, 25.viii.2021, leg. GBOL3, R. Albrecht, ZSM-HYM-42394-E11; München, NSG Allacher Lohe, 48.201, 11.483, 499 m, Malaise trap, 21.vii.2021, leg. GBOL3, R. Albrecht, ZSM-HYM-42326-H02; Berchtesgaden National Park, Königssee, Rinnkendlsteig, 47.551, 12.964, 695 m, Malaise trap, 4.x.2017, leg. D. Doczkal, J. Voith, ZSM-HYM-33162-B10; Berchtesgaden National Park, Wald west of St. Bartholomä, 47.547, 12.965, 620 m, Malaise trap, 28.vi.2017, leg. D. Doczkal, J. Voith, ZSM-HYM-33156-G05; Oberndorf, close to Krebsbach, 49.868, 9.516, 342 m, Malaise trap, 13.vii.2019, leg. J. Müller, ZSM-HYM-42382-H01; Willersdorf, Untere Mark, 49.733, 10.985, 292 m, Malaise trap, 12.vii.2019, leg. J. Müller, ZSM-HYM-42379-A06; München, Obermenzing, Premises of Zoologische Staatssammlung, 48.1648, 11.4849, 519 m, Malaise trap, 31.vii.2017, leg. Axel Hausman, BIOUG42697-G11; **Moldova**: [translated and transcribed from Russian] Rajon Anenii Noi, Hîrbovăț, 4.vi.1986, CNCHYM 00280; **Sweden**: Dalarna: Säterdalen, Näsåkerspussen; Säters kommun, 60.366667, 15.716667, Malaise trap, 8–21.vii.2003, leg. SMTP, CNC472136; [no collection information associated] WAM 0076; Sm, Nybro kommun, Alsterbro/Alsteran., 63.122200, 15.069970, Malaise trap, 20–25.viii.2005, leg. SMTP, CNC1967347.

##### Geographical distribution.

PAL.

PAL- Belarus, Germany*, Moldova*, Russia (NC, C), Sweden*, Ukraine.

##### Molecular data.

BIN: BOLD:AAU6216.

##### Host information.

Unknown.

##### Notes.

**Figure 7.**
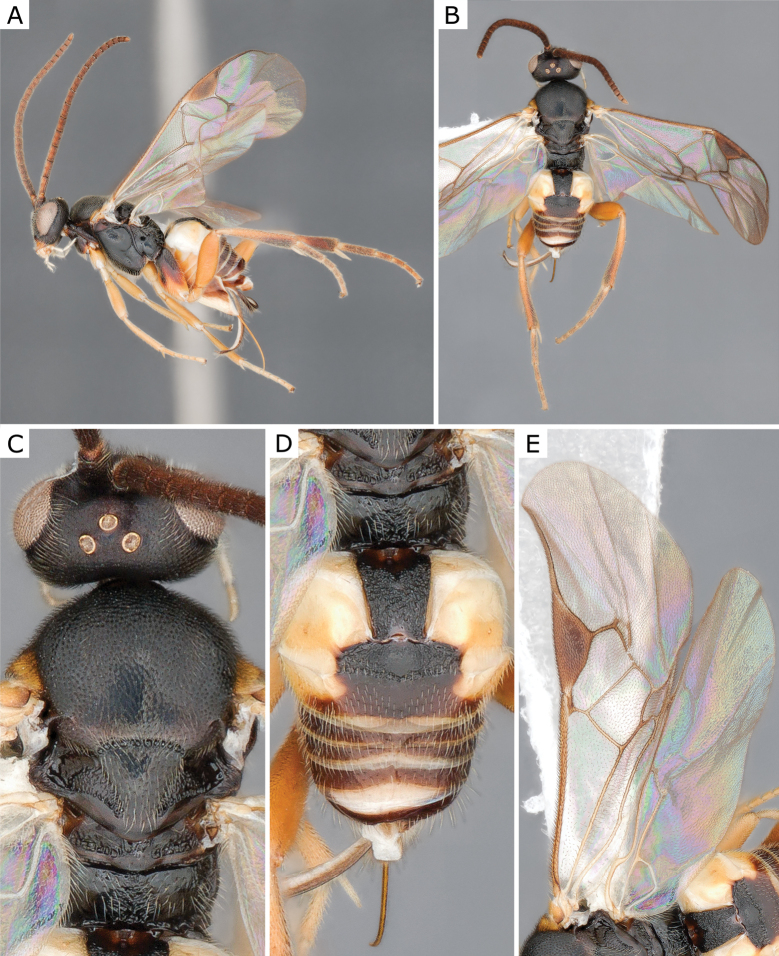
*Choerasgnarus* (Tobias & Kotenko, 1984), female (ZSM-HYM-33167-A07) **A** lateral view **B** dorsal view **C** mesosoma **D** metasoma **E** wing. Length of the specimen: 3.35 mm.

**Figure 8.**
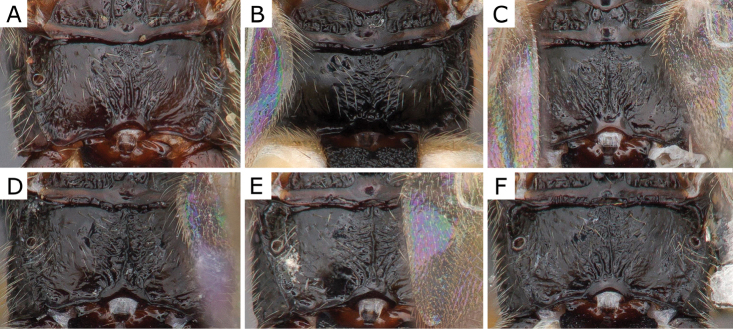
*Choerasgnarus* (Tobias & Kotenko, 1984) female propodeum **A** CNCHYM 00280 (Moldova) **B**ZSM-HYM-33167-A07 (Germany) **C**ZSM-HYM-42379-A06 (Germany) **D** CNC472136 (Sweden) **E**ZSM-HYM-33162-B10 (Germany) **F**ZSM-HYM-33167-A04 (Germany).

**Figure 9.**
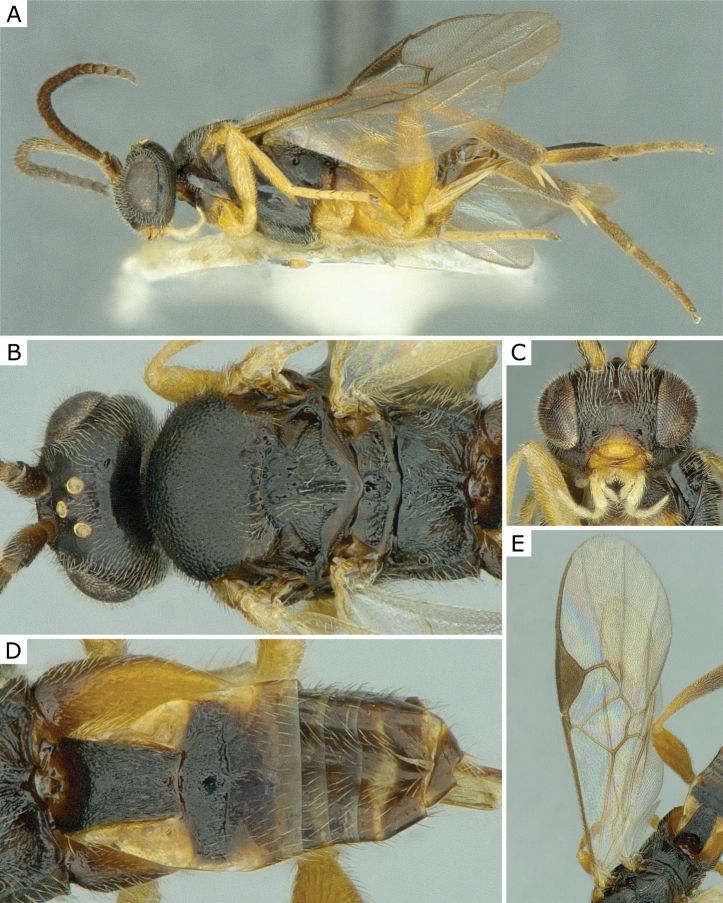
*Choerasgnarus* (Tobias & Kotenko, 1984), female (CNCHYM 00280) identified by A. Kotenko **A** lateral view **B** mesosoma **C** head frontal view **D** metasoma **E** wing. Length of the specimen: 2.95 mm.

The German specimens were compared with a specimen from Moldova (CNCHYM 00280) which had been identified by Kotenko in 1986 and donated to the CNC. We also ran our specimens through the keys of Tobias (1986), van Achterberg (2002), and Kotenko (2007) where they match *Choerasgnarus* also in accordance with Kotenko’s specimen at the CNC (CNCHYM 00280). In Abdoli et al. (2019), some of our specimens match *Choerasformosus* Abdoli & Fernandez-Triana, 2019 from Iran, based on the presence of a well-defined median carina on the propodeum. However, this character is variable in our specimens ranging from irregular rugosities to an incomplete to clearly defined median carina (see Fig. 8). In addition to morphology, our barcodes match a barcoded specimen from Moldova (CNCHYM 00280) by 100%, authoritatively identified by A. Kotenko, who is an author of the species. The presence/absence of a median carina as a character to identify species of *Choeras* may have to be reassessed in the future. This species is illustrated in Figs 7–9.

#### ﻿ Cotesia coryphe

Taxon classificationAnimaliaHymenopteraBraconidae

(Nixon, 1974)

0C4013B4-D414-5368-89A3-9BD9B0CDC43A

##### Material examined.

**Austria**: Hinterreit, Gemeinde Großgmain, 47.75, 12.946, 493 m, ex. *Hemarisfuciformis*, 22.vi.2021, leg. W. Langer, ZSM-HYM-ZLAB01-E11; **Germany**: Baden-Württemberg: Malsch, Hansjakobstr. 7, Urban Garden, 48.884, 8.32, 120 m, Malaise trap, 2.viii.2020, leg. D. Doczkal, ZSM-HYM-33154-H02; Bavaria: Arnstein, Rieden, 49.938, 10.051, 260 m, Malaise trap, 16.vii.2019, leg. J. Müller, ZSM-HYM-42385-H08; Bad Königshofen, 50.292, 10.484, 274 m, Malaise trap, 16.vii.2019, leg. J. Müller, ZSM-HYM-42384-E11; Forchheim, Untere Mark close to Willersdorf, 49.739, 10.969, 261 m, Malaise trap, 12.vii.2019, leg. J. Müller, ZSM-HYM-42377-E08; Isarmündung, Magerrasen, swampy, 48.78, 12.966, 313 m, sweeping, 30.vi.2021, leg. A. Höcherl, ZSM-HYM-ZLAB01-C05; Lkr. Kelheim Abensberg-Sandharlanden, NSG Sandharlandener Heide, 48.845, 11.801, 376 m, Malaise trap, 3.viii.2017, leg. D. Doczkal, J. Voith, ZSM-HYM-33157-E11; ZSM-HYM-33157-G09; ZSM-HYM-33157-G10; ZSM-HYM-33157-G11; ZSM-HYM-33157-G12; 8.ix.2017, leg. D. Doczkal, J. Voith, ZSM-HYM-33157-H07; Moos, Isarmündung, Magerrasen, swampy, 48.78, 12.966, 313 m, Malaise trap, 25.viii.2021, leg. GBOL3, R. Albrecht, ZSM-HYM-42396-F08; München, NSG Allacher Lohe, 48.199, 11.475, 502 m, Malaise trap, 21.vii.2021, leg. GBOL3, R. Albrecht, ZSM-HYM-42326-D06; 23.vi.2021, leg. GBOL3, R. Albrecht, ZSM-HYM-42326-B06; Berchtesgaden National Park, Königssee, Rinnkendlsteig, 47.553, 12.964, 775 m, Malaise trap, 30.vii.2017, leg. D. Doczkal, J. Voith, ZSM-HYM-33161-B03; Oberndorf, close to Krebsbach, 49.868, 9.516, 342 m, Malaise trap, 13.vii.2019, leg. J. Müller, ZSM-HYM-42382-G12; Oberstdorf, Oytal, rubble cone east of Gleitweg, 47.389, 10.348, 1200 m, 16.vi.2014, leg. D. Doczkal, S. Schmidt, J. Voith, ZSM-HYM-33420-A03; Sielstetten, östlich Grafendorfer Forst, 48.578, 11.863, 520 m, Malaise trap, 16.vii.2019, leg. J. Müller, ZSM-HYM-42383-A01; München, Obermenzing, Premises of Zoologische Staatssammlung, 48.1648, 11.4849, 519 m, Malaise trap, 31.vii.2017, leg. Axel Hausmann, BIOUG42687-C04; BIOUG42697-G07; **Netherlands**: Noord Holland, duinreservaat Egmond aan Zee, ex. *Hemarisfuciformis*, 17.vii.2016, leg. M. R. Shaw, MRS_JFT0691; **United Kingdom**: England: Wiltshire, Bentley Woods, ex. *Hemaristityus*, 26.vi.2011, leg. M. Townesend, CNCHYM45325.

##### Geographical distribution.

PAL.

PAL: Austria*, Germany*, Netherlands*, United Kingdom.

##### Molecular data.

BIN: partially BOLD:AAA7143.

##### Host information.

Sphingidae: type reared from *Hemarisfuciformis* (Linnaeus, 1758); also Hemaristityus* (Linnaeus, 1758).

##### Notes.

**Figure 10.**
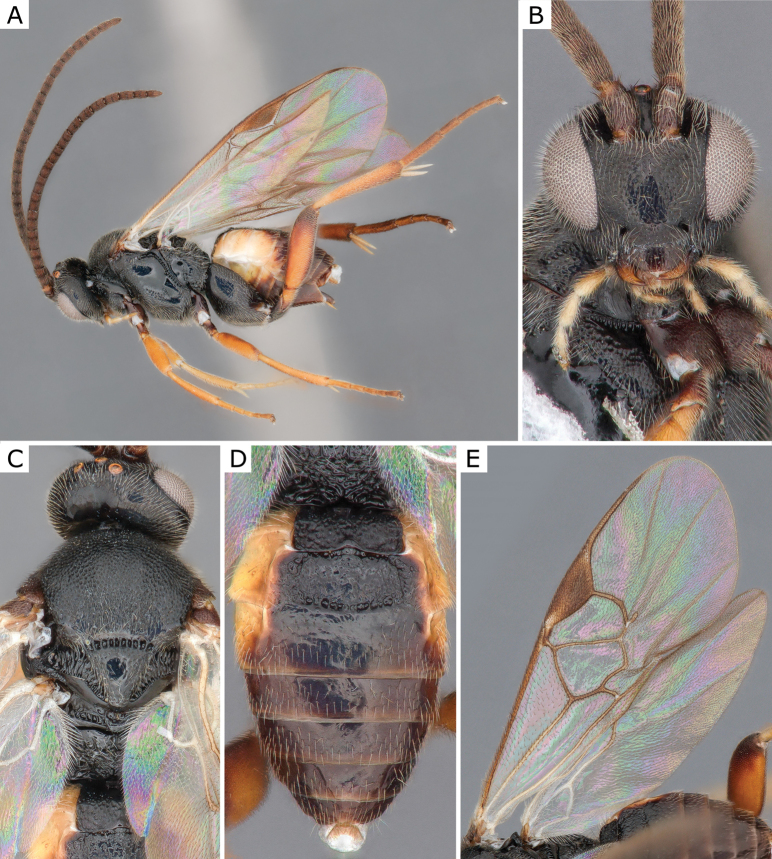
*Cotesiacoryphe* (Nixon, 1974), female (ZSM-HYM-33157-E11) **A** lateral view **B** head frontal view **C** mesosoma **D** metasoma **E** wing. Length of the specimen: 2.85 mm.

The German specimens were identified using Nixon (1974). Our sequences of this species were formerly part of BIN BOLD:ABY6805 and merged into BOLD:AAA7143 in February 2023. This BIN includes many clearly different species of *Cotesia*; see discussion below about this “megaBIN”. ASAP clustering resolves this species as a single cluster. One of the specimens that we examined (ZSM-HYM-ZLAB01-E11) and another specimen in this cluster (MRS_JFT0691) were reared from *Hemarisfuciformis*, which is congruent with the host information given in the original description of the species (Nixon 1974). A specimen from the United Kingdom (CNCHYM45325=MRS_JFT 0223) reared from *Hemaristityus* represents an additional host record for *Cotesiacoryphe*. This species is illustrated in Fig. 10.

#### ﻿ Cotesia eunomiae

Taxon classificationAnimaliaHymenopteraBraconidae

Shaw, 2009

21D0FAEA-2F16-554E-A63A-7F8EA864B338

##### Material examined.

**Belgium**: Luxembourg, Pisserotte, ex. *Boloriaeunomia*, vi.2004, leg. J. Choutt, individuals from separate gregarious broods, MS 106; MS 107; MS 108; **Finland**: Janakkala, ex. *Boloriaeunomia*, 14.vi.1992, leg. M. R. Shaw, MRS-JFT 0655; MRS-JFT 0656; **France**: Pyrénées-Orientales, Porte, ex. *Boloriaeunomia*, 30.v.2001, leg. T. Lafranchis, MRS_JFT 0118; **Germany**: Bavaria: Rhön, Hausen, Kleines Moor, 50.487, 10.039, 890 m, Malaise trap, 11.vii.2018, leg. D. Doczkal, ZSM-HYM-33165-E05.

##### Geographical distribution.

PAL.

PAL- Belgium, Finland*, France*, Germany*.

##### Molecular data.

BIN: BOLD:AAV9098.

##### Host information.

Nymphalidae: type reared from *Boloriaeunomia* (Esper, 1800).

##### Notes.

**Figure 11.**
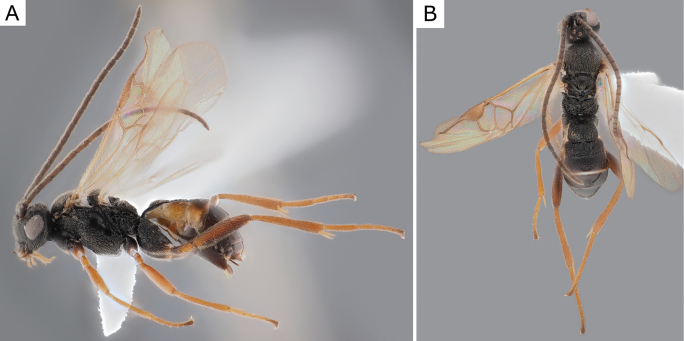
*Cotesiaeunomiae* Shaw, 2009, female (ZSM-HYM-33165-E05) **A** lateral and **B** dorsal views. Length of the specimen: 2.9 mm.

Our specimen from Germany matches the original description and clusters very closely (max. p-dist 0.34%) with six specimens (MRS-JFT 0655, MRS-JFT 0656, MS 106, MS 107, MS 108, MRS_JFT0118) reared from *Boloriaeunomia*, the host of the holotype (Shaw 2009). This wasp species appears to be completely specialised to *Boloriaeunomia*, which is classified as a highly endangered species in Bavaria and Germany (Reinhardt and Bolz 2011; Voith et al. 2016). As our German specimens are not reared, we checked the platform iNaturalist (https://www.inaturalist.org/) to verify whether this rather rare host occurs in this area. In a 5 km radius of our sampling site we found eight observations of *B.eunomia*. All these observations were confirmed by a lepidopterist via photos uploaded to iNaturalist. This species is illustrated in Fig. 11.

#### ﻿ Cotesia inducta

Taxon classificationAnimaliaHymenopteraBraconidae

Papp, 1973

61DC3578-57EB-5A8F-959F-20271DA23782

##### Material examined.

**Germany**: Bavaria: Oberstdorf, Oytal, rubble cone east of Gleitweg, 47.389, 10.348, 1200 m, Malaise trap, 16.vi.2014, leg. D. Doczkal, S. Schmidt, J. Voith, ZSM-HYM-33420-A02; **Spain**: Córdoba, Huerta El Caño, ex. *Leptotespirithous*, 15.vi.2012, leg. R. Obregón, MRS_JFT0268; Córdoba, Los Ídolos, ex. *Leptotespirithous*, 5.xii.2013, leg. R. Obregón, MRS_JFT0425; **United Kingdom**: England: Biggleswade, ex. *Satyriumw-album*, 01.vi.2005, leg. R. Revels, MS 005.

##### Geographical distribution.

PAL.

PAL- Bulgaria, Germany*, Hungary, Ireland, Israel, Korea, Moldova, Russia (KDA, PRI), Slovakia, Spain, Turkey, Ukraine, United Kingdom, Uzbekistan.

##### Molecular data.

BIN: BOLD:AAV9096.

##### Host information.

Host of type unknown; also Lycaenidae: *Callophrysavis* Chapman, 1909, *Celastrinaargiolus* (Linnaeus, 1758), *Glaucopsychemelanops* (Boisduval, 1828), Leptotespirithous* (Linnaeus, 1767), *Satyriumw-album* (Knoch, 1782), *Tomaresballus* (Fabricius, 1787).

##### Notes.

**Figure 12.**
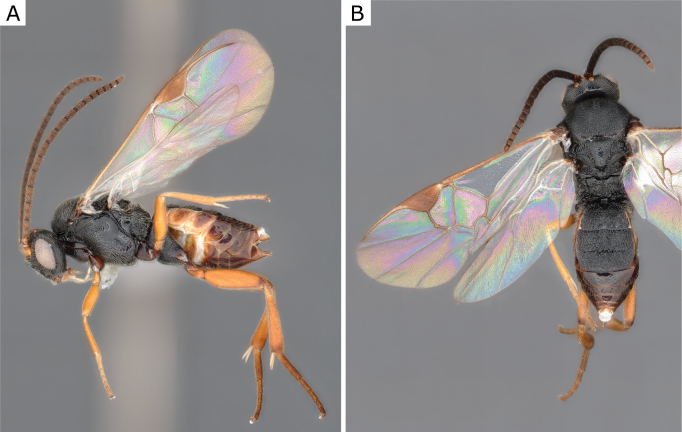
*Cotesiainducta* Papp, 1973, female (ZSM-HYM-33420-A02) **A** lateral and **B** dorsal views. Length of the specimen: 2.9 mm.

German specimens were compared with the description and keys in Papp (1973, 1986a, 1987) and Shaw (2007). No host was mentioned in the original description (Papp 1973) nor in later mentions of this species by its author (cf. Papp 1990). For more detailed and updated information on hosts and distribution of this species see Shaw (2007). Two specimens reared from *Leptotespirithous* (MRS_JFT0268, MRS_JFT0425) represent a new host record and cluster in the same BIN as our German specimens. This species is illustrated in Fig. 12.

#### ﻿ Cotesia mendicae

Taxon classificationAnimaliaHymenopteraBraconidae

(Tobias, 1986)

52A0F59E-E840-595A-86FB-3BC50CE0903C

##### Material examined.

**Austria**: Lower Austria, Raglitz, ex. *Phragmatobiafuliginosa*, 06.viii.2006, leg. J. Connell, MS 055; **Germany**: Bavaria: Balderschwang, Leiterberg, 47.489, 10.088, 1600 m, Malaise trap, 21.ix.2017, leg. D. Doczkal, J. Voith, ZSM-HYM-42326-A10.

##### Geographical distribution.

PAL.

PAL: Austria*, Germany*, Kazakhstan, Russia (VOR).

##### Molecular data.

BIN: partially BOLD:AAA7143.

##### Host information.

Host of (para-)type *Diaphoramendica* (Clerck, 1759); also Erebidae: Phragmatobiafuliginosa* (Linnaeus, 1758).

##### Notes.

**Figure 13.**
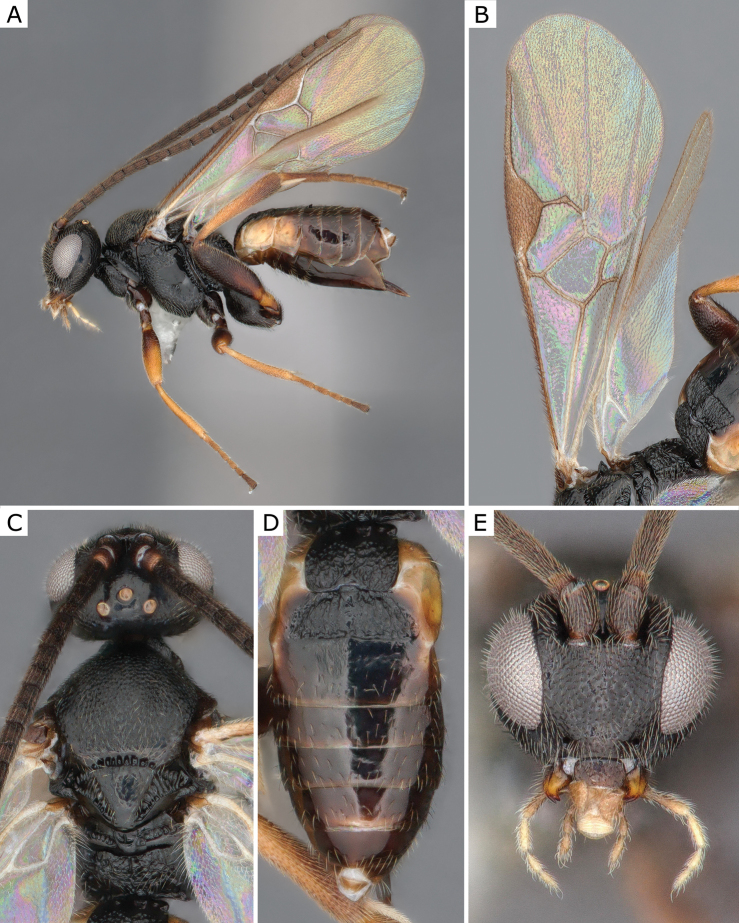
*Cotesiamendicae* (Tobias, 1986), female (ZSM-HYM-42326-A10) **A** lateral view **B** wing **C** mesosoma **D** metasoma **E** head frontal view. Length of the specimen: 2.85 mm.

Specimens were compared with the information provided in Tobias (1986) and Papp (1990). The specimen from Austria (MS 055) was compared to a paratype and reared from the same host group as the paratype. The COI barcode sequences of the Austrian and German specimens match 100% over a length of 616 bp and the specimens are very similar in morphology. This BIN includes many clearly different species of *Cotesia*; see discussion below about this “megaBIN”. ASAP clustering resolves the sequences of this species as a single cluster. No host is mentioned in the original description (Tobias 1986) but the paratype seen by Mark Shaw is labelled as from “*S.*” *mendica* [*Diaphoramendica* (Clerck, 1759)]. Here we present a related host record from *Phragmatobiafuliginosa* based on a gregarious brood from Austria (MS 055). Our sequences of this species are part of BIN BOLD:AAA71433. This species is illustrated in Fig. 13.

#### ﻿ Cotesia risilis

Taxon classificationAnimaliaHymenopteraBraconidae

(Nixon, 1974)

6E2E7505-627C-591B-A41D-434BC3A304E8

##### Material examined.

**Finland**: Uusimaa: Helsinki, Kaisaniemi Botanic Garden, 60.175700, 24.944700, Malaise trap, 29.viii-5.ix.2018, leg. J. Paukkunen, CNC1182785; **France**: Var, Callas, ex. *Satyriumw-album*, 5.v.2015, leg. P. Kan, B. Kan, MRS-JFT 0604; **Germany**: Baden-Württemberg: Malsch, Luderbusch, 48.913, 8.332, 117 m, Malaise trap, 26.vii.2020, leg. D. Doczkal, K. Grabow, ZSM-HYM-42388-G06; Bavaria: Bad Tölz, forest close to Isarstausee, 47.77, 11.547, 652 m, Malaise trap, 16.vii.2019, leg. J. Müller, ZSM-HYM-42378-F10; Chiemgau Alps, Ruhpolding, Fischbach, 47.709, 12.657, 720 m, Malaise trap, 30.viii.2016, leg. D. Doczkal, J. Voith, ZSM-HYM-33168-B10; Moos, Isarmündung, Magerrasen, swampy, 48.78, 12.966, 313 m, Malaise trap, 29.vii.2021, leg. GBOL3, R. Albrecht, ZSM-HYM-42396-E05; Moos, Isarmündung, 48.792, 12.968, 312 m, Malaise trap, 30.vi.2021, leg. GBOL3, R. Albrecht, ZSM-HYM-42395-B05; München, Fasanerie, Feldmoching, close to the train tracks, 48.193, 11.517, 509 m, Malaise trap, 9.vii.2019, leg. J. Müller, ZSM-HYM-42379-C09; Berchtesgaden National Park, Königssee, Rinnkendlsteig, 47.553, 12.964, 775 m, Malaise trap, 30.vii.2017, leg. D. Doczkal, J. Voith, ZSM-HYM-33161-B04; ZSM-HYM-33161-B05; 47.555, 12.965, 750 m, Malaise trap, 9.viii.2017, leg. D. Doczkal, J. Voith, ZSM-HYM-33161-H08; Neu-Geusmanns, Wald, 49.76, 11.48, 474 m, Malaise trap, 13.vii.2019, leg. J. Müller, ZSM-HYM-42378-B07; ZSM-HYM-42378-B08; **Spain**: Barcelona, Valles Oriental, St Pere de Vilamajour, ex. Gonepteryxcf.rhamni, 21.vi.2009, leg. C. Stefanescu, MS 095.

##### Geographical distribution.

PAL.

PAL- Finland*, France, Germany*, Greece, Hungary, Iran, Italy, Mongolia, Montenegro, Netherlands, Romania, Slovakia, Spain, Sweden, Turkey, United Kingdom.

##### Molecular data.

BIN: partially BOLD:AAA6099.

##### Host information.

Pieridae: type reared from *Gonepteryxrhamni* (Linnaeus, 1758); also Lycaenidae: *Satyriumw-album* (Knoch, 1782).

##### Notes.

Barcoding cluster BIN BOLD:AAA6099 currently includes 155 sequences which have been assigned seven species names: *Cotesiarisilis*, *C.saltatoria* (+ C.cf.saltatoria), *C.amesis*, *C.ancilla*, *C.cyaniridis*, *C.kazak*, and *C.flaviconchae*. The barcoding cluster also includes a large number of specimens from the Nearctic currently labelled as “Cotesia jft09”. Many of these species names are represented by reared material and, based on morphology and biology, clearly represent different species. They are all parasitoids of Pieridae and Lycaenidae with the exception of *C.kazak* (which might have been a misidentification) and possibly *C.flaviconchae*.

**Figure 14.**
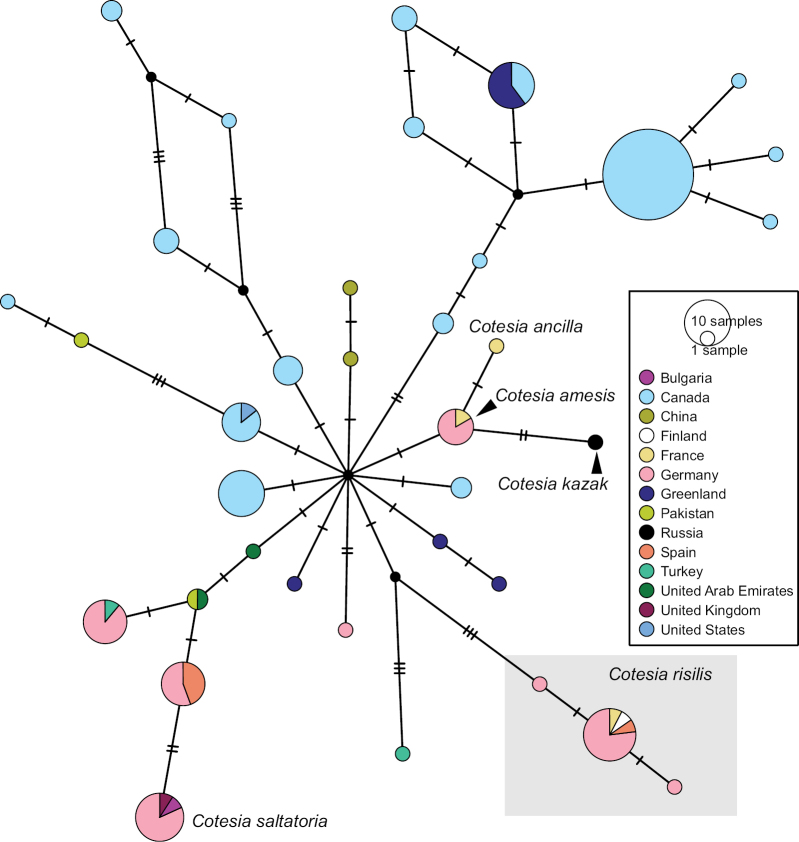
TCS haplotype network of BIN BOLD:AAA6099, sequence length for analysis: 504 bp. The haplotypes morphologically identified as *Cotesiarisilis* as part of this project are marked by a box. Each hatch mark in the network represents a single mutational change; small black dots at nodes indicate missing haplotypes. The diameter of the circles is proportional to the number of haplotypes sampled and the countries are colour-coded. The aligned sequences and traits can be reviewed in Suppl. materials 8, 9.

**Figure 15.**
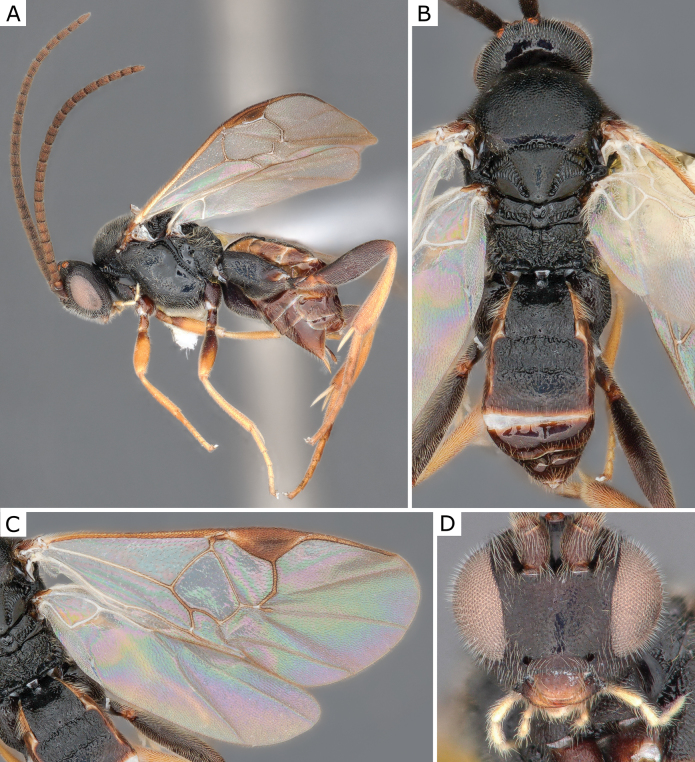
*Cotesiarisilis* (Nixon, 1974), female (ZSM-HYM-33161-B04) **A** lateral view **B** meso- and metasoma **C** wing **D** head frontal view. Length of the specimen: 3.35 mm.

We performed a Haplotype Network analysis including most sequences in this BIN (excluding specimens CNCHYM00406 (*C.cyaniridis*) and DQ538819 (*C.flaviconchae*) due to the sequences being significantly shorter than the other available sequences and with incomplete collection data). Our German material clusters in eight different haplotypes (Fig. 14) that include at least three different species identifications. However, we observed that some of our sequences (all voucher codes in material section of this species) are separated by at least four mutations from all other sequences in this BIN and 100% match the sequence of a specimen from Spain (MS 095) identified as *C.risilis* reared from Gonepteryxcf.rhamni, the host of the holotype. Morphological examination confirmed that our voucher specimens match the species concept of *C.risilis* (Nixon 1974). Distance matrix analysis of these specimens showed 0.31% intraspecific p-distance for *C.risilis*. Another specimen identified as *C.risilis* from France (the sequence of which is also part of this *C.risilis* haplotype) was reared from *Satyriumw-album* (MRS-JFT 0604). A host record from *Gonepteryxcleopatra* (Linnaeus, 1767) in Spain is called into doubt by Shaw and Colom (2023) as probably *G.rhamni*. This species is illustrated in Fig. 15.

#### ﻿ Cotesia selenevora

Taxon classificationAnimaliaHymenopteraBraconidae

Shaw, 2009

0F01FD36-A2DA-5202-AFCB-6081D28537AD

##### Material examined.

**Belgium**: Luxembourg: Libin, ex. *Boloriaselene*, 01.vi.2008, leg. J. Choutt, MRS-Cot-cal [paratype]; **Germany**: Bavaria: Lkr. Kelheim, Siegenburg, Bombodrom, 48.755, 11.791, 411 m, Malaise trap, 8.ix.2017, leg. D. Doczkal, J. Voith, ZSM-HYM-33169-A02.

##### Geographical distribution.

PAL.

PAL- Belgium, Finland, Germany*, Sweden.

##### Molecular data.

BIN: partially BOLD:AAA7143.

##### Host information.

Nymphalidae: type reared from *Boloriaselene* (Denis & Schiffermüller, 1775).

##### Notes.

**Figure 16.**
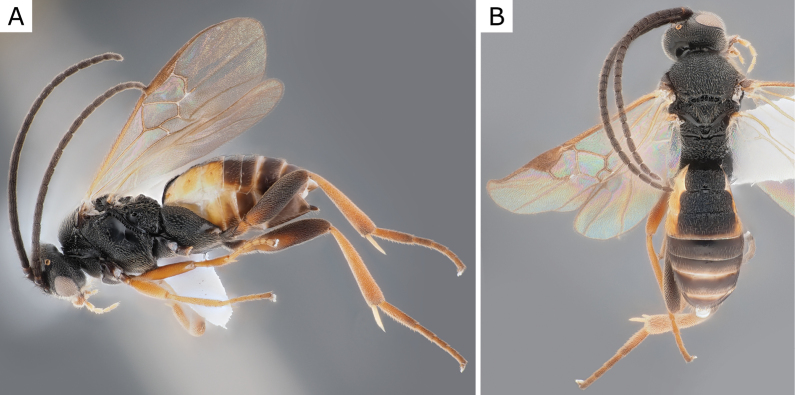
*Cotesiaselenevora* Shaw, 2009, female (ZSM-HYM-33169-A02) **A** lateral and **B** dorsal views. Length of the specimen: 3.0 mm.

Our sequences of this species were formerly part of BIN BOLD:AAA9381 and merged into BOLD:AAA7143 in February 2023. This BIN includes many clearly different species of *Cotesia*; see discussion below about this “megaBIN”. ASAP clustering resolves this species as a single cluster, including a specimen reared from *Boloriaselene* (Denis & Schiffermüller, 1775) and part of the type series (MRS-Cot-cal=MS 075). Our German specimen matches the barcode of the paratype 100% and also matches the species morphologically, based on Shaw (2009). This species is illustrated in Fig. 16.

#### ﻿ Cotesia subordinaria

Taxon classificationAnimaliaHymenopteraBraconidae

(Tobias, 1976)

3D3AA8DB-E900-59C5-BDB9-10EAFF963EC8

##### Material examined.

**Germany**: Bavaria: Plattling, Isarmündung, renat. gravel bar, 48.781, 12.906, 317 m, Malaise trap, 30.vi.2021, leg. GBOL3, R. Albrecht, ZSM-HYM-42393-H02; Waldbrunn, Stadtforst, 49.762, 9.803, 299 m, Malaise trap, 13.vii.2019, leg. J. Müller, ZSM-HYM-42385-G03; **Poland**: Biebrza National Park, 53.473694, 22.65675, ex. *Rivulasericealis*, 15.vi.2014, leg. M. R. Shaw, MRS_JFT0436; **United Kingdom**: England: Gloucestershire, Eastleach, ex. *Rivulasericealis*, 20.vii.2009, leg. M. R. Shaw, MS 082; 01.viii.2009, leg. M. R. Shaw, MS 102.

##### Geographical distribution.

PAL.

PAL: Azerbaijan, Georgia, Germany*, Netherlands, Poland*, Russia (NC), United Kingdom.

##### Molecular data.

BIN: partially BOLD:ACO3220.

##### Host information.

Host of type unknown; also Erebidae: *Rivulasericealis* (Scopoli, 1763).

##### Notes.

**Figure 17.**
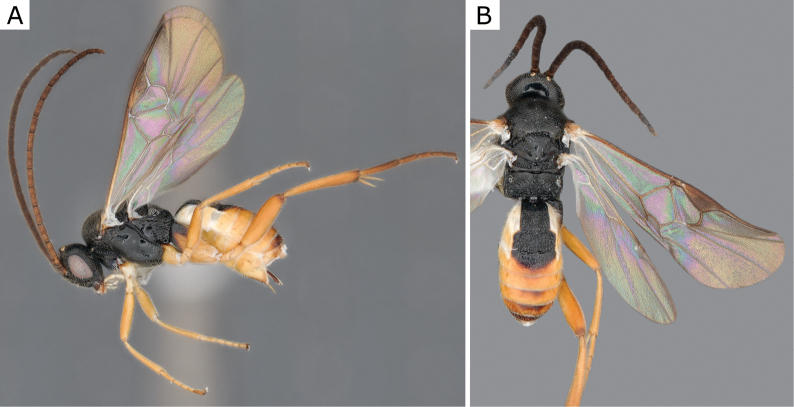
*Cotesiasubordinaria* (Tobias, 1976), female (ZSM-HYM-42393-H02) **A** lateral and **B** dorsal views. Length of the specimen: 3.1 mm.

German specimens were identified using the keys of Tobias (1986) and Papp (1986a). Sequences of three reared and identified specimens ex. *Rivulasericealis* detailed in Shaw (2012a, b) match our sequences from Germany at 0.16% max. p-distance: MRS_JFT0436, MS 082, MS 102. Our sequences of this species were formerly part of BIN BOLD:ACO3220 and merged into BOLD:AAA7143 in February 2023. This BIN includes many clearly different species of *Cotesia*; see discussion below about this “megaBIN”. ASAP clustering resolves this species as a single cluster. No host is mentioned in the original description (Tobias 1976), but we follow Shaw (2012a, 2012b) and the host data associated with those barcoded specimens. This species is illustrated in Fig. 17.

#### ﻿ Deuterixys plugarui

Taxon classificationAnimaliaHymenopteraBraconidae

(Tobias, 1975)

B6263A47-244A-5037-A8CB-847EAA0C785F

##### Material examined.

**Georgia**: Kakheti: Lagodekhi reserve, Mt Kudigora, 41.855850, 46.292733, 847 m, Malaise trap, 25.viii-4.ix.2014, leg. G. Japoshvili, CNC506818; **Germany**: Bavaria: Bad Windsheim, Rappenau, 49.482, 10.468, 382 m, canopy fogging, 9.vii.2020, leg. B. Leroy, ZSM-HYM-42392-G03; Bad Windsheim, 49.47, 10.446, 400 m, fogging, 19.v.2020, leg. B. Leroy, ZSM-HYM-42392-B01; ZSM-HYM-42392-B02; 49.488, 10.513, 411 m, canopy fogging, 20.v.2020, leg. B. Leroy, ZSM-HYM-42392-A10; Bibart, 49.657, 10.435, 325 m, canopy fogging, 7.vii.2020, leg. B. Leroy, ZSM-HYM-42392-D02; Schonungen, 50.075, 10.426, 341 m, canopy fogging, 20.v.2020, leg. B. Leroy, ZSM-HYM-42392-B06; Uffenheim, 49.544, 10.252, 357 m, canopy fogging, 3.vii.2019, leg. B. Leroy, ZSM-HYM-33159-B05; Wiesentheid, 49.803, 10.277, 216 m, canopy fogging, 2.vi.2019, leg. B. Leroy, ZSM-HYM-33158-E09; Wonfurt, 49.994, 10.408, 264 m, canopy fogging, 2.vii.2019, leg. B. Leroy, ZSM-HYM-33159-H10; **Netherlands**: Gelderland: Otterlo, Hoge Veluwe NP, ex. *Bucculatrixulmella*, 16.viii.2019, leg. M. R. Shaw, MRS_JFT0823; 24.viii.2019, leg. M. R. Shaw, MRS_JFT0826.

##### Geographical distribution.

PAL.

PAL- Georgia*, Germany*, Hungary, Moldova, Netherlands*, Russia (S), Ukraine, United Kingdom.

##### Molecular data.

BIN: BOLD:AEJ7518.

##### Host information.

Bucculatricidae: type reared from *Bucculatrixulmella* Zeller, 1848.

##### Notes.

**Figure 18.**
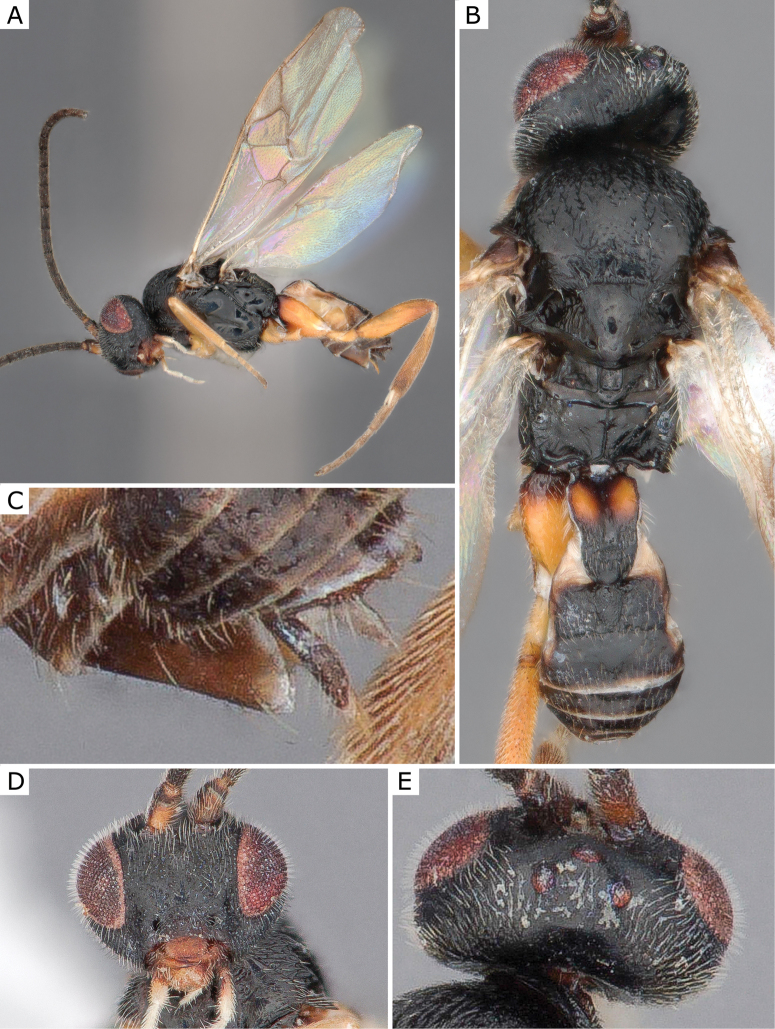
*Deuterixysplugarui* (Tobias, 1975), female (ZSM-HYM-42392-D02) **A** lateral view **B** meso- and metasoma **C** hypopygium lateral view **D** head frontal and **E** head dorsal views. Length of the specimen: 1.65 mm.

**Figure 19.**
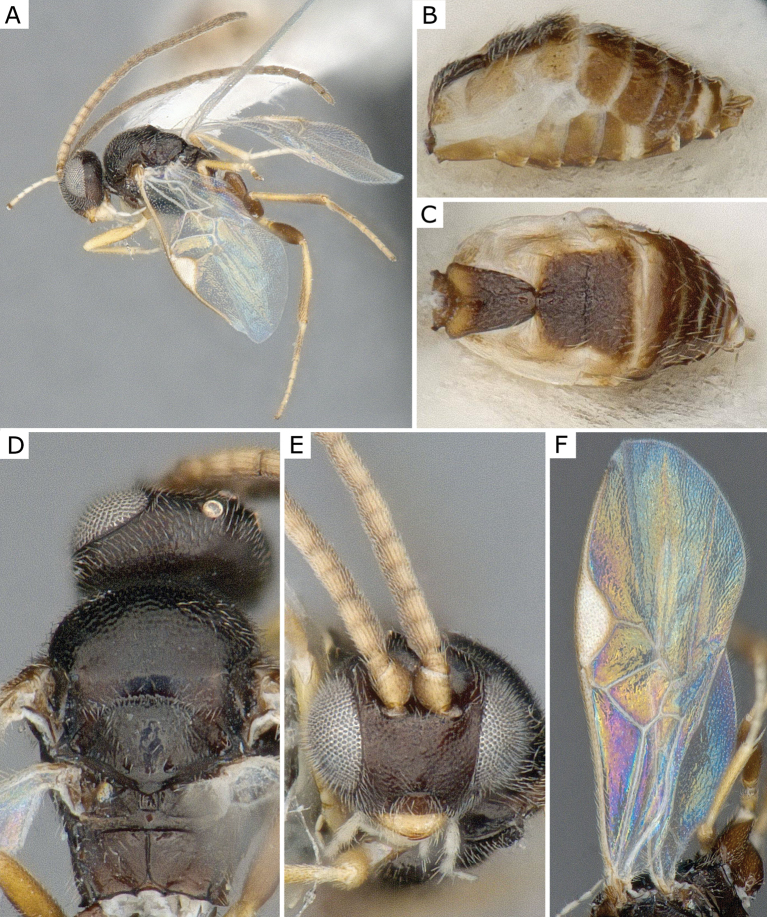
*Deuterixysplugarui* (Tobias, 1975), male (CNC506818) **A** lateral view **B** metasoma lateral view **C** metasoma dorsal view **D** mesosoma **E** head frontal view **F** wing.

German specimens were identified using the keys and information provided in Papp (1983), Tobias (1986), and especially Zeng et al. (2011). This species is matched with a DNA barcode for the first time. The only known host data is from the original description (Tobias 1975). Two specimens reared from *Bucculatrixulmella* were also available to us (MRS_JFT0823, MRS_JFT0826); they match our morphological concept of this species and were reared from the same host as the type, but have not yet been sequenced. This species is illustrated in Figs 18, 19.

#### ﻿ Dolichogenidea cerialis

Taxon classificationAnimaliaHymenopteraBraconidae

(Nixon, 1976)

9B40170F-E946-5E13-A0C7-102FB77B20A0

##### Material examined.

**Germany**: Baden-Württemberg: Malsch, Hansjakobstr. 7, Urban Garden, 48.884, 8.32, 120 m, Malaise trap, 13.ix.2020, leg. D. Doczkal, ZSM-HYM-33154-A05; Malsch, Luderbusch, 48.913, 8.332, 117 m, Malaise trap, 2.viii.2020, leg. D. Doczkal, K. Grabow, ZSM-HYM-42389-A11; 26.vii.2020, leg. D. Doczkal, K. Grabow, ZSM-HYM-42388-G11; ZSM-HYM-42388-H01; **Ukraine**: [translated and transcribed from Russian] Kaniv Nature Reserve, Shlehiv island, 4.ix.1991, leg. A. Kotenko, CNCHYM 01013.

##### Geographical distribution.

PAL.

PAL- Bulgaria, Germany*, Hungary, Israel, Italy, Kazakhstan, Russia (S), Spain, Ukraine*.

##### Molecular data.

BIN: BOLD:AAZ9570.

##### Host information.

Host of type unknown.

##### Notes.

**Figure 20.**
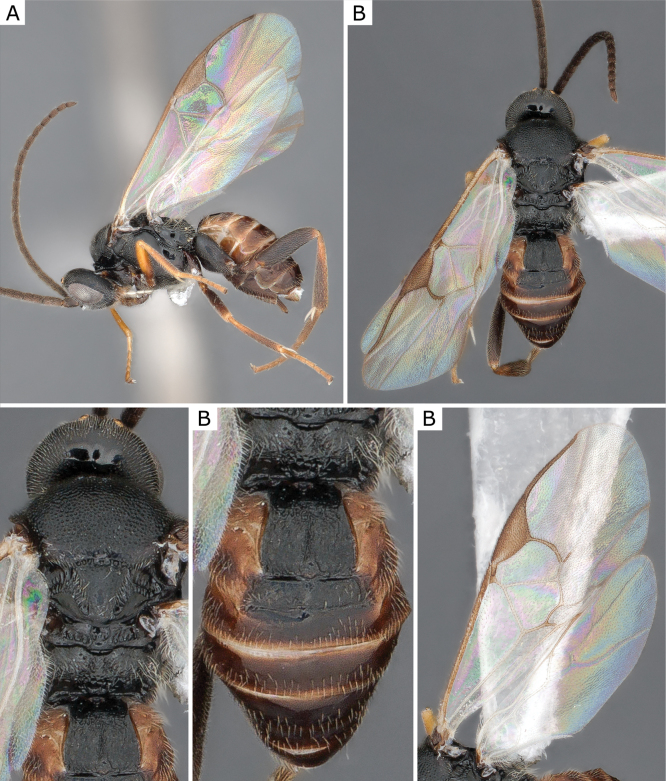
*Dolichogenideacerialis* (Nixon, 1976), female (ZSM-HYM-42388-G11) **A** lateral view **B** dorsal view **C** mesosoma **D** metasoma **E** wing. Length of the specimen: 2.4 mm.

**Figure 21.**
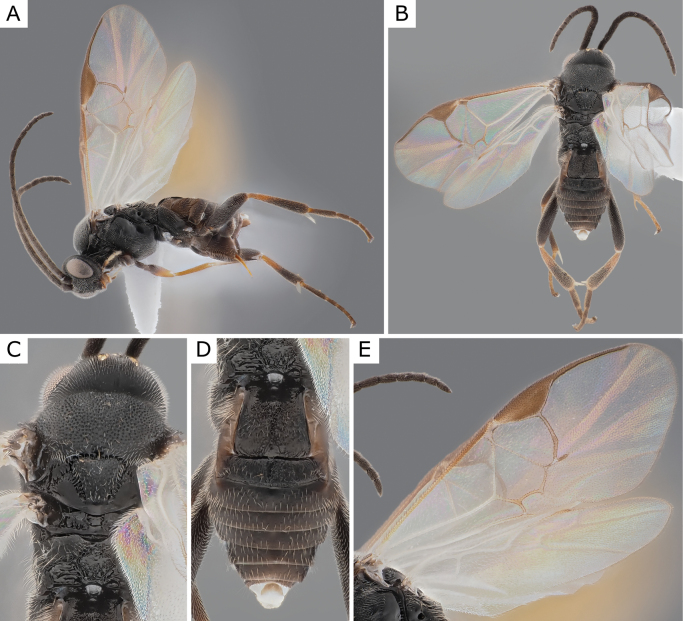
*Dolichogenideacerialis* (Nixon, 1976), female (ZSM-HYM-33154-A05) **A** lateral view **B** dorsal view **C** mesosoma **D** metasoma **E** wing. Length of the specimen: 2.4 mm.

The sequences of our German specimens match that of a specimen from Ukraine, identified by Kotenko and stored in the CNC collection (CNCHYM 01013); we studied both the German and the Ukrainian specimens and they match the morphological characters described by Nixon (1976), particularly the apical segment of the fore tarsus with a distinctive spine, the very short ovipositor sheaths (those two characters are very unusual in Dolichogenidea) but also leg colour, weak pale basal spot on pterostigma, anteromesoscutum punctuation, scutellum sculpture, propodeum areolation, hind spurs size, shape of T1 and T2. Nixon (1976) mentioned *Ascotisselenaria* (Denis & Schiffermüller, 1775) as host of additional non-type specimens from Israel which he identified as *Dolichogenideacerialis*, but at the same time noted that these reared specimens differed slightly in morphology from the type series. These specimens might represent a different species, so we consider this as a questionable host record for *D.cerialis*. This species is illustrated in Figs 20, 21.

#### ﻿ Dolichogenidea cheles

Taxon classificationAnimaliaHymenopteraBraconidae

(Nixon, 1972)

83EFD4CB-ACA3-5F49-ABFB-110A0F9E0314

##### Material examined.

**Germany**: Baden-Württemberg: Malsch, Hansjakobstr. 7, Urban Garden, 48.884, 8.32, 120 m, Malaise trap, 19.vii.2020, leg. D. Doczkal, ZSM-HYM-33154-G12; 5.vii.2020, leg. D. Doczkal, ZSM-HYM-33152-H03; ZSM-HYM-33152-H05; Bavaria: Moos, Isarmuendung, Hartholzauwald, 48.786, 12.959, 313 m, Malaise trap, 13.vii.2021, leg. GBOL3, R. Albrecht, ZSM-HYM-42394-F11; ZSM-HYM-42394-F12; 29.vii.2021, leg. GBOL3, R. Albrecht, ZSM-HYM-42394-G09; Sielstetten, östlich Grafendorfer Forst, 48.578, 11.863, 520 m, Malaise trap, 16.vii.2019, leg. J. Müller, ZSM-HYM-42383-A03.

##### Geographical distribution.

PAL.

PAL- Finland, Germany*, Hungary, Poland, Russia (NW), Sweden, Turkey.

##### Molecular data.

BIN: BOLD:ACQ9527.

##### Host information.

Host of type unknown. Other host associations in need of verification.

##### Notes.

**Figure 22.**
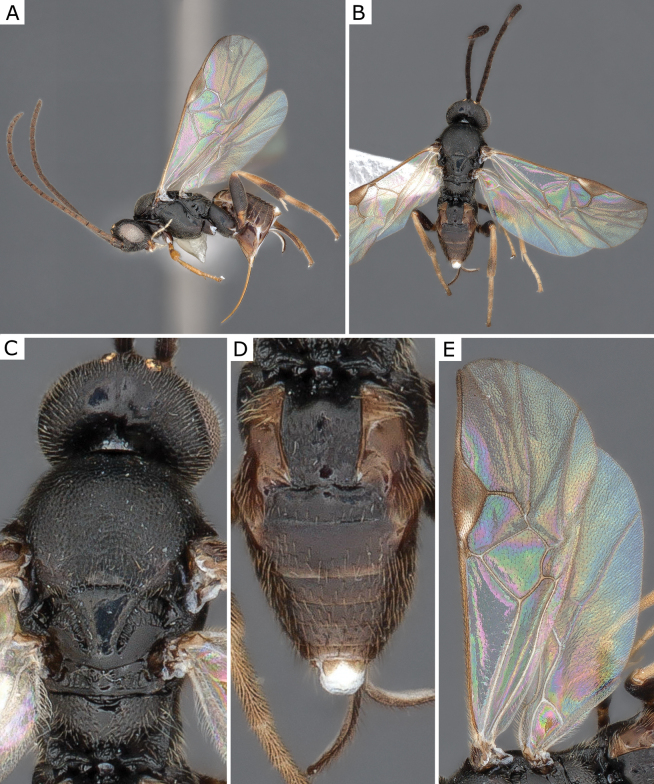
*Dolichogenideacheles* (Nixon, 1972), female (ZSM-HYM-33152-H03) **A** lateral view **B** dorsal view **C** mesosoma **D** metasoma **E** wing. Length of the specimen: 2.3 mm.

German specimens were compared with the original description (Nixon 1972) as well as the works of Papp (1978) and Tobias (1986), and they match perfectly the characters provided in Nixon’s original description, particularly: colour of legs, tegula, pterostigma, flagellomeres (more or less, the paler areas in flagellomeres are present but not as sharp as described by Nixon), ocelli in very low triangle, anteromesoscutum and scutellum sculpture, propodeum sculpture (lack of carinae, as described by Nixon), metatibial spurs, density and shape of spines on outer surface of metatibia, T1 and T2 shape and sculpture, length of ovipositor sheaths, partial widening of sheaths towards posterior end, and down-curved ovipositor. The hosts that in the past have been associated with this species are not from the type material and comprise two different Lepidoptera families: Tortricidae – *Aclerisholmiana* (Linnaeus, 1758) (Papp 1988), and Gracillariidae – *Caloptiliarufipennella* (Hübner, 1796) (Papp 1988) and *Caloptiliafribergensis* (Fritzsche, 1871) (Marczak and Buszko 1993); therefore, we consider supposed hosts in need of verification. This species is illustrated in Fig. 22.

#### ﻿ Dolichogenidea coleophorae

Taxon classificationAnimaliaHymenopteraBraconidae

(Wilkinson, 1938)

D68A100E-60EB-5C4F-AD7A-36BBE25C773C

##### Material examined.

**Canada**: Newfoundland and Labrador: 2 miles west of Gambo, 48.789478, -54.261043, 19.vi.1975, leg. A. G. Raske, J. D. Rowe, ex. *Coleophoraserratella*, CNCHYM 01020; Gambo, 48.786481, -54.215467, 27.vii.1985, CNCHYM 01019; **Germany**: Baden-Württemberg: Malsch, Luderbusch, 48.912, 8.332, 112 m, Malaise trap, 24.v.2020, leg. D. Doczkal, K. Grabow, ZSM-HYM-42386-G05; Bavaria: Forchheim, Untere Mark bei Willersdorf, 49.739, 10.969, 261 m, Malaise trap, 12.vii.2019, leg. J. Müller, ZSM-HYM-42377-D11; [exact location unknown], 13.viii.1975, CNCHYM 01021; **Switzerland**: Aigle, 46.319083, 6.970444, 9.viii.1973, CNCHYM 01024; **United Kingdom**: England: [exact location unknown], 15.iii.1938, ex. *Coleophoraserratella*, CNCHYM 01023.

##### Geographical distribution.

NEA, PAL.

NEA: Canada (NL); PAL: Azerbaijan, Finland, Germany*, Hungary, Poland, Romania, Russia (KHA, VOR, YAR), Slovakia, Switzerland, Tajikistan, Tunisia, Turkey, United Kingdom, Uzbekistan.

##### Molecular data.

BOLD:AEO8197.

##### Host information.

Coleophoridae: type reared from *Coleophoraserratella* (Linnaeus, 1761); also possibly Coleophora?ibipennella Zeller, 1849; Coleophora?lusciniaepennella (Treitschke, 1833); Coleophora?obducta (Meyrick, 1931); Coleophora?tadzhikiella Danilevsky, 1955.

##### Notes.

**Figure 23.**
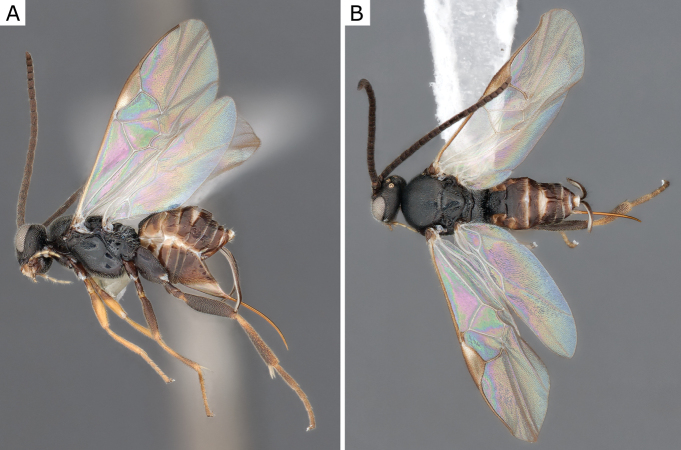
*Dolichogenideacoleophorae* (Wilkinson, 1938), female (ZSM-HYM-42377-D11) **A** lateral and **B** dorsal views. Length of the specimen: 2.25 mm.

Several Canadian (CNCHYM 01019, CNCHYM 01020) and European specimens at the CNC (CNCHYM 01021 from Germany, CNCHYM 01023 from United Kingdom, CNCHYM 01024 from Switzerland) were reared from the host of the type or the synonym *Coleophorafuscedinella* Zeller, 1849 and identified as *D.coleophorae*. We compared our material collected in Germany with those reared specimens collected in Canada and Europe as well as the original description and the works of Nixon (1976), Papp (1981a) and Tobias (1986). Our freshly collected material from Germany morphologically matches the reared specimens as well as the literature. All specimens we examined are associated with sequences, except for CNCHYM 01023 which was collected in 1938. The barcode sequences of the reared specimens are short (107 bp) and all of these specimens were collected between 1973 and 1985. They do not 100% match our sequences (3 bp difference). However, since the COI sequences of the historical material are very short, the species occurs in several neighbouring countries of Germany, the host is widely distributed in Europe and occurs in Germany, and our specimens match the morphological concept of the species, we conclude that our specimens fit our current concept of this species despite the currently somewhat conflicting DNA barcodes. In the historical literature there are other host records from several additional species of *Coleophora*, which may be correct but are here cited as questionable. Other literature host records from different Lepidoptera families are much less probable and we do not consider them here. This species is illustrated in Fig. 23.

#### ﻿ Glyptapanteles indiensis

Taxon classificationAnimaliaHymenopteraBraconidae

(Marsh, 1979)

314EDEE8-7D95-5E2E-BFD9-89A7B1E7B921

##### Material examined.

**Czech Republic**: South Moravia: Obora Soutok, Lanžhot, 48.69, 16.945, 165 m, 14.v.2013, leg. P. Drozd, BC-ZSM-HYM-23872-A04; ex. ?*Alsophilaauscularia*, 05.v.2015, leg. M. Sigut, BC-ZSM-HYM-27569-F09; ex. *Operophterabrumata*, 08.v.2015, leg. P. Drozd, BC-ZSM-HYM-23872-F08; **Germany**: Baden-Württemberg: Malsch, Hansjakobstr. 7, Urban Garden, 48.884, 8.32, 120 m, Malaise trap, 11.x.2020, leg. D. Doczkal, ZSM-HYM-33154-B09; Bavaria: Markt Nordheim, Kehrenberg, 49.547, 10.366, 419 m, canopy fogging, 10.vii.2020, leg. B. Leroy, ZSM-HYM-42393-B07; Rhön Fladungen, NSG Schwarzes Moor, Kermi-Hochmoor, 50.512, 10.069, 780 m, Malaise trap, 23.viii.2017, leg. D. Doczkal, ZSM-HYM-33165-A08; Rhön Hausen, Eisgraben, basalt block heap at forest edge, 50.503, 10.09, 735 m, Malaise trap, 23.vii.2018, leg. D. Doczkal, ZSM-HYM-33166-C03; 9.viii.2018, leg. D. Doczkal, ZSM-HYM-33166-D10; Südpark, 48.103, 11.509, 550 m, ex. *Operophterabrumata*, 28.v.2021, leg. W. Langer, ZSM-HYM-ZLAB01-F05; **India**: Kashmir Sprinagar, ex. *Lymantriaobfuscata*, [collector unknown], 2.v.1977, CNCHYM 03231; CNCHYM 03232; **Japan**: Aichi: Mt. Chausu, 35.2275 137.655558, 1300 m, 9.vii.1995, leg. K. Yamagishi, JMIC 0011.

##### Geographical distribution.

NEA, OTL, PAL.

NEA: USA (PA), OTL: India, PAL: Czech Republic*, Germany*, Japan*.

##### Molecular data.

BOLD:ABY2372.

##### Host information.

Erebidae: type reared from *Lymantriaobfuscata* (Marsh, 1979); also *Lymantriadispar* (Linnaeus, 1758); Geometridae*: Operophterabrumata* (Linnaeus, 1758).

##### Notes.

**Figure 24.**
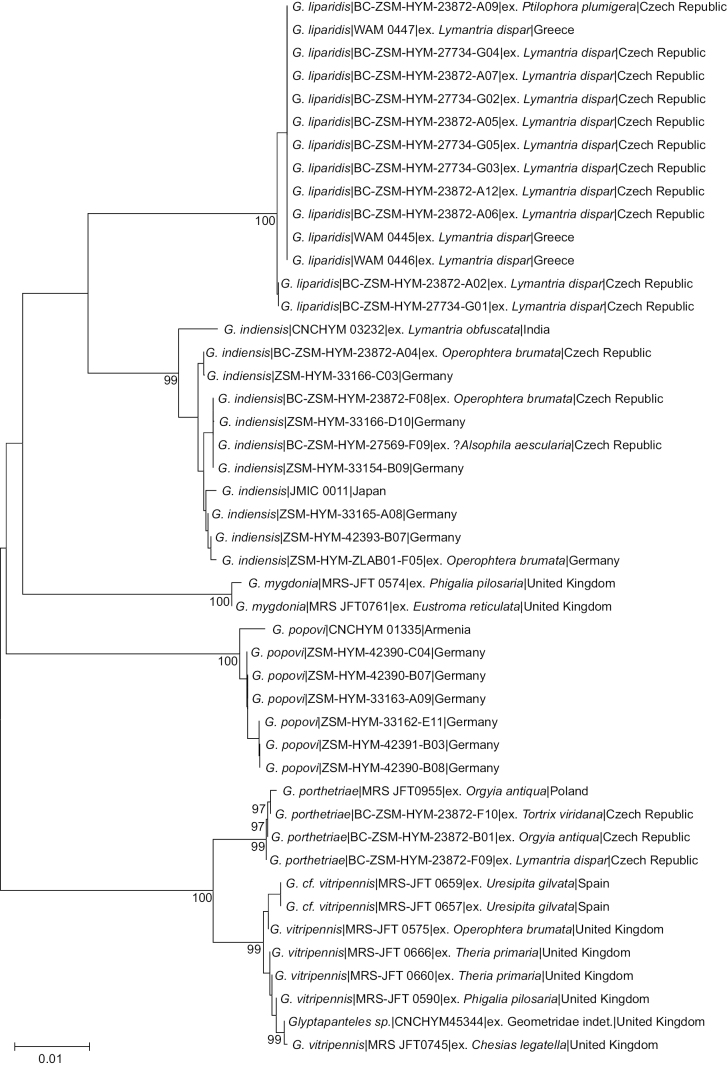
Neighbour-joining topology of the COI barcoding region of *Glyptapantelesindiensis*, *G.popovi* and morphologically similar species, based on Kimura 2-parameter distances. Numbers next to nodes represent non-parametric bootstrap values > 90% (1,000 replicates). The aligned sequences and N-J topology can be reviewed in Suppl. materials 6, 7.

**Figure 25.**
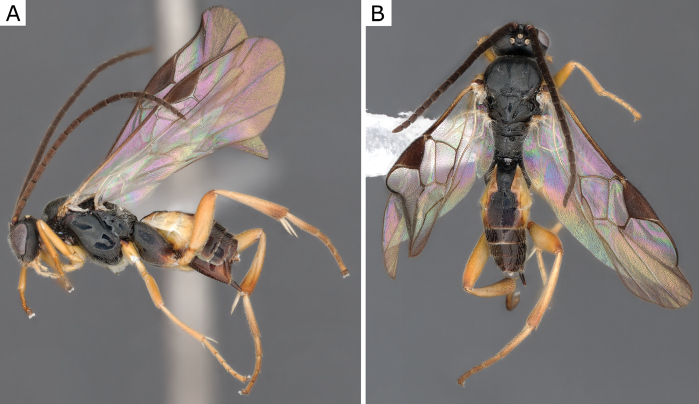
*Glyptapantelesindiensis* (Marsh, 1979), female (ZSM-HYM-33154-B09) **A** lateral and **B** dorsal views. Length of the specimen: 3.5 mm.

**Figure 26.**
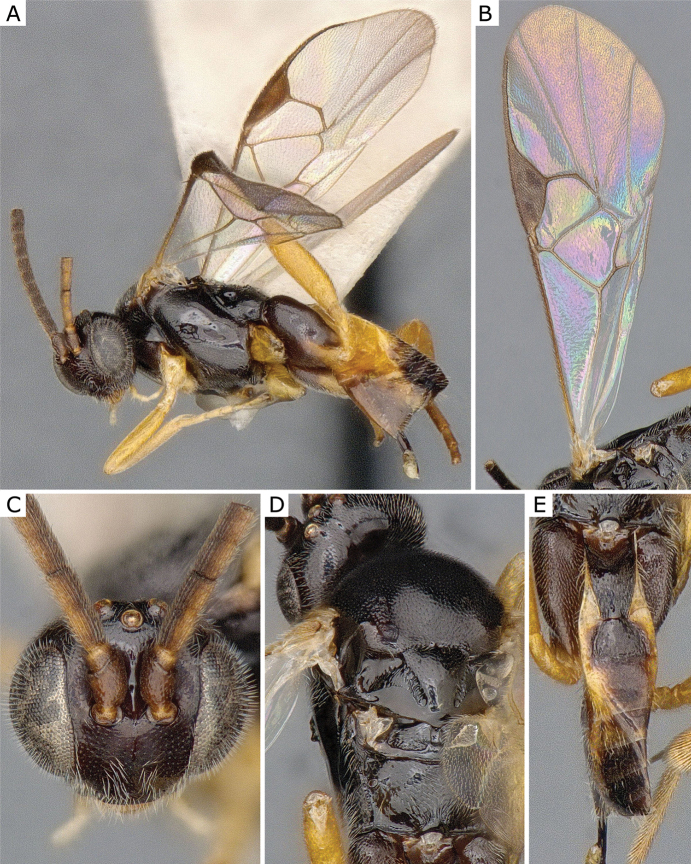
*Glyptapantelesindiensis* (Marsh, 1979), female paratype (CNCHYM 03231) **A** lateral view **B** forewing **C** head frontal view **D** mesosoma **E** metasoma.

We record *G.indiensis* for the first time in the Palaearctic region, based on specimens from Germany, Japan and Czech Republic. This species is morphologically similar, especially in habitus, to several *Glyptapanteles* species. Our identification was therefore based on a careful study (detailed below) which included a combination of morphology (see Figs 25, 26, both examination of authenticated specimens and consulting original descriptions and other relevant papers (e.g., Muesebeck 1928; Nixon 1973; Marsh 1979; Papp 1983)), DNA barcodes (available for all species discussed below, see Suppl. materials and Fig. 24) and hosts (available for most species mentioned above, except for *G.popovi*). The German, Japanese (JMIC 0011) and Czech specimens (BC-ZSM-HYM-23872-A04, BC-ZSM-HYM-23872-F08, BC-ZSM-HYM-27569-F09) were identified by morphological comparison with two paratypes of *G.indiensis* deposited in the CNC as well as information from the original description (Marsh 1979). One of those paratypes (a male specimen (CNCHYM 03232) reared from *Lymantriaobfuscata* in India, apparently part of the same brood as the female paratype deposited at the CNC (CNCHYM 03231)) was successfully barcoded and the 455 bp sequence matches the remaining sequences in this BIN by 99.5%. *Glyptapantelesindiensis* is known to parasitise *Lymantriaobfuscata* in India and *Lymantriadispar* (at least in the laboratory), and therefore is of interest as a biocontrol agent (Marsh 1979), although we are not aware of published data confirming the parasitisation of *L.dispar* by *G.indiensis* in the wild. However, we have additional data from a metabarcoding study in Germany including caterpillars that were collected as part of a canopy fogging project. There, seven individual caterpillars of *L.dispar* had more than 30 reads of sequences that match the barcoding cluster that we associated with *G.indiensis* (99.6–100% bp similarity), therefore providing at least an indirect confirmation of the parasitisation of *L.dispar* in the wild by this wasp species. Additionally, we examined two specimens (ZSM-HYM-ZLAB01-F05 and BC-ZSM-HYM-23872-F08) which were reared from the Geometridae*Operophterabrumata* and represent a new host family record for this species. The aforementioned metabarcoding data suggests that there might be additional hosts for this species; however, this would need to be confirmed by rearing.

Other species of *Glyptapanteles* parasitising *Lymantriadispar* are *Glyptapantelesliparidis* (BOLD:AAV2164, including several reared specimens from this host such as WAM 0445=MRS_JFT 0028, BC-ZSM-HYM-23872-A02, BC-ZSM-HYM-23872-A05) and *Glyptapantelesporthetriae* (BOLD:ACL7229, including a reared specimen from this host: BC-ZSM-HYM-23872-F09). The many available barcodes from both *G.porthetriae* and *G.liparidis* are very distinct and clearly separated from those of *G.indiensis* (> 3.5% K2P-distance, see Fig. 24), and there are also morphological differences between these three species as detailed in Marsh (1979).

The known hosts of *G.mygdonia* include *Operophterabrumata* and the multiple hosts recorded for *G.vitripennis* in the literature (many of them likely incorrect) include both *O.brumata* and *L.dispar*. The many available sequences of *G.mygdonia* (BOLD:AAU5027) and *G.vitripennis* (BOLD:AAA7148) are also very distinctive and far apart from those of *indiensis* [The sequences of *G.vitripennis* and *G.liparidis* are relatively very close (2.13% p-distance); furthermore, *G.vitripennis* seems to include a complex of species that remains unresolved, but that is beyond the scope of the present paper]. There are also morphological differences between these species and *G.indiensis* (Nixon 1973). Two characters we found were useful are the relative length of the ovipositor sheaths, which is much longer in *G.indiensis* as compared to the other two species and the lack of a curved spine on the fore tarsus for *G.indiensis* (present in both *G.mygdonia* and *G.vitripennis*).

The last species we compared to *G.indiensis* was *G.popovi*, which is much less understood. Until now, *G.popovi* was only known from Turkey and Turkmenistan (Fernandez-Triana et al. 2020) and there is no host known for this species. Based on information from Telenga (1955), Tobias (1986), and the study of a specimen of *G.popovi* from Armenia (CNCHYM 01335) identified by Kotenko in 1981 and deposited in the CNC these two species are different. Analyses of available DNA sequences strongly support that (these two species are clearly apart by more than 5% K2P-distance, compare Fig. 25). We found German specimens from both species and thus both species are recorded from Germany in this paper (see also comments below, under *G.popovi*). *Glyptapantelesindiensis* is illustrated in Figs 25, 26.

#### ﻿ Glyptapanteles popovi

Taxon classificationAnimaliaHymenopteraBraconidae

(Telenga, 1955)

63763F81-99D8-5126-8AD2-F5706E4AFAC8

##### Material examined.

**Armenia**: [translated and transcribed from Russian] Khosrov Forest State Reserve, Vediiskii reservoir sector (of reserve), montane forest, 30.vi.1981, CNCHYM 01335; **Germany**: Bavaria: Garmisch-Partenkirchen, Zugspitze, Platt, 47.406, 11.009, 1965 m, Malaise trap, 11.ix.2018, leg. D. Doczkal, J. Voith, ZSM-HYM-33163-A09; 47.407, 11.006, 2030 m, Malaise trap, 11.ix.2018, leg. D. Doczkal, J. Voith, ZSM-HYM-42391-B03; 47.407, 11.008, 2005 m, Malaise trap, 11.ix.2018, leg. D. Doczkal, J. Voith, ZSM-HYM-42390-B07; ZSM-HYM-42390-B08; 9.x.2018, leg. D. Doczkal, J. Voith, ZSM-HYM-42390-C04; 47.412, 11.007, 2210 m, Malaise trap, 2.viii.2018, leg. D. Doczkal, J. Voith, ZSM-HYM-33162-E11.

##### Geographical distribution.

PAL.

PAL- Armenia*, Germany*, Turkey, Turkmenistan.

##### Molecular data.

BIN: BOLD:AEJ4298.

##### Host information.

Host unknown.

##### Notes.

**Figure 27.**
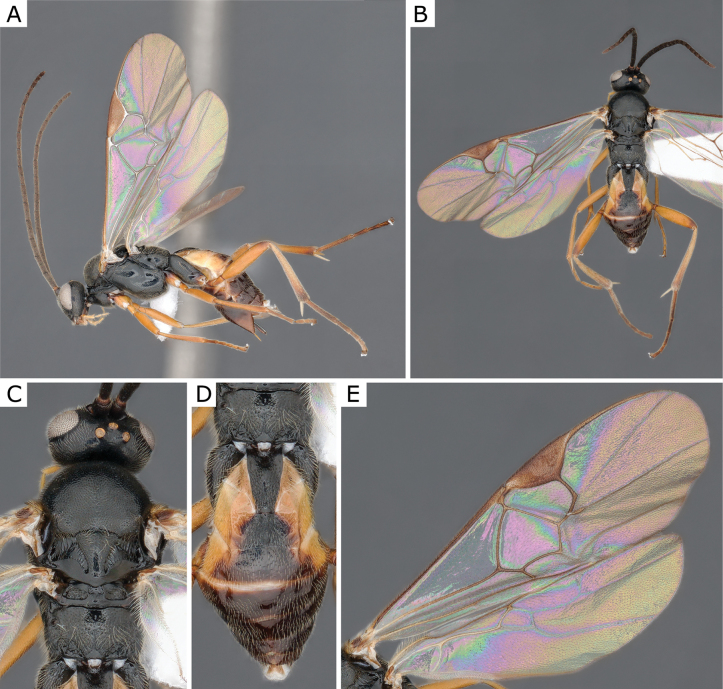
*Glyptapantelespopovi* (Telenga, 1955), female (ZSM-HYM-33163-A09) **A** lateral view **B** dorsal view **C** mesosoma **D** metasoma **E** wing. Length of the specimen: 4.0 mm.

**Figure 28.**
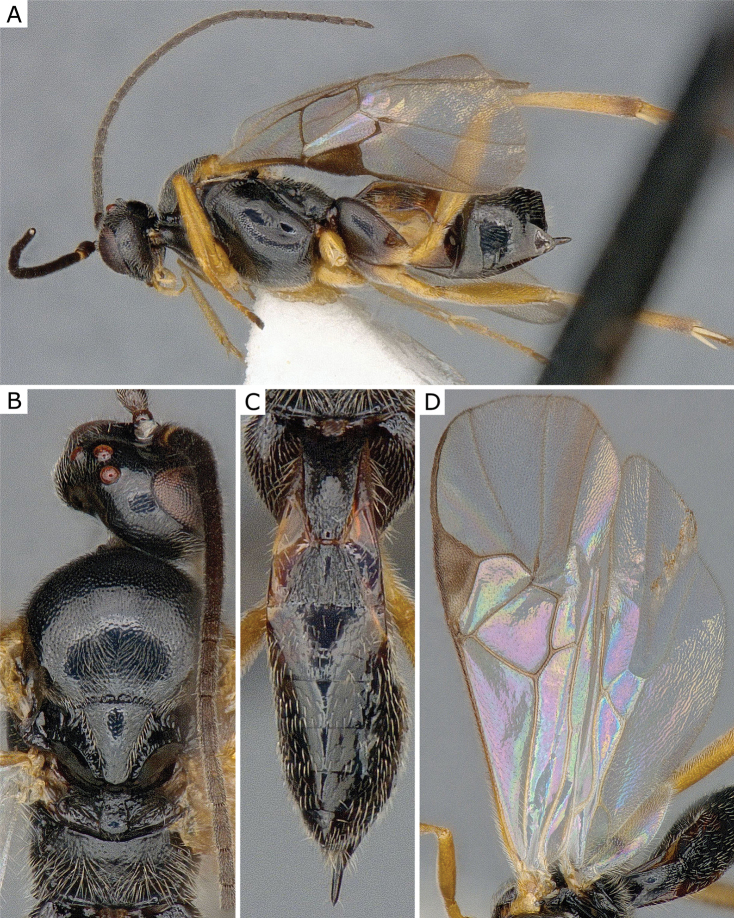
*Glyptapantelespopovi* (Telenga, 1955), female (CNCHYM 01335=CNC280989) **A** lateral view **B** mesosoma **C** metasoma **D** wing.

Our specimens were identified morphologically using keys and information in Telenga (1955), Papp (1983), and Tobias (1986) as well as comparison with a specimen from Armenia (CNCHYM 01335) identified by Kotenko in 1981 and deposited in the CNC. The German and Armenian specimens also share similar DNA barcodes (99.5% overlap, sequence length of Armenian specimen is 425 bp). See also comments under *G.indiensis* above. Our material of this species was collected only in an alpine habitat (> 1900 m, Zugspitze). This species is illustrated in Figs 27, 28.

#### ﻿ Illidops cloelia

Taxon classificationAnimaliaHymenopteraBraconidae

(Nixon, 1965)

5CF81CB1-FA59-5BEC-BD81-734A631BCA31

##### Material examined.

**Germany**: Bavaria: Garmisch-Partenkirchen, Zugspitze, Platt, 47.407, 11.008, 2005 m, Malaise trap, 2.viii.2018, leg. D. Doczkal, J. Voith, ZSM-HYM-42389-G08.

##### Geographical distribution.

PAL.

PAL- Austria, Germany*, Hungary, Korea, Russia (E, NC), Slovakia, Switzerland, Tajikistan, former Yugoslavia.

##### Molecular data.

BIN: BOLD:AEO8223.

##### Host information.

Host unknown.

##### Notes.

**Figure 29.**
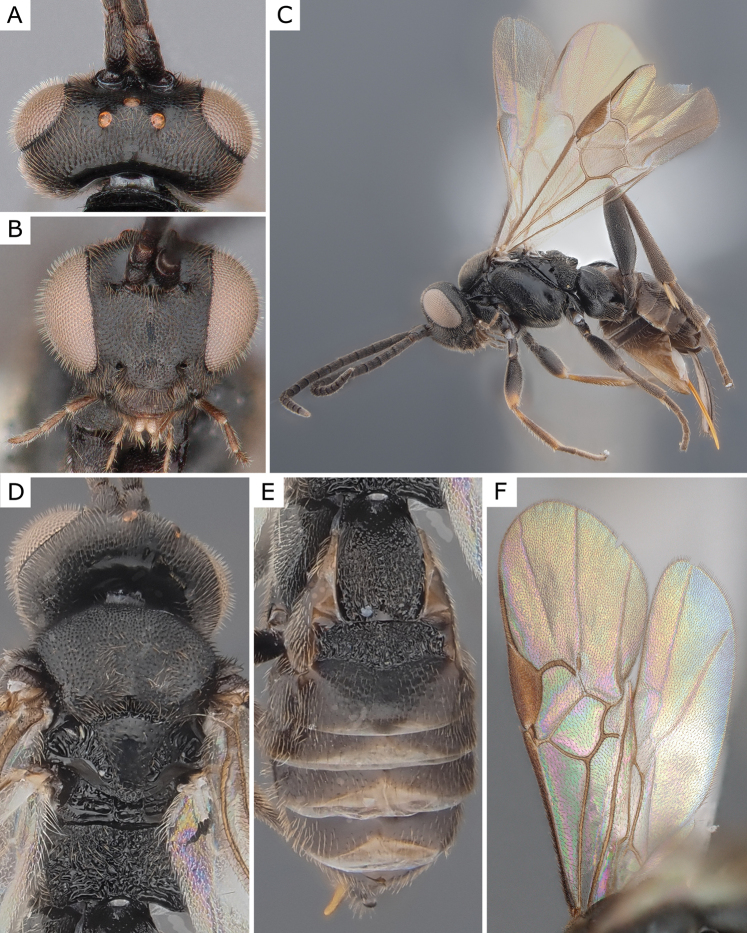
*Illidopscloelia* (Nixon, 1965), female (ZSM-HYM-42389-G08) **A** head dorsal view **B** head frontal view **C** lateral view **D** mesosoma **E** metasoma **F** wing. Length of the specimen: 2.75 mm.

The German specimen was identified by comparison with the keys and details from the works of Nixon (1965, 1976), Papp (1973, 1981a), and Tobias (1986). Our material of this species was collected only in an alpine habitat (> 2000 m, Zugspitze). This species is illustrated in Fig. 29.

#### ﻿ Illidops splendidus

Taxon classificationAnimaliaHymenopteraBraconidae

(Papp, 1974)

BA87A911-A360-5F1A-9D43-A86498C4A290

##### Material examined.

**Germany**: Bavaria: Lkr. Kelheim Siegenburg, Bombodrom, 48.755, 11.791, 411 m, Malaise trap, 26.v.2017, leg. D. Doczkal, J. Voith, ZSM-HYM-33168-H06.

##### Geographical distribution.

PAL.

PAL- Germany*, Hungary, Russia (C).

##### Molecular data.

BIN: BOLD:AEJ7519.

##### Host information.

Host unknown.

##### Notes.

**Figure 30.**
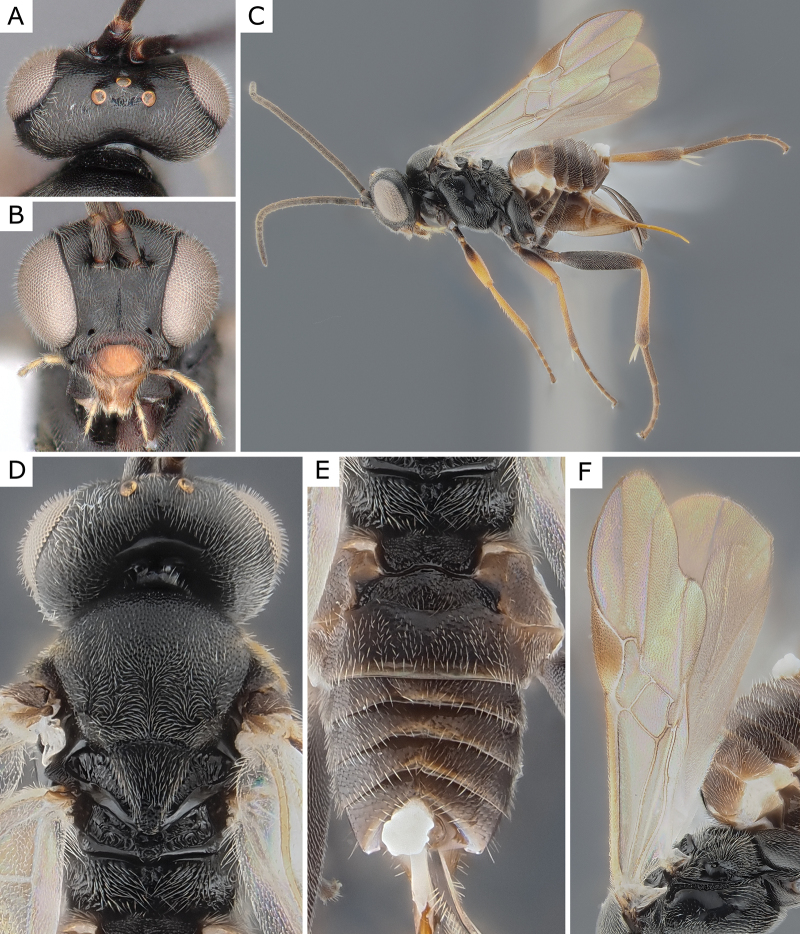
*Illidopssplendidus* (Papp, 1974), female (ZSM-HYM-33166-H06) **A** head dorsal view **B** head frontal view **C** lateral view **D** mesosoma **E** metasoma **F** wing. Length of the specimen: 2.75 mm.

The German specimen was identified by comparison with the keys of Papp (1974, 1981a) and Tobias (1986) and the original description by Papp (1974). The single specimen available to us was collected in a rare sand-dune habitat close to Siegenburg in Bavaria. This species is illustrated in Fig. 30.

#### ﻿ Microgaster arctostaphylica

Taxon classificationAnimaliaHymenopteraBraconidae

Shaw, 2012

1C3CF7A4-07F1-530C-A158-F560404FD224

##### Material examined.

**Germany**: Bavaria: Bad Tölz, forest close to Isarstausee, 47.77, 11.547, 652 m, Malaise trap, 16.vii.2019, leg. J. Müller, ZSM-HYM-42378-F09; Berchtesgaden National Park, Königssee, Rinnkendlsteig, 47.553, 12.964, 775 m, Malaise trap, 14.vi.2017, leg. D. Doczkal, J. Voith, ZSM-HYM-33160-F10; ZSM-HYM-33160-F12; Oberndorf, close to Krebsbach, 49.868, 9.516, 342 m, Malaise trap, 13.vii.2019, leg. J. Müller, ZSM-HYM-42325-C09; Rhön Fladungen, NSG Schwarzes Moor, Kermi-Hochmoor, 50.512, 10.069, 780 m, Malaise trap, 18.vii.2017, leg. D. Doczkal, ZSM-HYM-33164-G03; **Sweden**: Gotland: Roleks; Gotlands kommun, 57.536783, 18.337883, Malaise trap, 17.vii-9.viii.2005, leg. SMTP, CNC471954; **United Kingdom**: Scotland: Inverness-shire, Tulloch Moor, ex. *Argyroplocearbutella*/*Sticteamygindiana*, 30.iv.2016, leg. R. J. Heckford, MRS_JFT0770; Morayshire, Boat of Garton NH9319, ex. ?*Stictea mygdeana*, 20.v.2014, leg. R. J. Heckford, MRS-JFT 0640.

##### Geographical distribution.

PAL.

PAL- Germany*, Sweden*, United Kingdom.

##### Molecular data.

BIN: BOLD:AAH1039.

##### Host information.

Tortricidae: type reared from *Argyroplocearbutella* (Linnaeus, 1758); also *Epinotianemorivaga* (Tengström, 1848), *Sticteamygindiana* (Denis & Schiffermüller, 1775).

##### Notes.

**Figure 31.**
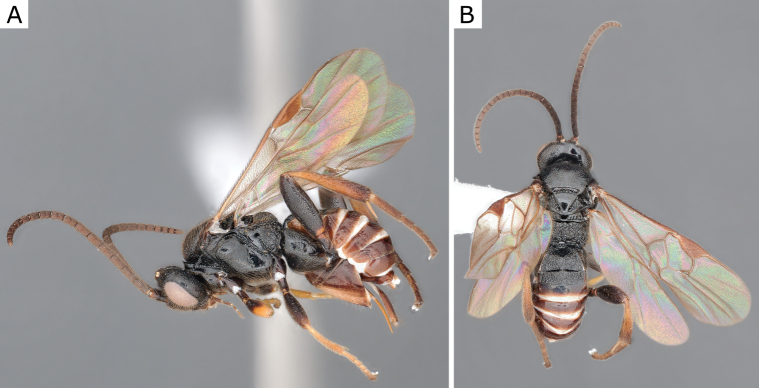
*Microgasterarctostaphylica* Shaw, 2012, female (ZSM-HYM-33160-F12) **A** lateral and **B** dorsal views. Length of the specimen: 3.45 mm.

German specimens were identified by comparison with the original description and specimens from that paper (Shaw 2012a); all specimens we examined except for one had the orange (paler) tip of mesofemur that is considered one of the diagnostic features. The species can be confused with *Microgastermessoria* Haliday, 1834. However, *M.messoria* clusters in BIN BOLD:AAV2150 (including a reared specimen ex. *Aspilapteryxtringipennella* (Zeller, 1839) in the BOLD database, one of the hosts that Nixon based his concept on (of *Microgastertibialis* Nees, 1834, a synonym of *M.messoria*)). It is well separated from the BIN that contains our sequences of *M.arctostaphylica* (BOLD:AAV2150: within-BIN max. p-distance: 0.86%, Nearest-Neighbour minimum p-distance: 2.31%). Based on publicly available data in BOLD, *M.arctostaphylica* also could be present in Turkey (CGTURK-1139) but we could not examine that specimen. This BIN is quite variable (2.25% max. within-BIN max. p-distance and 2.69% min. p-distance to the Nearest-Neighbor). There is a single specimen (ZSM-HYM-42325-C09) that is 2.3% apart from the other German material and clusters with the specimen from Turkey; except for some minor differences in colouration it matches our morphological concept of the species. Future analyses and study of more specimens might provide support to consider this a complex of species, but for the time being we consider all of them to belong to *Microgasterarctostaphylica*. This wasp species is known to parasitise several Tortricidae hosts, all of which were collected feeding on *Arctostaphylosuva-ursi* (Shaw, 2012). Two reared specimens from Scotland were sequenced and clustered in the same BIN (BOLD:AAH1039), one from ?*Sticteamygindiana* (MRS-JFT 0640), and one from *Argyroplocearbutella*/*Sticteamygindiana* (MRS_JFT0770). This species is illustrated in Fig. 31.

#### ﻿ Microgaster caris

Taxon classificationAnimaliaHymenopteraBraconidae

Nixon, 1968

8D8726EA-DD3E-5ADE-AD1B-3DCB06688120

##### Material examined.

**Germany**: Bavaria: Allgäu, Oberstdorf, Oytal Magerweide östlich Oytalhaus, 47.388, 10.344, 1056 m, 1.vi.2014, leg. D. Doczkal, S. Schmidt, J. Voith, BC-ZSM-HYM-24118-E02; Atzmannsberg, Hessenreuther und Atzmannsberger Forst, 49.825, 11.963, 550 m, Malaise trap, 11.vii.2019, leg. J. Müller, ZSM-HYM-42384-B05; Bad Toelz, forest close to Isarstausee, 47.77, 11.547, 652 m, Malaise trap, 16.vii.2019, leg. J. Müller, ZSM-HYM-42378-G03; Berchtesgaden, Bischofswiesener Ache, 47.629, 12.975, 597 m, Malaise trap, 20.vii.2019, leg. J. Müller, ZSM-HYM-42375-F10; Berchtesgaden, Königssee, Wald west of St. Bartholomae, 47.547, 12.965, 620 m, Malaise trap, 14.vii.2017, leg. D. Doczkal, J. Voith, ZSM-HYM-42323-C08; Dienhausen, 47.886, 10.827, 724 m, Malaise trap, 15.vii.2019, leg. J. Müller, ZSM-HYM-42380-A03; Fabrikschleichach, close to Weilersbachtal, 49.917, 10.525, 408 m, Malaise trap, 12.vii.2019, leg. J. Müller, ZSM-HYM-42376-D12; Ketterschwang, Wald, 47.963, 10.676, 650 m, Malaise trap, 16.vii.2019, leg. J. Müller, ZSM-HYM-42381-F10; Marktoberdorf, nördlich von Rieder, 47.76, 10.643, 769 m, Malaise trap, 15.vii.2019, leg. J. Müller, ZSM-HYM-42384-H07; Moos, Isarmündung, Hartholzauwald, 48.786, 12.959, 313 m, Malaise trap, 25.viii.2021, leg. GBOL3, R. Albrecht, ZSM-HYM-42395-A10; Moos, Isarmündung, Magerrasen, swampy, 48.78, 12.966, 313 m, Malaise trap, 12.viii.2021, leg. GBOL3, R. Albrecht, ZSM-HYM-42396-E09; 25.viii.2021, leg. GBOL3, R. Albrecht, ZSM-HYM-42396-F07; Moos, Isarmündung, *Molinia* meadow, 48.779, 12.95, 313 m, Malaise trap, 12.viii.2021, leg. GBOL3, R. Albrecht, ZSM-HYM-42394-H03; 13.vii.2021, leg. GBOL3, R. Albrecht, ZSM-HYM-42394-B03; 16.vi.2021, leg. GBOL3, R. Albrecht, ZSM-HYM-42391-D03; 25.viii.2021, leg. GBOL3, R. Albrecht, ZSM-HYM-42394-E10; 29.vii.2021, leg. GBOL3, R. Albrecht, ZSM-HYM-42394-C09; 30.vi.2021, leg. GBOL3, R. Albrecht, ZSM-HYM-42391-E09; ZSM-HYM-42391-E10; Moos, Isarmündung, Weichholz Auwald, 48.792, 12.968, 312 m, Malaise trap, 13.vii.2021, leg. GBOL3, R. Albrecht, ZSM-HYM-42395-B12; 25.viii.2021, leg. GBOL3, R. Albrecht, ZSM-HYM-42395-C01; Berchtesgaden National Park, Wald west of St.Bartholomä, 47.547, 12.965, 620 m, Malaise trap, 21.viii.2017, leg. D. Doczkal, J. Voith, ZSM-HYM-33156-F04; ZSM-HYM-33156-F05; Neu-Geusmanns, Wald, 49.76, 11.48, 474 m, Malaise trap, 13.vii.2019, leg. J. Müller, ZSM-HYM-42378-B05; Plattling, Isarmündung, renat. gravel bar, 48.781, 12.906, 317 m, Malaise trap, 25.viii.2021, leg. GBOL3, R. Albrecht, ZSM-HYM-42391-B11; ZSM-HYM-42391-B12; 30.vi.2021, leg. GBOL3, R. Albrecht, ZSM-HYM-42393-H01; Rothenbuch, 49.963, 9.389, 346 m, Malaise trap, 15.vii.2019, leg. J. Müller, ZSM-HYM-42382-C02; ZSM-HYM-42382-C03; Ruhpolding, Fischbach, 47.709, 12.657, 720 m, Malaise trap, 4.vii.2016, leg. D. Doczkal, J. Voith, ZSM-HYM-42398-C10; 47.716, 12.658, 710 m, Malaise trap, 13.ix.2016, leg. D. Doczkal, J. Voith, ZSM-HYM-42323-A03; Siegenburg, 48.755, 11.791, 411 m, Malaise trap, 13.vii.2017, leg. D. Doczkal, J. Voith, ZSM-HYM-42324-D06; Wimmelbach, close to Untere Mark, pond edge, 49.71, 10.994, 290 m, Malaise trap, 12.vii.2019, leg. J. Müller, ZSM-HYM-42381-C12.

##### Geographical distribution.

PAL.

PAL- Austria, China (JL), Czech Republic, Germany*, Hungary, Russia (C, PR), Slovakia, Switzerland.

##### Molecular data.

BIN: BOLD:ACN6851.

##### Host information.

Host of type unknown. Other host associations in need of verification.

##### Notes.

**Figure 32.**
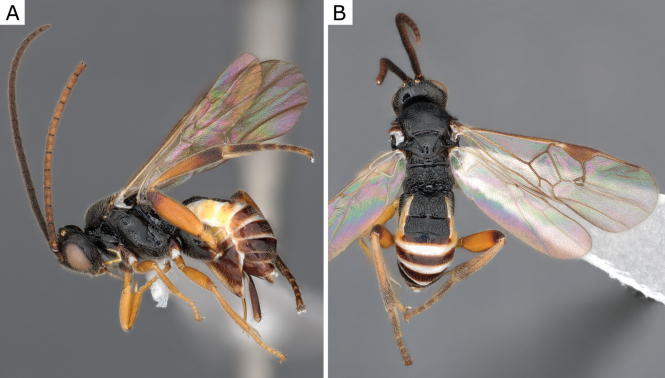
*Microgastercaris* Nixon, 1968, female (ZSM-HYM-33156-F04) **A** lateral and **B** dorsal views. Length of the specimen: 3.55 mm.

The German specimens were identified by comparison with the original description (Nixon 1968) as well as information from Papp (1976a). The host data associated with this wasp species (?Gelechiidae: ?*Anacampsispopulella* (Clerck, 1759); ?Tortricidae: ?*Archipsrosana* (Linnaeus, 1758)) was reported in later publications without enough detail and thus it is here considered to be questionable. This species is illustrated in Fig. 32.

#### ﻿ Microgaster nervosae

Taxon classificationAnimaliaHymenopteraBraconidae

Shaw, 2023

E05B3AA6-442F-5969-8579-D0B6A227EB8D

##### Material examined.

**Germany**: Baden-Württemberg: Malsch, Luderbusch, 48.912, 8.332, 114 m, Malaise trap, 12.iv.2020, leg. D. Doczkal, K. Grabow, ZSM-HYM-42386-G09; Bavaria: Bodenwöhr, Truppenübungsplatz, 49.264, 12.358, 395 m, Malaise trap, 22.v.2016, leg. D. Doczkal, J. Voith, ZSM-HYM-42397-H05; ZSM-HYM-42397-H06; Lkr. Kelheim Siegenburg, Bombodrom, 48.755, 11.791, 411 m, Malaise trap, 26.v.2017, leg. D. Doczkal, J. Voith, ZSM-HYM-33168-H04; ZSM-HYM-33168-H05; 48.759, 11.809, 407 m, Malaise trap, 26.v.2017, leg. D. Doczkal, J. Voith, ZSM-HYM-33168-E09; ZSM-HYM-33168-E10; Siegenburg, Bombodrom, 48.755, 11.791, 411 m, Malaise trap, 14.vi.2017, leg. D. Doczkal, J. Voith, ZSM-HYM-42324-C06; **United Kingdom**: Scotland: Edinburgh, Blackford Hill, ex. *Agonopterixnervosa*, 23.iv.2019, leg. M. R. Shaw, MRS_JFT0801 [paratype]; 01.v.2019, leg. M. R. Shaw, MRS_JFT0802 [paratype].

##### Geographical distribution.

PAL.

PAL- Germany*, United Kingdom.

##### Molecular data.

BIN: BOLD:ACR4142.

##### Host information.

Depressariidae: type reared from *Agonopterixnervosa* (Haworth, 1811); also *Agonopterixumbellana* (Fabricius, 1794).

##### Notes.

**Figure 33.**
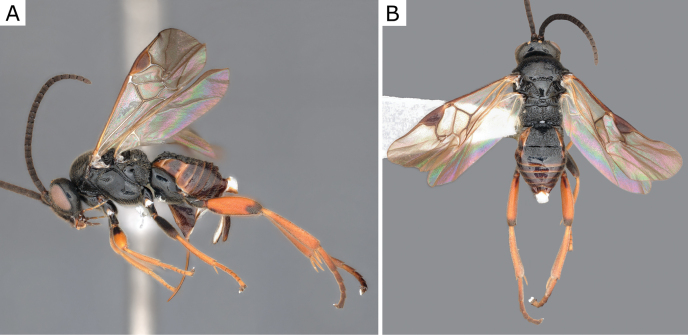
*Microgasternervosae* Shaw, 2023, female (ZSM-HYM-33168-E09) **A** lateral and **B** dorsal views. Length of the specimen: 4.0 mm.

This species was very recently described from Britain (Shaw 2023) and German specimens were compared morphologically with the description. Our sequences 100% match the sequences of two paratypes reared from *Agonopterixnervosa* (MRS_JFT0801, MRS_JFT0802). This species is illustrated in Fig. 33.

#### ﻿ Microgaster nixalebion

Taxon classificationAnimaliaHymenopteraBraconidae

Shaw, 2004

86F10391-4B8C-56FB-937D-27EE3718405B

##### Material examined.

**Austria**: Lower Austria: Opponitz, ex. *Pataniaruralis*, vi.2007, leg. J. Connell, MRS_JFT0934; **Germany**: Baden-Württemberg: Malsch, Hansjakobstr. 7, Urban Garden, 48.884, 8.32, 120 m, Malaise trap, 13.ix.2020, leg. D. Doczkal, ZSM-HYM-33153-H06; Malsch, Hardtwald NE Kieswerk Glaser, 48.915, 8.313, 125 m, Malaise trap, 9.vii.2011, leg. D. Doczkal, ZSM-HYM-42323-D11; Malsch, Luderbusch, 48.913, 8.332, 117 m, Malaise trap, 2.viii.2020, leg. D. Doczkal, K. Grabow, ZSM-HYM-42389-A08; 26.vii.2020, leg. D. Doczkal, K. Grabow, ZSM-HYM-42388-F12; Bavaria: Böbing, Ammertal, 47.747, 10.965, 686 m, 14.vii.2013, leg. D. Doczkal, ZSM-HYM-33420-D06; Dienhausen, 47.886, 10.827, 724 m, Malaise trap, 15.vii.2019, leg. J. Müller, ZSM-HYM-42325-C12; ZSM-HYM-42325-D01; ZSM-HYM-42380-A05; ZSM-HYM-42380-A06; ZSM-HYM-42380-A07; ZSM-HYM-42380-A08; Fabrikschleichach, Lichtung, 49.918, 10.56, 366 m, Malaise trap, 12.vii.2019, leg. J. Müller, ZSM-HYM-42382-F04; ZSM-HYM-42382-F05; Fabrikschleichach, close to Weilersbachtal, 49.917, 10.525, 408 m, Malaise trap, 12.vii.2019, leg. J. Müller, ZSM-HYM-42376-D10; ZSM-HYM-42376-D11; ZSM-HYM-42376-E01; Gütersleben, Gramschatzer Wald, 49.873, 9.932, 272 m, Malaise trap, 12.vii.2019, leg. J. Müller, ZSM-HYM-42379-F08; Lkr. Kelheim Abensberg-Sandharlanden, NSG Sandharlandener Heide, 48.845, 11.801, 376 m, Malaise trap, 3.viii.2017, leg. D. Doczkal, J. Voith, ZSM-HYM-33157-F07; Lkr. Kelheim Siegenburg, Bombodrom, 48.755, 11.791, 411 m, Malaise trap, 8.ix.2017, leg. D. Doczkal, J. Voith, ZSM-HYM-33168-H09; Lohr am Main, Beilstein, Weinberg Waldrand, 50.003, 9.563, 195 m, Malaise trap, 14.vii.2018, leg. D. Doczkal, ZSM-HYM-33156-A01; 3.vi.2018, leg. D. Doczkal, ZSM-HYM-33155-E07; Moos, Isarmündung, *Molinia* meadow, 48.779, 12.95, 313 m, Malaise trap, 30.vi.2021, leg. GBOL3, R. Albrecht, ZSM-HYM-42391-E06; Moos, Isarmündung, Stromtalwiese, 48.777, 12.994, 310 m, Malaise trap, 13.vii.2021, leg. GBOL3, R. Albrecht, ZSM-HYM-42395-E03; München, NSG Allacher Lohe, 48.199, 11.475, 502 m, Malaise trap, 19.viii.2021, leg. GBOL3, R. Albrecht, ZSM-HYM-42326-F04; 23.vi.2021, leg. GBOL3, R. Albrecht, ZSM-HYM-42326-B01; Berchtesgaden National Park, Königssee, Rinnkendlsteig, 47.551, 12.964, 695 m, Malaise trap, 9.viii.2017, leg. D. Doczkal, J. Voith, ZSM-HYM-33162-B05; Berchtesgaden National Park, Wald west of St.Bartholomä, 47.547, 12.965, 620 m, Malaise trap, 28.vi.2017, leg. D. Doczkal, J. Voith, ZSM-HYM-33156-D11; ZSM-HYM-33156-D12; ZSM-HYM-33156-E01; ZSM-HYM-33156-E02; Rhön Fladungen, NSG Schwarzes Moor, Kermi-Hochmoor, 50.512, 10.069, 780 m, Malaise trap, 9.viii.2017, leg. D. Doczkal, ZSM-HYM-33165-A02; Rhön Hausen, Kleines Moor, 50.487, 10.039, 890 m, Malaise trap, 25.vii.2018, leg. D. Doczkal, ZSM-HYM-33165-F06; Sankt Wolfgang, Wald, 48.466, 13.146, 474 m, Malaise trap, 13.vii.2019, leg. J. Müller, ZSM-HYM-42384-A03; Schärding, 48.436, 13.41, 304 m, Malaise trap, 13.vii.2019, leg. J. Müller, ZSM-HYM-42325-D06; ZSM-HYM-42325-D07; ZSM-HYM-42375-E09; Selb, Schönwald, 50.187, 12.1, 675 m, Malaise trap, 14.vii.2019, leg. J. Müller, ZSM-HYM-42381-F04; Siegenburg, Bombodrom, 48.755, 11.791, 411 m, Malaise trap, 13.vii.2017, leg. D. Doczkal, J. Voith, ZSM-HYM-42324-D04; 23.viii.2017, leg. D. Doczkal, J. Voith, ZSM-HYM-42324-G09; 29.vi.2017, leg. D. Doczkal, J. Voith, ZSM-HYM-42324-E12; Thiersheim, Karlmühle, 50.075, 12.152, 517 m, Malaise trap, 15.vii.2019, leg. J. Müller, ZSM-HYM-42381-A02; ZSM-HYM-42381-A05; Volkach, Kolitzheim, 49.922, 10.234, 229 m, Malaise trap, 16.vii.2019, leg. J. Müller, ZSM-HYM-42378-D02; Wimmelbach, close to Untere Mark, pond edge, 49.71, 10.994, 290 m, Malaise trap, 12.vii.2019, leg. J. Müller, ZSM-HYM-42381-C10; München, Obermenzing, Premises of Zoologische Staatssammlung, 48.1648, 11.4849, 519 m, Malaise trap, 31.vii.2017, leg. Axel Hausman, BIOUG42788-F01; **Serbia**: Southwestern Serbia, 2.3 km SE of Nova Varoš, along Creek, 43.443, 19.853, 1014 m, 14.vi.2009, leg. J. Skevington, CNCH1019; **Spain**: Can Liro; Barcelona, Catalonia, ex. *Vanessaatalanta*, 12.v.2006, leg. C. Stefanescu, WAM 0444; Catalonia, El Puig, ex. *Vanessacardui* on *Echium* sp., 15.vi.2016, leg. C. Stefanescu, MRS_JFT0714; **United Kingdom**: England, Beetham, Cumbria, ex. *Anthophilafabriciana*, 15.viii.2013, leg. M. R. Shaw, MRS_JFT0342.

##### Geographical distribution.

PAL.

PAL- Austria*, Belgium, France, Germany*, Greece, Serbia*, Spain*, United Kingdom.

##### Molecular data.

BIN: BOLD:ABY6385.

##### Host information.

Choerutidae: type reared from *Anthophilafabriciana* (Linnaeus, 1767); also *Prochoreutismyllerana* (Fabricius, 1794); Nymphalidae: *Aglaisurticae* (Linnaeus, 1758), *Vanessaatalanta* (Linnaeus, 1758); Vanessacardui* (Linnaeus, 1758); Pyralidae: *Pataniaruralis* (Scopoli, 1763).

##### Notes.

**Figure 34.**
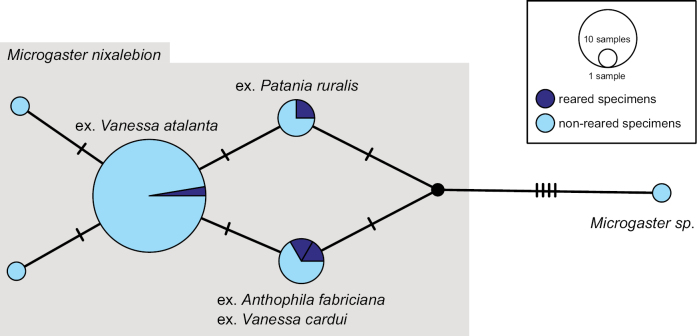
TCS haplotype network of BIN BOLD:ABY6385, sequence length for analysis: 392 bp to accommodate MRS_JFT0342=MARKB109-21 ex. *Anthophilafabriciana* from the United Kingdom. The haplotypes morphologically identified as *Microgasternixalebion* as part of this project are marked by a grey background. Each hatch mark in the network represents a single mutational change; small black dots at nodes indicate missing haplotypes. The diameter of the circles is proportional to the number of haplotypes sampled (see legend). The aligned sequences and traits can be reviewed in Suppl. materials 10, 11.

**Figure 35.**
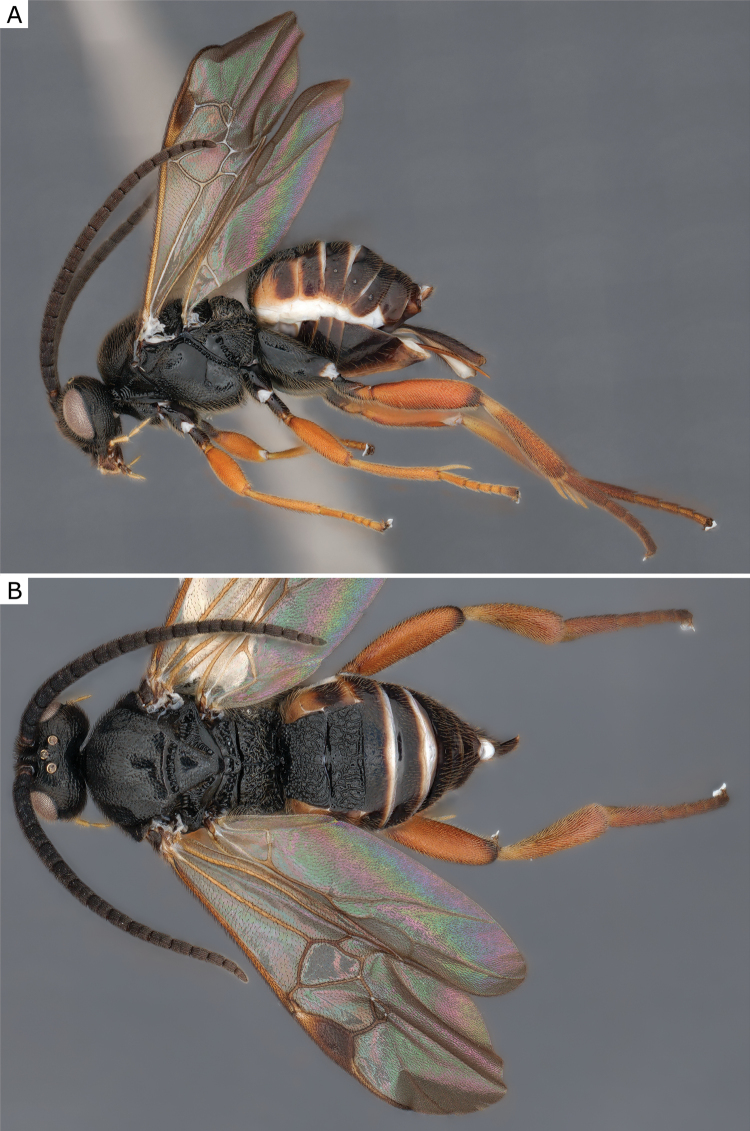
*Microgasternixalebion* Shaw, 2004, female (ZSM-HYM-42380-A06) **A** lateral and **B** dorsal views. Length of the specimen: 4.25 mm.

German specimens were identified by comparing with the information in the original description (Shaw 2004) as well as specimens deposited in the CNC. Our sequences from German specimens are a 100% match for barcode sequences of several reared specimens: two specimens identified as *M.nixalebion*, one reared from *Pataniaruralis* (MRS_JFT0934), one from *Vanessaatalanta* (WAM 0444=MRS_JFT 0029). There are two more reared specimens in this barcoding cluster, both males and initially determined only to genus, one from *Vanessacardui* (Linnaeus, 1758) (MRS_JFT0714) and one from *Anthophilafabriciana*, the host of the holotype (MRS_JFT0342). A single specimen in this barcoding cluster differs by several mutations from the other sequences; until more specimens for morphological study are available we keep it as *Microgaster* sp. (ZSM-HYM-42325-G10) (see Fig. 34). This species is illustrated in Fig. 35.

#### ﻿ Microgaster raschkiellae

Taxon classificationAnimaliaHymenopteraBraconidae

Shaw, 2012

D6E312AE-CF73-5283-8BCE-B29211AA76ED

##### Material examined.

**Germany**: Bavaria: Rhön Hausen, Eisgraben, basalt block heap at forest edge, 50.503, 10.09, 735 m, Malaise trap, 23.vii.2018, leg. D. Doczkal, ZSM-HYM-33166-C08; Rhön Hausen, Kleines Moor, 50.487, 10.039, 890 m, Malaise trap, 25.vii.2018, leg. D. Doczkal, ZSM-HYM-33165-G04; **United Kingdom**: Scotland: Armadale, Skye, ex. *Mompharaschkiella*, 4.vii.2012, leg. M. R. Shaw, CNCHYM45380.

##### Geographical distribution.

NEA, PAL.

NEA: Canada (MB); PAL: Germany*, United Kingdom.

##### Molecular data.

BOLD:AAC9130.

##### Host information.

Momphidae: type reared from *Mompharaschkiella* (Zeller, 1839).

##### Notes.

**Figure 36.**
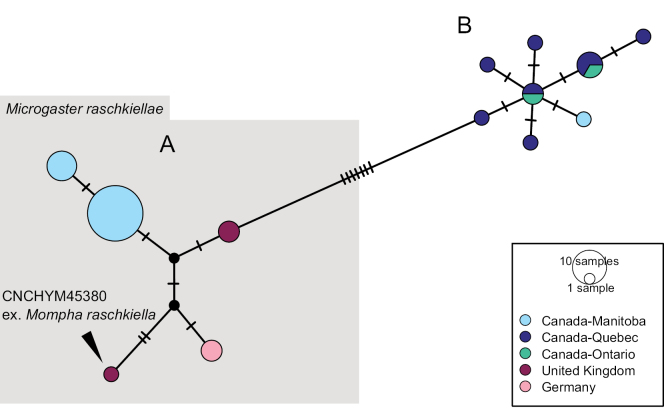
TCS haplotype network of BIN BOLD:AAC9130, the haplotypes morphologically identified as *Microgasterraschkiellae* as part of this project are in cluster A and marked by a grey background. Each hatch mark in the network represents a single mutational change; small black dots at nodes indicate missing haplotypes. The diameter of the circles is proportional to the number of haplotypes sampled (see legend). The aligned sequences and traits can be reviewed in Suppl. materials 12, 13.

**Figure 37.**
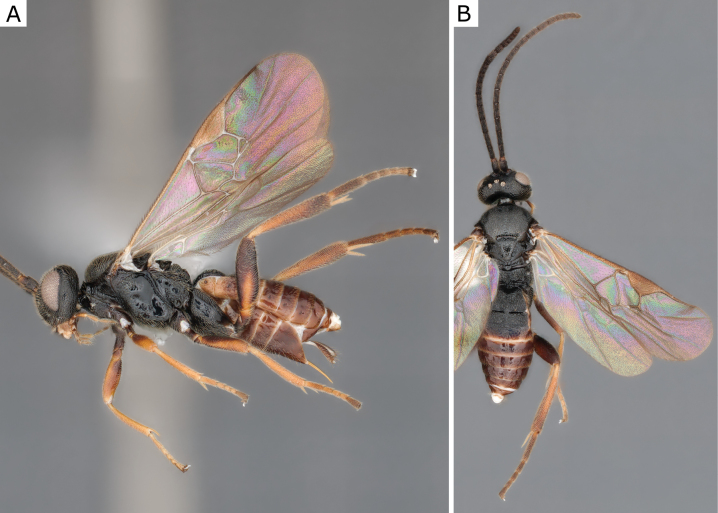
*Microgasterraschkiellae* Shaw, 2012, female (ZSM-HYM-33165-G04) **A** lateral and **B** dorsal views. Length of the specimen: 3.0 mm.

German specimens were identified by comparing to the original description (Shaw 2012a). BIN BOLD:AAC9130 probably includes more than one species. This BIN includes two haplotype clusters (Fig. 36) separated by seven mutations. Cluster A includes specimens from the United Kingdom identified by the original author of the species and reared from the same host as the holotype (CNCHYM45380=MRS_JFT0192). Our specimens collected from Germany cluster with these authoritatively identified specimens and match the species morphologically with some minor differences. We consider them *Microgasterraschkiellae* and report this species for Germany for the first time. Cluster B likely represents a different species from the Nearctic, but exploring this further is beyond the scope of this project. This species is illustrated in Fig. 37.

#### ﻿ Microplitis coactus

Taxon classificationAnimaliaHymenopteraBraconidae

(Lundbeck, 1896)

0C26B8F6-BB31-5666-92C9-935952CEBACD

##### Material examined.

**Canada**: Newfoundland and Labrador: Saglek, Torngat Mountains NP, Base Camp south of park, 58.451, -62.798, 5 m, 01.viii.2014, leg. D. Whitaker, BIOUG18647-F03; Nunavut: Ellesmere Island, Hazen Camp, 81.816667, -71.300000, [date unknown, leg. unknown], CNC497575; **Germany**: Bavaria: Atzmannsberg, Hessenreuther and Atzmannsberger Forst, 49.825, 11.963, 550 m, Malaise trap, 11.vii.2019, leg. J. Müller, ZSM-HYM-42384-B08; St. Oswald, National Park Bayerischer Wald, 48.9509, 13.422, 842 m, Malaise trap, 20.vi.2012, leg. G. Sellmayer, BIOUG05949-B01.

##### Geographical distribution.

NEA, PAL.

NEA- Canada (NL*, NU), Greenland; PAL- Germany*, Iceland.

##### Molecular data.

BIN: BOLD:ACA4555.

##### Host information.

Host of type unknown; also Noctuidae.

##### Notes.

**Figure 38.**
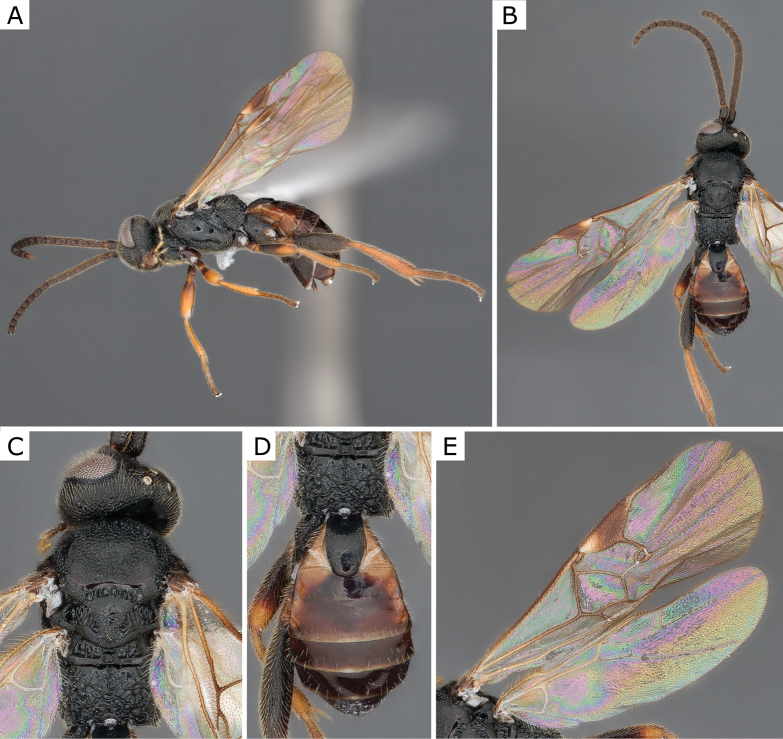
*Microplitiscoactus* (Lundbeck, 1896), female (ZSM-HYM-42384-B08) **A** lateral view **B** dorsal view **C** mesosoma **D** metasoma **E** wing. Length of the specimen: 2.6 mm.

**Figure 39.**
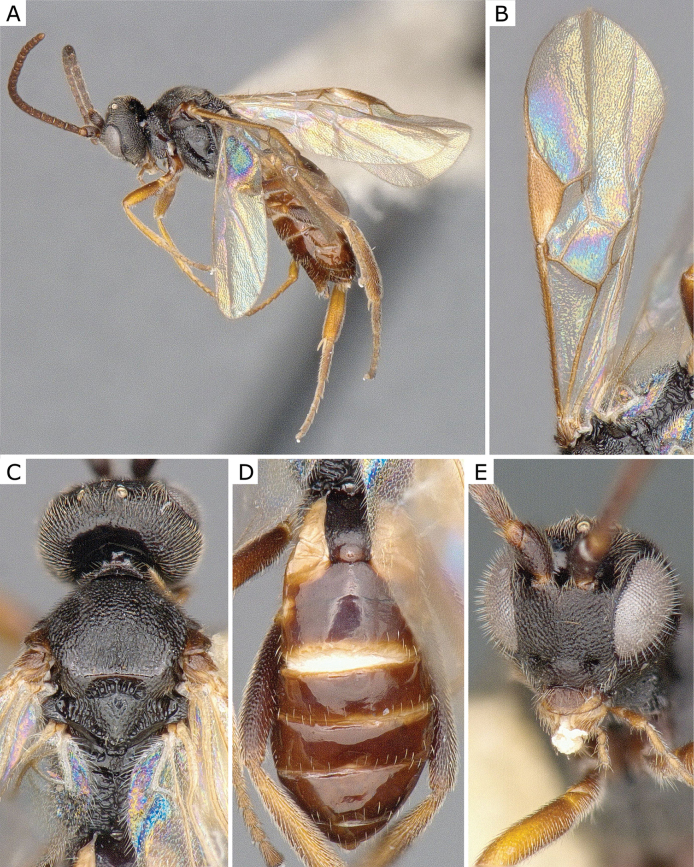
*Microplitiscoactus* (Lundbeck, 1896), female (CNC497575) **A** lateral view **B** forewing **C** mesosoma **D** metasoma **E** head frontal view.

German specimens were identified by comparison with many specimens at the CNC (see Figs 38, 39) and by checking the keys and information in Papp (1984b) and van Achterberg (2006) and the original description (Lundbeck 1896). The associated host information is taken from the original description of the species (Lundbeck 1896, 244) which stated that (loose translation from Danish follows): “there were nine specimens from earlier dates without a specific locality, all females; according to the inscription, they hatched from a *Noctua* species [this would refer just to a noctuid = Noctuidae at that time]. [...] The wasp cocoons seem to form a hollow ball and were found under rocks in several places in both northern and southern Greenland”. This is the first record of the species outside the Nearctic and Iceland. The sequences from Germany match well (0.31% p-distance) with the sequences from Greenland available in BOLD, and the corresponding BIN is fairly cohesive (average of 0.58% of bp difference within BIN and 0.96% max. p-distance within the BIN) and comparatively very well differentiated from any other BIN currently in BOLD (nearest BIN is at 3.57% p-distance), therefore confirming also from a molecular perspective the presence of this species in Europe. This species is illustrated in Figs 38, 39.

#### ﻿ Microplitis kewleyi

Taxon classificationAnimaliaHymenopteraBraconidae

Muesebeck, 1922

CCCADB0E-77C7-59A6-84B2-7076C905C232

##### Material examined.

**Germany**: Bavaria: Ammergebirge Halblech, Im Laich, gravel bar, 47.606, 10.841, 904 m, Malaise trap, 16.ix.2016, leg. D. Doczkal, J. Voith, ZSM-HYM-33167-F01; Forchheim, Untere Mark bei Willersdorf, 49.739, 10.969, 261 m, Malaise trap, 12.vii.2019, leg. J. Müller, ZSM-HYM-42377-F06; Lkr. Kelheim Abensberg-Sandharlanden, NSG Sandharlandener Heide, 48.845, 11.801, 376 m, Malaise trap, 3.viii.2017, leg. D. Doczkal, J. Voith, ZSM-HYM-33157-E03; Marquartstein, close to Rathaus, 47.759, 12.462, 543 m, Malaise trap, 19.vii.2019, leg. J. Müller, ZSM-HYM-42380-C10; Plattling, Isarmündung, renat. gravel bar, 48.781, 12.906, 317 m, Malaise trap, 25.viii.2021, leg. GBOL3, R. Albrecht, ZSM-HYM-42391-B09; Siegenburg, Bombodrom, 48.76, 11.807, 410 m, Malaise trap, 23.viii.2017, leg. D. Doczkal, J. Voith, ZSM-HYM-42324-C05; Sielstetten, östlich Grafendorfer Forst, 48.578, 11.863, 520 m, Malaise trap, 16.vii.2019, leg. J. Müller, ZSM-HYM-42383-A06; Willersdorf, Untere Mark, 49.733, 10.985, 292 m, Malaise trap, 12.vii.2019, leg. J. Müller, ZSM-HYM-42379-A12.

##### Geographical distribution.

NEA, PAL.

NEA- Canada (AB, MB, NB, NL, NS, ON, PE, QC), United States (CA, DC, IA, MD, MI, NJ, NY, WI); PAL*- Germany*.

##### Molecular data.

BIN: BOLD:AAB8493.

##### Host information.

Noctuidae: type reared from *Euxoa* sp.; also *Agrotisipsilon* (Hufnagel, 1766), *Euxoaochrogaster* (Guenée, 1852), ?*Pseudohermonassabicarnea* (Guenée, 1852).

##### Notes.

**Figure 40.**
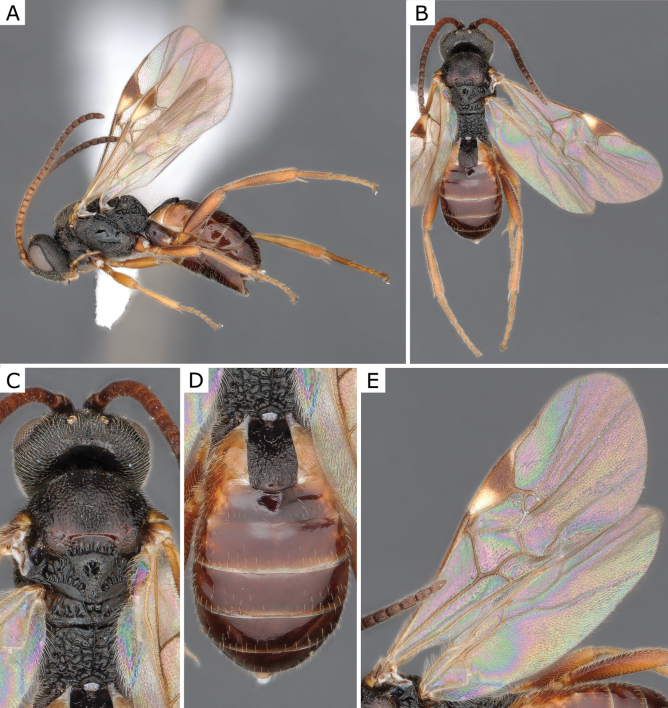
*Microplitiskewleyi* Muesebeck, 1922, female (ZSM-HYM-33157-E03). **A** lateral view, **B** dorsal view, **C** mesosoma, **D** metasoma, **E** wing. Length of the specimen: 2.35 mm.

German specimens were identified by comparison with many specimens in the CNC and by checking the keys and information in Muesebeck (1922). This is the first record of the species outside the Nearctic. A few described species in the Palearctic share some characteristics with *M.kewleyi* (and other Nearctic species), particularly the short antenna, large pale spot anteriorly on pterostigma, and relatively small body size (e.g., see couplet 21 in Nixon (1970) and Papp (1984b)). However, *M.kewleyi* can be distinguished from *M.spectabilis* based on shape of T1 and darker colour of anterior flagellomeres; from *M.tristis* because wings are not infumated, shorter inner spur of metatibia, and thinner femora; from *M.pallidipennis* based on shape and sculpture of T2; from *M.steinbergi* because of thinner metafemur and metatibia; and from *M.heterocerus* because of a much larger pale spot on pterostigma, different leg colouration and thinner femora. Eight sequences from Germany match 100% with the many sequences from Canada and the USA available in BOLD, and the corresponding BIN is very cohesive (average of 0.03% of bp difference within BIN members) and very apart from any other BIN currently in BOLD (nearest BIN is 5.77% different), therefore confirming also from a molecular perspective the presence of this species in Europe (Germany). There are also two sequences from Bulgaria, two from Pakistan, and one from Tanzania, but those specimens are from different institutions which we were not able to examine, and no photographs were available for them in BOLD either. Therefore, those countries are not recorded for the species here, even if the DNA barcode evidence indicates a reasonable chance that *M.kewleyi* could also be present there. Two of the hosts associated with this species were recorded by Muesebeck, who had described the wasp species and are therefore considered to be accurate. A third host, *Euxoaochrogaster*, is within the same genus as the host of the type and it is also considered to be reliable. Only one host record from the literature is here considered to be questionable, as it comes from a compilation of information without any supporting evidence. This species is illustrated in Fig. 40.

#### ﻿ Microplitis naenia

Taxon classificationAnimaliaHymenopteraBraconidae

Nixon, 1970

28E3827C-6793-5913-B4C5-3160B3733AB8

##### Material examined.

**Czech Republic**: South Moravia: Obora Soutok, Lanžhot, 48.69, 16.945, 165 m, ex. *Orthosiacruda*, 14.v.2013, leg. P. Drozd, BC-ZSM-HYM-23873-E06; **Germany**: Bavaria: Grettstadt, 49.963, 10.372, 248 m, canopy fogging, 28.v.2021, leg. B. Leroy, ZSM-HYM-42393-D05; Iphofen, 49.646, 10.315, 355 m, canopy fogging, 2.v.2019, leg. B. Leroy, ZSM-HYM-33158-A10; Prichsenstadt, 49.855, 10.304, 258 m, canopy fogging, 1.v.2019, leg. B. Leroy, ZSM-HYM-33158-A09; Schonungen, 50.077, 10.429, 359 m, canopy fogging, 23.v.2019, leg. B. Leroy, ZSM-HYM-33158-E05; ZSM-HYM-33158-E06; Wiesentheid, 49.803, 10.277, 216 m, canopy fogging, 1.v.2019, leg. B. Leroy, ZSM-HYM-33158-B02; ZSM-HYM-33158-B03; Thüringen: Neubrunn, 50.511, 10.457, 448 m, canopy fogging, 29.v.2021, leg. D. Rabl, ZSM-HYM-42447-D08.

##### Geographical distribution.

PAL.

PAL- Czech Republic*, Germany*, Hungary, Russia (C, NW), Slovakia, Turkey, United Kingdom.

##### Molecular data.

BINs: BOLD:ABV9098, BOLD:AEK2564.

##### Host information.

Host of type unknown; also Noctuidae: *Cosmiatrapezina* (Linnaeus, 1758), *Conistravaccinii* (Linnaeus, 1761), *Eupsiliatransversa* (Hufnagel, 1766), *Orthosiacruda* (Denis & Schiffermüller, 1775), *Orthosiacerasi* (Fabricius, 1775), *Rileyianafovea* (Treitschke, 1825).

##### Notes.

**Figure 41.**
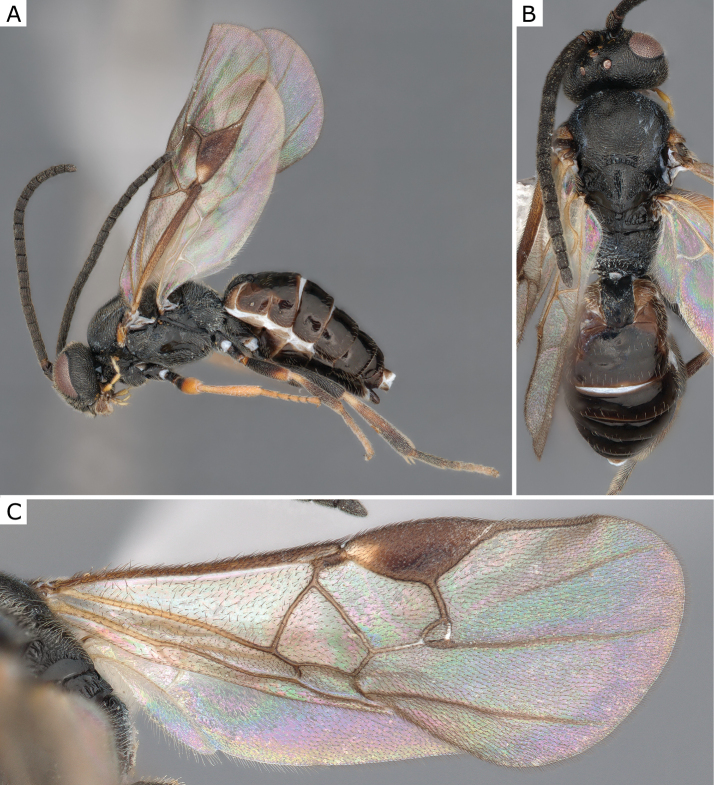
*Microplitisnaenia* Nixon, 1970, female (ZSM-HYM-42447-D08) **A** lateral view **B** meso- and metasoma **C** wing. Length of the specimen: 3.5 mm.

The morphology of our material (see Fig. 41) matches the species described in Nixon (1970), as well as the keys in Papp (1984b), Tobias (1986), and Kotenko (2007). There are two BINs which we associate with this species. Our specimens show some intraspecific morphological variability, which matches what Nixon mentioned in the original description of the species. Specimens of both barcoding clusters parasitise the same host, *Orthosiacruda*, from which two of Nixon’s paratypes were reared. One specimen from BOLD:ABV9098 (BC-ZSM-HYM-23873-E06) was reared from this species, and we have metabarcoding data that strongly indicates that members of BOLD:AEK2564 parasitise the same host species. Metabarcoding of 22 individual caterpillars of *O.cruda* resulted in more than 100 reads per caterpillar of sequences that match this BIN. With this information, we consider our specimens of both barcoding clusters to represent members of *M.naenia*. BIN BOLD:ABV9098 and BOLD:AEK2564 are separated by 1.71% p-distance and the species has an intraspecific variability of 2.63% (p-distance). The hosts associated with this wasp species were reported either by Nixon (1970), as part of the original description, or by Capek (1972) and Capek et al. (1982); we consider both to be reliable because some of the material reported by Nixon (1970) came from Capek. This species is illustrated in Fig. 41.

#### ﻿ Pholetesor bedelliae

Taxon classificationAnimaliaHymenopteraBraconidae

(Viereck, 1911)

42CFD911-4BB6-537D-8F20-E5A2BC1159E2

##### Material examined.

**Canada**: New Brunswick: Fredericton, 45.963487, -66.6442, 9.vii.1970, leg. C. M. Yoshimoto, CNCHYM 03145 [paratype]; **Germany**: Bavaria: Bayreuth, Gemein, Trebgast, 49.989, 11.603, 348 m, Malaise trap, 11.vii.2019, leg. J. Müller, ZSM-HYM-42385-C09; Volkach, Stammheim am Main, 49.92, 10.192, 215 m, Malaise trap, 16.vii.2019, leg. J. Müller, ZSM-HYM-42380-E08.

##### Geographical distribution.

AUS, NEA, NEO, PAL.

AUS: Hawaiian Islands; NEA: Canada (AB, BC, MB, NB, NS, ON, QC, SK), USA (AK, AZ, AR, CA, CT, DC, FL, IL, IA, KA, LA, MO, NJ, NY, OR, VA); NEO: Bermuda, Peru; PAL: Finland, Germany*.

##### Molecular data.

BIN: BOLD:AAA9172.

##### Host information.

Types reared from *Bedellia* sp. At least 21 host species within seven families of Lepidoptera have been recorded as hosts of this wasp species (Yu et al. 2016), but many may be incorrect and thus are not cited here.

##### Notes.

**Figure 42.**
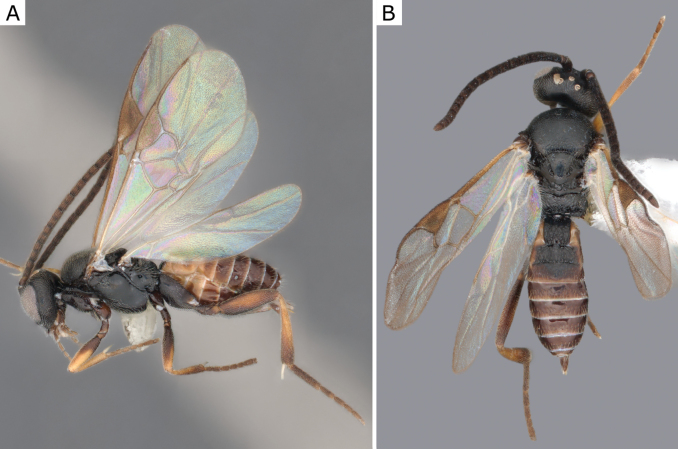
*Pholetesorbedelliae* (Viereck, 1911), female (ZSM-HYM-42380-E08) **A** lateral and **B** dorsal view. Length of the specimen: 2.15 mm.

We compared our specimens to a paratype and many other Nearctic specimens stored at the CNC (such as CNCHYM 03145), as well as the comprehensive description in Whitfield (2006). Morphologically, this species is somewhat similar to *Pholetesormaritimus* (Wilkinson, 1941), which is also recorded from Europe (Fernandez-Triana et al. 2020). However, the characters used by Nixon (1973) do not work in all cases to separate these species, as some specimens of *Pholetesorbedelliae* have coarse/strong sculpture (of T1, T2, and anteromesoscutum) that approaches or is very similar to the sculpture described for *P.maritimus*. DNA barcodes unequivocally show that the two species are far apart and that the German specimens clearly cluster with many specimens identified by us of *P.bedelliae* from Canada and USA. All these sequences are separated by ~ 5% p-distance from the sequences of *P.maritimus* currently available in BOLD (MRS_JFT0464, MRS_JFT0471). Many hosts associated with *P.bedelliae* in the historical literature are probably incorrect, due to problems identifying both the wasp and caterpillar species (e.g., see Whitfield 2006); solving this problem is beyond the scope of this paper. This species is illustrated in Fig. 42.

#### ﻿ Protapanteles endemus

Taxon classificationAnimaliaHymenopteraBraconidae

(Nixon, 1965)

CD0BD7F1-64B4-5C1E-B5E7-67AF2FF3684F

##### Material examined.

**France**: Jura, Ounans, ex. *Thyatirabatis*, 25.vii.2013, leg. M. R. Shaw, MRS_JFT0355; **Germany**: Baden-Württemberg: Malsch, Hansjakobstr. 7, Urban Garden, 48.884, 8.32, 120 m, Malaise trap, 30.viii.2020, leg. D. Doczkal, ZSM-HYM-33153-G08; Malsch, Luderbusch, 48.913, 8.332, 117 m, Malaise trap, 19.vii.2020, leg. D. Doczkal, K. Grabow, ZSM-HYM-42388-D09; **Poland**: Biebrza National Park, cocoons on *Ribesnigrum* with *Abraxasgrossulariata*, leg. M. R. Shaw, MRS_JFT0444.

##### Geographical distribution.

PAL.

PAL: France, Germany*, Hungary, Kazakhstan, Poland*, Russia (ZAB, SPE), Switzerland, Ukraine, United Kingdom.

##### Molecular data.

BIN: BOLD:AEI1558.

##### Host information.

Geometridae: Type reared from *Abraxasgrossulariata* (Linnaeus, 1758); also Drepanidae*: Thyatirabatis* (Linnaeus, 1758).

##### Notes.

**Figure 43.**
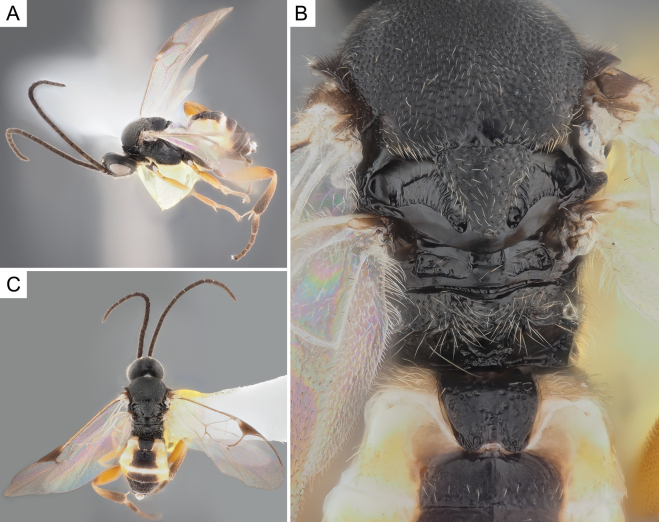
*Protapantelesendemus* (Nixon, 1965), female (ZSM-HYM-33153-G08) **A** lateral view **B** mesosoma, T1, and T2 **C** dorsal view. Length of the specimen: 2.5 mm.

The German specimens were identified based on information from the original description (Nixon 1965) as well as subsequent papers (Nixon 1976; Papp 1984a). Our German sequences 99.7–100% match two identical sequences of specimens with some host relations: one was collected as a cocoon from *Ribes* sp. with *Abraxasgrossulariata* (MRS_JFT0444), the Geometrid species the holotype was reared from (Nixon 1965). Another specimen was reared from *Thyatirabatis* (MRS_JFT0355), a rather novel host and member of Drepanidae. We propose here that an additional host record from the literature is excluded: Tobias (1971: 246) had recorded *Autographagamma* (Linnaeus, 1758) Noctuidae as a host of *Protapantelesendemus* in the St. Petersburg (as Leningrad) region; however, a subsequent paper from the same author (Tobias 1986: 661) ignored that host record and even the record of the wasp species from that region (it only recorded *P.endemus* from a much more southern region of the former Soviet Union, now Ukraine). It is likely that either the wasp, the host or both were misidentified in the 1971 paper and therefore this record must be removed (unless additional data in the future confirms that association). This species is illustrated in Fig. 43.

#### ﻿ Rasivalva desueta

Taxon classificationAnimaliaHymenopteraBraconidae

Papp, 1989

DEDF7065-D256-5505-9C5D-FF06C6FD3964

##### Material examined.

**Germany**: Bavaria: Bayernwald National Park, Schöberg, ex. ?*Eilemadepressa*, 16.vi.2015, leg. M. R. Shaw, MRS-JFT 0582; Berchtesgaden National Park, Königssee, Rinnkendlsteig, 47.555, 12.965, 750 m, Malaise trap, 21.viii.2017, leg. D. Doczkal, J. Voith, ZSM-HYM-33162-A05; **Sweden**: Öland: Gamla Skogsby (Kalkstad); Mörbylånga kommun, 56.616700, 16.507617, Malaise trap, 17.vii-7.viii.2003, leg. SMTP, CNC602554.

##### Geographical distribution.

PAL.

PAL- Germany*, Sweden*, Switzerland.

##### Molecular data.

BIN: BOLD:ADE2589.

##### Host information.

Host of type unknown. Other host associations in need of verification.

##### Notes.

**Figure 44.**
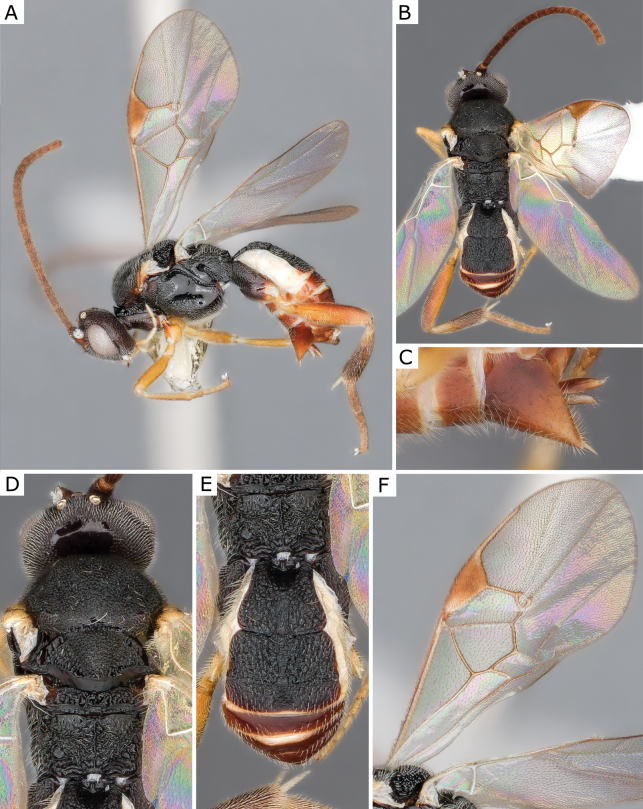
*Rasivalvadesueta* Papp, 1989, female (ZSM-HYM-33162-A05) **A** lateral view **B** dorsal view **C** hypopygium lateral view **D** mesosoma **E** metasoma **F** forewing. Length of the specimen: 3.3 mm.

**Figure 45.**
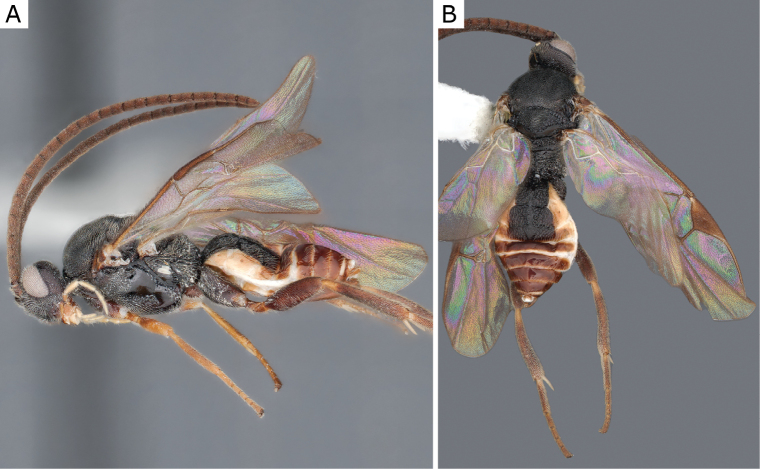
*Rasivalvadesueta* Papp, 1989, male (CNC602554) **A** lateral and **B** dorsal views.

The non-reared German specimen was identified as *Rasivalvadesueta* by comparing it with the detailed original description (Papp 1989) and Oltra-Moscardó and Jiménez-Peydró (2005). One male specimen (MRS-JFT 0582) in this cluster, also from Germany, was possibly reared from *Eilemadepressa* (Esper, 1787) which would represent the first host record for this wasp species. However, the wasp cocoon could not be associated unequivocally with host remains and hence the host association needs to be confirmed. We also report Sweden as a new country record based on a male specimen we examined (CNC602554), which is morphologically similar to the German female (except for darker metafemur and less broad T1 and T2, in agreement with the original description of a male paratype) as well as matching DNA barcode sequences. This species is illustrated in Figs 44, 45.

### ﻿Additional species

#### ﻿ Cotesia eulipis

Taxon classificationAnimaliaHymenopteraBraconidae

(Nixon, 1974)

B9D869AE-0157-519D-9FA3-DF20BB78B6E7

##### Material examined.

**Canada**: British Columbia: Winfield, 50.061, -119.431, [450m,] ex. *Operophterabruceata*, 17.v.2001, leg. K. Deglow, CNCHYM 00330; CNCHYM 00331; Prince Edward Island: Brackley Beach, Prince Edward Island National Park, 46.431111, -63.216111, ex. *Rheumapterahastata*, 10.viii.1940, leg. G.S. Walley, CNC1447813; Yukon: Top of the World Highway km 82, 64.09, -140.951, 19.vii.2006, leg. H. Goulet, C. Boudreault, HYM00001784; **Finland**: Lapland: Utsjoki, Kevo, ex. *Operophterabrumata*, 28.vi.2010, leg. K. Ruohomaki, MRS 0049; MRS_JFT0049; Utsjoki, Vetsikko, ex. *Operophterabrumata*, 28.vi.2010, leg. K. Ruohomaki, MRS 0050; **Germany**: Bavaria; St. Oswald, National Park Bayerischer Wald, 48.951, 13.422, 842 m, Malaise trap, 25.vii.2012, leg. G. Sellmayer, BIOUG07768-G08; **Norway**: Rogaland, Hana, ex. *Operophterabrumata*, 28.vi.2010, leg. K. Ruohomaki, MRS 0048; **United Kingdom**: Scotland: Argyll, Scotnish Farm, ex. *Rheumapterahastata*, 25.iv.1990, leg. K. P. Bland, CNCHYM49288.

##### Geographical distribution.

NEA, PAL.

NEA*- Canada* (BC, PE, YT); PAL- Bulgaria, Finland, Germany, Greece, Hungary, Norway*, Sweden, United Kingdom.

##### Molecular data.

BIN: BOLD:ACZ1254.

##### Host information.

Geometridae: type reared from *Rheumapterahastata* (Linnaeus, 1758); also Operophterabruceata* (Hulst, 1886), Operophterabrumata* (Linnaeus, 1758).

##### Notes.

**Figure 46.**
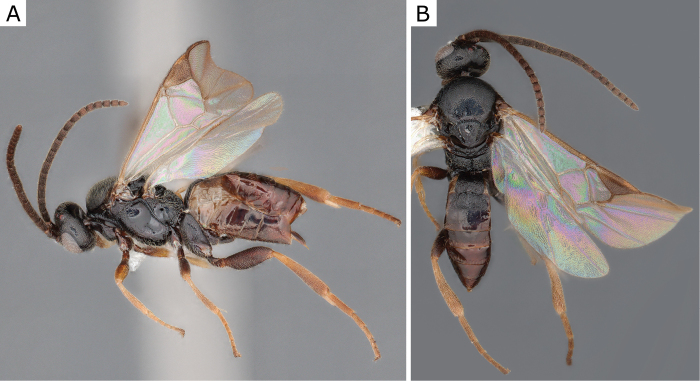
*Cotesiaeulipis* (Nixon, 1974), female (BIOUG07768-G08) **A** lateral and **B** dorsal views. Length of the specimen: 2.75 mm.

**Figure 47.**
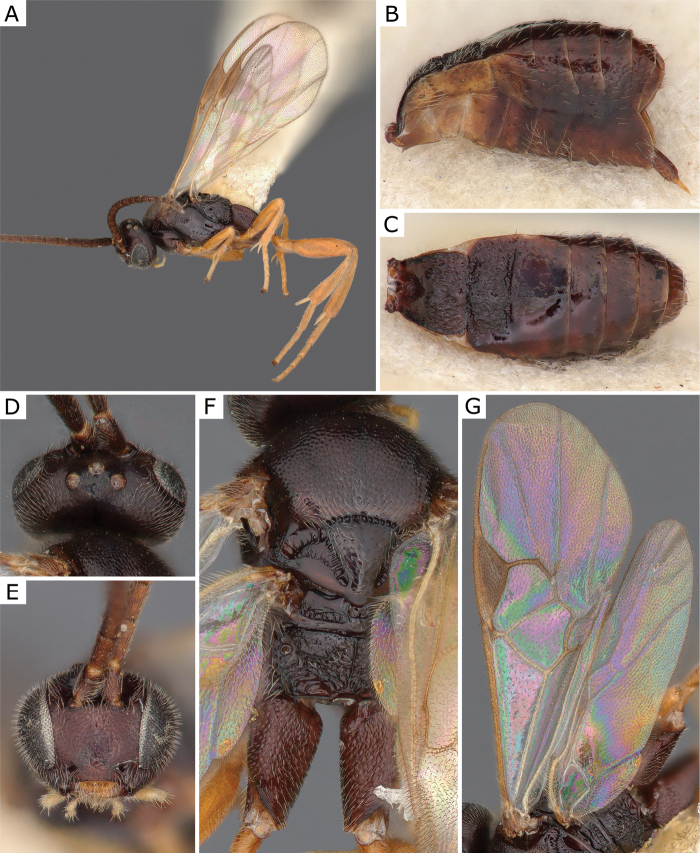
*Cotesiaeulipis* (Nixon, 1974), female (CNC1447813) **A** lateral view **B** metasoma lateral view **C** metasoma dorsal view **D** head dorsal view **E** head frontal view **F** mesosoma dorsal view **G** wing. Length of the specimen: head + mesosoma 1.5 mm, metasoma 1.0 mm.

Barcoding cluster BOLD:ACZ1254 includes sequences of specimens from Finland, Germany, Norway, United Kingdom, and Canada. Specimens in this cluster from both sides of the Atlantic were reared from *Rheumapterahastata* (the host of the holotype): from the United Kingdom (CNCHYM49288=MRS_JFT 0154) and from Canada (CNC1447813). Morphological examination resulted in some differences in leg colouration between Canadian and European specimens available to us (see Figs 46, 47). However, T1–T3 shape and structure are similar and the host information and DNA barcoding also point towards all of our specimens representing one species. Based on this integrative species concept from studying specimens from German as well as some Canadian and European material, and considering the hosts of this species, we here consider BIN BOLD:ACZ1254 to represent *C.eulipis* and report this species for the Nearctic for the first time. Other specimens in this BIN were reared from *Operophterabrumata* (MRS 0048=MRS_JFT0048, MRS 0049=MRS_JFT0049, MRS 0050=MRS_JFT0050) in Finland and Norway and from *Operophterabruceata* (CNCHYM 00330, CNCHYM 00331) in Canada. There is another specimen from Austria (CNC990841=MRS_JFT0743) that morphologically matches *C.eulipis*, yet clusters in a different BIN (BOLD:AEV7769) which has a p-distance of 1.9% to all other specimens that we identified as *C.eulipis* and is reared from *Hydriacervinalis* (Scopoli, 1763), a host so far not reported for this species. Resolving this goes beyond the scope of this paper, for the purpose of this manuscript we prefer to retain this specimen as “Cotesiacf.eulipis”. This species is illustrated in Figs 46, 47.

#### ﻿ Cotesia tetrica

Taxon classificationAnimaliaHymenopteraBraconidae

(Reinhard, 1880)

6B197A1C-A8A9-5D90-B28F-4BB823B88247

##### Material examined.

**Austria**: Lower Austria, Raglitz, ex. *Aphantopushyperantus*, 04.v.2011, leg. J. Connell, MRS 0054; **Germany**: Bavaria: Allgäu, Oberstdorf, Oytal, Schochen, alpine meadow, 47.392, 10.37, 1930 m, 6.viii.2014, leg. D. Doczkal, S. Schmidt, J. Voith, BC-ZSM-HYM-24109-E04; Balderschwang, Leiterberg, 47.486, 10.09, 1290 m, Malaise trap, 25.viii.2017, leg. D. Doczkal, J. Voith, ZSM-HYM-42325-F05; Garmisch-Partenkirchen, Zugspitze, Platt, 47.405, 11.009, 1980 m, Malaise trap, 2.viii.2018, leg. D. Doczkal, J. Voith, ZSM-HYM-42390-G12; Lkr. Weilheim, Pähl, Hartschimmelhof Niedermoor west of Goasl, 47.942, 11.182, 713 m, Malaise trap, 18.viii.2021, leg. GBOL3, R. Albrecht, ZSM-HYM-42398-E12; Rhön Hausen, Kleines Moor, 50.487, 10.039, 890 m, Malaise trap, 25.vii.2018, leg. D. Doczkal, ZSM-HYM-33165-G09.

##### Geographical distribution.

PAL.

PAL- Austria*, Germany, Montenegro, Serbia, United Kingdom.

##### Molecular data.

BIN: BOLD:AAV9103.

##### Host information.

Host of type unknown; also Nymphalidae: Satyrinae*: *Lasiommatamegera* (Linnaeus, 1767), *Maniolajurtina* (Linnaeus, 1758); Aphantopushyperantus* (Linnaeus, 1758).

##### Notes.

**Figure 48.**
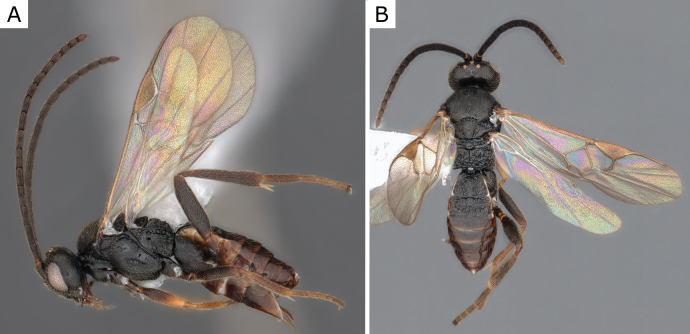
*Cotesiatetrica* (Reinhard, 1880), female (ZSM-HYM-42325-F05) **A** lateral and **B** dorsal views. Length of the specimen: 2.55 mm.

This species was previously recorded from Germany (Belokobylskij et al. 2003). Germany was also mentioned as the type locality in the world checklist (Fernandez-Triana et al. 2020); however, the country was not added to the distribution range of that species by error, so we confirm it here. Other specimens morphologically identified as *Cotesiatetrica* clustering in this BIN were reared from *Aphantopushyperantus* in Austria (MRS 0054, CNC990821, CNC990822), and there are barcoded specimens in NMS from several identified European species of *Erebia* (M. Rindos and MRS pers. obs) in the same BIN. Host records in the historical literature from other Lepidoptera families are incorrect, and Wilkinson (1945: 84) had already advised against reporting those records. The distribution in Serbia is based on Žikić et al. (2021). This species is illustrated in Fig. 48.

#### ﻿ Diolcogaster claritibia

Taxon classificationAnimaliaHymenopteraBraconidae

(Papp, 1959)

DF0C09B3-77B3-53F7-8195-38B4A72DBC03

##### Material examined.

**Canada**: Ontario: Ottawa, Central Experimental Farm, DBM Field Cage Trials, 45.389959, -75.711949, 23.vi.2010, leg. P. Mason, S. Girardoz, CNCHYM 01692; CNCHYM 01693; CNCHYM 01694; **CYPRUS**: Amathus, 21-iv-1966, leg. Mavromoustakis, CNCHYM 00892; **FRANCE**: Languedoc-Roussillon: Baillargues, Herault, 43.662, 4.014, 3-vi-1995, leg. P. Mason, CNCH1127; Bel Air; Herault, 43.639, 3.75, 5-vi-1995, leg. P. Mason, CNCH1126; **Germany**: Baden-Württemberg: Malsch, Hansjakobstr. 7, Urban Garden, 48.884, 8.32, 120 m, Malaise trap, 19.vii.2020, leg. D. Doczkal, ZSM-HYM-33154-G11; 5.vii.2020, leg. D. Doczkal, ZSM-HYM-33152-H07; Bavaria: Bayreuth, Laineck, 49.959, 11.618, 358 m, Malaise trap, 11.vii.2019, leg. J. Müller, ZSM-HYM-42375-C09; ZSM-HYM-42375-C10; ZSM-HYM-42375-D01; ZSM-HYM-42375-D03; Bobingen, cemetery, 48.272, 10.84, 524 m, Malaise trap, 16.vii.2019, leg. J. Müller, ZSM-HYM-42385-C11; Forkendorf, close to Thiergarten, 49.903, 11.555, 423 m, Malaise trap, 10.vii.2019, leg. J. Müller, ZSM-HYM-42385-D06; Hassfurt, Mechenried, 50.096, 10.483, 254 m, Malaise trap, 12.vii.2019, leg. J. Müller, ZSM-HYM-42383-C09; ZSM-HYM-42383-C10; Iphofen, Mönchsondheim, 49.668, 10.28, 263 m, Malaise trap, 16.vii.2019, leg. J. Müller, ZSM-HYM-42384-E02; Volkach, Kolitzheim, 49.922, 10.234, 229 m, Malaise trap, 16.vii.2019, leg. J. Müller, ZSM-HYM-42378-D08; Wimmelbach, close to Untere Mark, pond edge, 49.71, 10.994, 290 m, Malaise trap, 12.vii.2019, leg. J. Müller, ZSM-HYM-42381-D01; Wunsiedel, Waldrand SW von Wintersreuth, 50.035, 12.039, 538 m, Malaise trap, 16.vii.2019, leg. J. Müller, ZSM-HYM-42380-B06; **Jordan**: East Jordan: Wadi Schaib, 9-ii-1968, leg. J.S. Klapperich, CNCHYM 00893; **Netherlands**: Gelderland: Wageningen, ex *Plutellaxylostella*, 2012, leg. J. Harvey, CNCHYM45357.

##### Geographical distribution.

NEA, PAL.

NEA- Canada (AB, MB, ON); PAL- Afghanistan, Armenia, Austria, Azerbaijan, Belarus, Cyprus, Finland, France, Georgia, Germany, Greece, Hungary, Italy, Iran, Jordan, Kazakhstan, Lithuania, Macedonia, Moldova, Netherlands, Russia (ZAB, KDA), Spain, Syria, Tunisia, Turkey, Turkmenistan, Ukraine, former Yugoslavia.

##### Molecular data.

BIN: BOLD:AAH1034, BOLD:AEV8838.

##### Host information.

Host of type unknown; also Plutellidae: *Plutellaarmoraciae* Busck, 1912, *Plutellaxylostella* (Linnaeus, 1758).

##### Notes.

**Figure 49.**
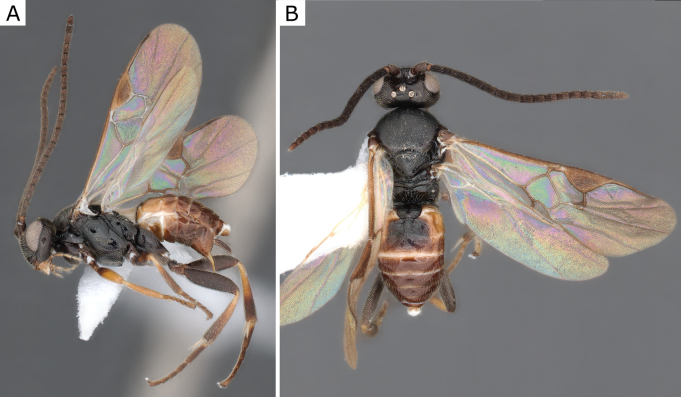
*Diolcogasterclaritibia* (Papp, 1959), female (ZSM-HYM-33152-H07) **A** lateral and **B** dorsal views. Length of the specimen: 2.25 mm.

German specimens were identified based on the detailed species concept in Fernandez-Triana et al. (2014b). One of the hosts currently associated with this species, *Plutellaxylostella*, has been widely reported in the literature (e.g., Papp 1981b; Fernandez-Triana et al. 2014a) and the wasp is commonly reared from it. Our sequences match specimens reared from *P.xylostella* (CNCHYM 01692, CNCHYM 01693, CNCHYM 01694) in Canada and a sequence from another specimen from the Netherlands (CNCHYM45357=MRS_JFT0220) also reared from the same host; the maximum p-distance for those specimens and the German material is 0.82%. Specimens of this species cluster in two BINs which are separated by 2.14% but also show a high within-BIN variability of 2.28% and 2.64%. A second host species, *Plutellaarmoraciae*, has been recently recorded for this wasp in Canada (Abram et al. 2022). The reared wasps are not yet sequenced. Whether *D.claritibia* represents a single species or a complex of morphologically cryptic species is beyond the scope of the present paper and will need further study including more (reared) specimens from both the Palearctic and the Nearctic. The hosts were first recorded by Papp (1981b) and Abram et al. (2022). The distribution of this species in Germany was mentioned by Papp (1981b), but to our knowledge not cited in literature since, so we confirm it here. Additionally, we add the distribution for Syria, which was also mentioned by Papp (1981b), but not cited since. Finally, we correct the distribution from the world checklist and add the records from Cyprus, France, Italy, Jordan, and the Netherlands reported by Fernandez-Triana et al. (2014b). This species is illustrated in Fig. 49.

#### ﻿ Microgaster procera

Taxon classificationAnimaliaHymenopteraBraconidae

Ruthe, 1860

D8CF51D2-1623-5256-9ACA-76353D83956B

##### Material examined.

**Canada**: Prince Edward Island: Near Georgetown; Georgetown, 46.417, -62.667, 8 m, 31.vii.2005, leg. M. Sharkey, WMIC 0244; WMIC 0245; **Germany**: Bavaria: Berchtesgaden National Park, Königssee, Rinnkendlsteig, 47.553, 12.964, 775 m, Malaise trap, 30.vii.2017, leg. D. Doczkal, J. Voith, ZSM-HYM-33160-F06; Berchtesgaden National Park, Wald west of St.Bartholomä, 47.547, 12.965, 620 m, Malaise trap, 28.vi.2017, leg. D. Doczkal, J. Voith, ZSM-HYM-33156-E05; ZSM-HYM-33156-E06; ZSM-HYM-33156-E07; Schmelzenholzham, Waldrand, 48.488, 13.124, 468 m, Malaise trap, 12.vii.2019, leg. J. Müller, ZSM-HYM-42325-B01; Siegenburg, Bombodrom, 48.755, 11.791, 411 m, Malaise trap, 13.vii.2017, leg. D. Doczkal, J. Voith, ZSM-HYM-42324-D03.

##### Geographical distribution.

NEA, PAL.

NEA*- Canada* (PE); PAL- Austria, Finland, Germany, Hungary, Ireland, Mongolia, Netherlands, Poland, Romania, Russia (SPE), Spain, Ukraine.

##### Molecular data.

BIN: BOLD:AAA9548.

##### Host information.

Host of type unknown. Shaw (2012a) gives tentative hosts of some British specimens (see below).

##### Notes.

**Figure 50.**
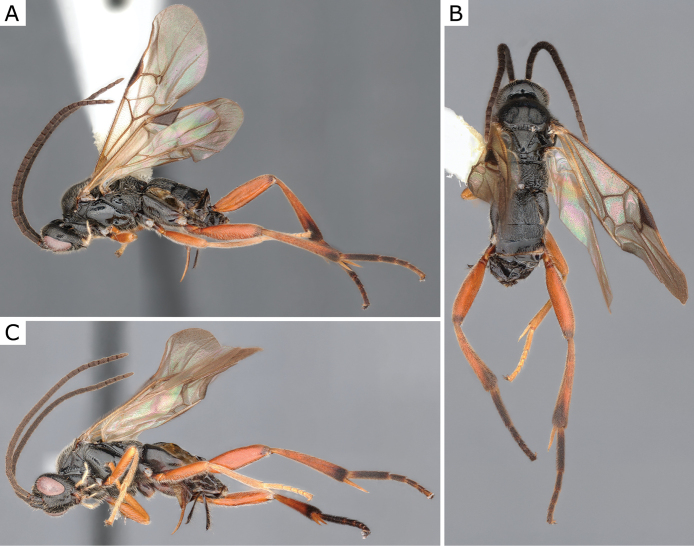
*Microgasterprocera* Ruthe, 1860, female (WMIC0245) **A** lateral view **B** dorsal view **C** lateroventral view. Length of the specimen: 4.15 mm.

**Figure 51.**
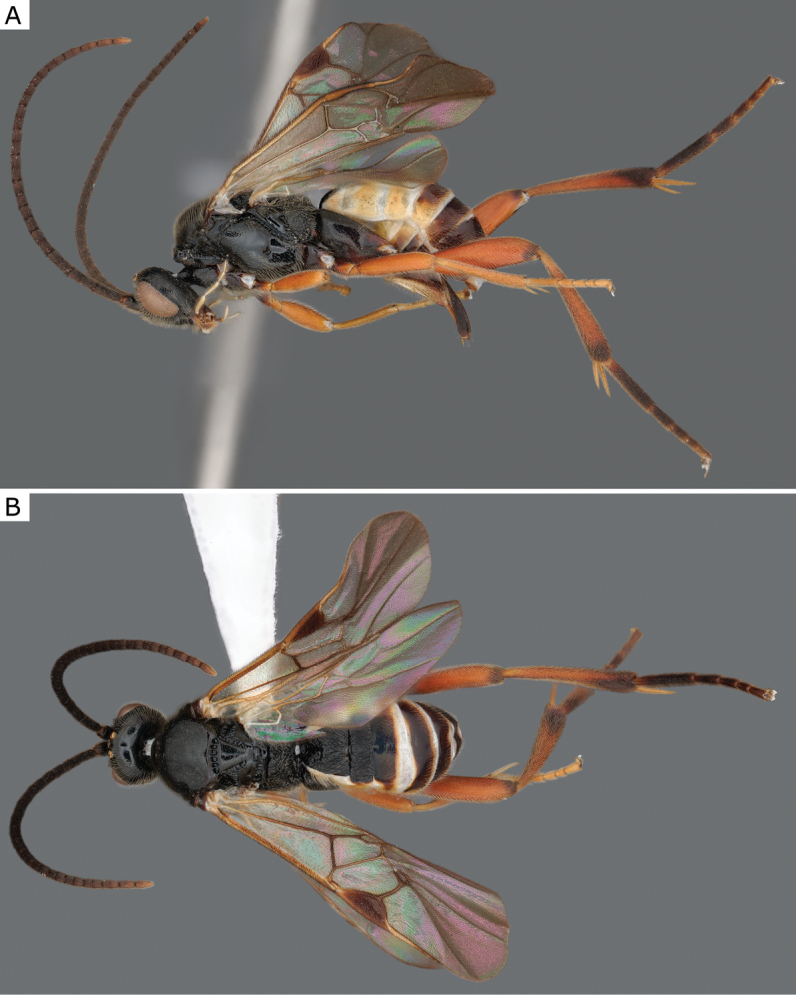
*Microgasterprocera* Ruthe, 1860, female (ZSM-HYM-33160-F06) **A** lateral and **B** dorsal views. Length of the specimen: 5.25 mm.

This is not a new record for Germany, but morphological examination of German material and assignment of the species name to a barcoding cluster allow us to record the species in the Nearctic for the first time. The Canadian specimens were compared to the European material and all sequences in this barcoding cluster have a maximum pairwise-distance of just 0.15%. The host information for this species must be considered as mostly unreliable; based on a recent discussion (Shaw 2012a: 194) it seems as though the crambid *Ananiahortulata* could be the most trustable record, but even that needs verification. This species is illustrated in Figs 50, 51.

## ﻿Discussion

### ﻿DNA Barcoding

Processing 5364 specimens in this reversive DNA barcoding approach combined with integrative taxonomy enabled us to gather many new distributions as well as much biological data in a relatively short time frame. We were able to report 30 species for Germany for the first time, four species as occurring in the Holarctic for the first time and, by also taking into account material already determined in NMS and CNC collection, publish 26 additional country records, disclose ten new host-parasitoid associations (five of which are new host families), link ten species with DNA barcodes for the first time, and, based mainly on recently collected German material, illustrate many species for which photographs were not previously available. We were also able to link males and females in a few cases (*Choerasciscaucasicus*, *Rasivalvadesueta*), although we mostly selected female specimens for examination. We increased the known German fauna of Microgastrinae by more than 10% from 252 species (Papp 1981b; Belokobylskij et al. 2003; Fernandez-Triana et al. 2020; Shaw 2020, 2022) to 282 species and provide a first step towards a comprehensive barcode library for German Microgastrinae.

We used the framework of the BOLD workbench and database as it already includes more than 65,000 sequences of Microgastrinae worldwide (www.boldsystems.org, accessed on 20 Aug 2023) and has been used as a main molecular analysis tool in Microgastrinae taxonomy in the past (Smith et al. 2013; Fernandez-Triana et al. 2014c; Fagan-Jeffries et al. 2018). It is important to note that all this barcoding data available for Microgastrinae wasps is still very much underrepresenting the molecular diversity of the group and hence molecular species hypotheses might change in the future. We critically tested all our molecular species hypotheses by using morphology and biology in our integrative species concepts.

We investigated BINs that had high within-BIN maximum p-distances (> 2.2%) (Ratnasingham and Hebert 2013) and BINs with low p-distances to their Nearest Neighbour (< 2.2%) (Raupach et al. 2020). Using this as a rough method for screening our molecular clusters, we found several cases of BIN discrepancies. In one case a species was represented by more than one BIN (= over-splitting: *Microplitisnaenia*BOLD:ABV9098 and BOLD:AEK2564, potentially *Diolcogasterclaritibia*BOLD:AAH1034 and BOLD:AEV8838). This does not represent a major problem for molecular species identifications as long as the multiple BINs would be associated with a single taxon. In fact, the two BINs that represent *M.naenia* are Nearest Neighbours (NN) and separated only by a minimum NN p-distance of 1.70%. This case of over-splitting probably represents intraspecific variation and, once more haplotypes are sampled, those barcoding clusters might eventually merge. There are other BINs that are close (p-dist < 2.2%) to their Nearest Neighbour: *Cotesiaeulipis*BOLD:ACZ1254, *Dolichogenideacerialis*BOLD:AAZ9570, *Microgastercaris*BOLD:ACN6851. Those clusters could potentially be problematic; however, in those cases, the within-BIN maximum p-distance is much lower than the minimum NN p-distance. Meanwhile, cases of BIN-sharing represent a problem for DNA-based species identification: In those cases, a BIN includes several species (= BIN-sharing: e.g., *Cotesiarisilis* in BIN BOLD:AAA6099).

BOLD:AAA7143 is an even more extreme case of BIN-sharing, it includes four species of *Cotesia* treated in this paper: *C.coryphe*, *C.mendicae*, *C.selenevora*, and *C.subordinaria*. When starting our analyses in 2022, each of these species was part of a separate BIN but merged into this “megaBIN” in early 2023. In Table 1, the previous BINs are included in brackets below the current BIN. This cluster includes a total of 1772 members (last updated 07 Aug 2023) and several possibly misidentified families, subfamilies, and genera. We performed ASAP clustering for a subset of sequences: this revealed 163 clusters in the first partition (ASAP score 3.99) and 141 clusters in the second partition (ASAP score 6.50). Our subset includes 1471 sequences, 100 interim species identifications (indicating different morphotypes), and 26 species. This “megaBIN” is clearly not useful in clustering these species of *Cotesia*. This problem is likely temporary as it only appeared by spring 2023 and might be resolved, e.g., by removing some sequences that might be causing these problems.

**Table 1.** P-distances and member counts of the BINs treated in this paper, retrieved on 8 Aug 2023. Single asterisks (*) mark each species name that was matched to a BIN for the first time, Double asterisks (**) mark each species that was sequenced for the first time. A BIN in parentheses shows that the BIN represents more than one species and cannot be used for molecular identification of the species. A BIN in brackets shows the previous BIN before February 2023. If the minimum NN p-distance is < 2.2% or within BIN maximum p-distance > 2.2%, then the within-BIN max. p-distance and minimum NN-p-distance are in bold font.

Species names	BIN(s)	Members (BIN)	BIN-compliant members	within- BIN average p-dist	within-BIN max. p-dist	min. NN p-dist
*Apantelesgalleriae*	BOLD:AAG1400	25	13	0.38%	1.17%	2.36%
*Apanteleskubensis*	BOLD:AAH1340	7	5	0.14%	0.32%	5.61%
Choerasciscaucasicus*	BOLD:ACU3996	5	1	0.22%	0.48%	6.78%
Choerasgnarus*	BOLD:AAU6216	41	12	0.08%	0.52%	2.23%
*Cotesiacoryphe*	(BOLD:AAA7143) [BOLD:ABY6805]	1772 [25]	859 [5]	3.85% [0.16%]	7.61% [**0.71**%]	2.21% [**1.50**%]
*Cotesiaeulipis*	BOLD:ACZ1254	16	4	0.29%	**0.59**%	**1.18**%
*Cotesiaeunomiae*	BOLD:AAV9098	6	0	0.18%	0.34%	7.68%
*Cotesiainducta*	BOLD:AAV9096	4	1	0.22%	0.34%	2.48%
*Cotesiamendicae*	(BOLD:AAA7143) [BOLD:ABY8119]	1772 [N/A]	859 [N/A]	3.85% [N/A]	7.61% [N/A]	2.21% [N/A]
*Cotesiarisilis*	(BOLD:AAA6099)	182	105	1.25%	**2.87**%	**1.12**%
*Cotesiaselenevora*	(BOLD:AAA7143) [BOLD:AAA9381]	1772 [23]	859 [6]	3.85% [0.62%]	7.61% [**2.84**%]	2.21% [**1.40**%]
*Cotesiasubordinaria*	(BOLD:AAA7143) [BOLD:ACO3220]	1772 [5]	859 [0]	3.85% [0.07%]	7.61% [**0.19**%]	2.21% [**1.44**%]
*Cotesiatetrica*	BOLD:AAV9103	25	17	0.29%	0.66%	2.84%
Deuterixysplugarui**	BOLD:AEJ7518	9	0	0.54%	0.96%	5.83%
*Diolcogasterclaritibia*	BOLD:AAH1034	95	73	0.34%	**2.28**%	**2.14**%
	BOLD:AEV8838	16	8	0.66%	**2.64**%	**2.14**%
*Dolichogenideacerialis*	BOLD:AAZ9570	8	5	0.28%	**0.49**%	**1.28**%
Dolichogenideacheles*	BOLD:ACQ9527	10	5	0.57%	1.42%	3.33%
Dolichogenideacoleophorae**	BOLD:AEO8197	2	0	0.00%	0.00%	2.72%
*Glyptapantelesindiensis*	BOLD:ABY2372	16	8	0.31%	0.81%	3.52%
*Glyptapantelespopovi*	BOLD:AEJ4298	7	0	0.23%	0.64%	3.25%
Illidopscloelia**	BOLD:AEO8223	2	0	0.00%	0.00%	4.81%
Illidopssplendidus**	BOLD:AEJ7519	1	0	N/A	N/A	6.58%
*Microgasterarctostaphylica*	BOLD:AAH1039	12	7	0.93%	**2.25**%	**2.68**%
*Microgastercaris*	BOLD:ACN6851	66	4	0.17%	**0.72**%	**1.60**%
*Microgasternervosae*	BOLD:ACR4142	11	3	0.35%	0.82%	2.72%
*Microgasternixalebion*	BOLD:ABY6385	62	12	0.20%	**1.48**%	**0.97**%
Microgasterprocera*	BOLD:AAA9548	8	3	0.04%	0.16%	5.33%
*Microgasterraschkiellae*	BOLD:AAC9130	36	25	0.91%	**2.26**%	**1.93**%
*Microplitiscoactus*	BOLD:ACA4555	6	4	0.58%	0.96%	3.57%
*Microplitiskewleyi*	BOLD:AAB8493	127	53	0.03%	0.80%	5.77%
*Microplitisnaenia*	BOLD:ABV9098	18	17	0.35%	**1.28**%	**1.70**%
	BOLD:AEK2564	9	2	0.36%	**0.64**%	**1.70**%
*Pholetesorbedelliae*	BOLD:AAA9172	74	55	0.46%	1.62%	3.29%
Protapantelesendemus*	BOLD:AEI1558	2	1	0.17%	0.17%	7.64%
Rasivalvadesueta*	BOLD:ADE2589	4	2	0.08%	0.17%	3.04%

Overall, a more general observation from discussing integrative species concepts in our dataset is that the distances between barcoding clusters can vary substantially between genera. We were able to observe several cases of lumping (or BIN-sharing) in *Cotesia* with p-distances of ~ 1.0–1.5% between morphologically and biologically well-established species. We had to exclude some species from this paper because the situation turned out to be more complex than previously expected (e.g., *Cotesiaeuchloevora* Shaw, 2020 and *Cotesiapilicornis* (Thomson, 1895); see discussion of these two species in Shaw and Fernandez-Triana 2020). Other genera such as *Illidops* or *Deuterixys* were not affected by this and showed p-distances of ~ 4–10% between species in our larger dataset (pers. obs.). This has been documented and discussed for other taxa (e.g., Schütte et al. 2023). For now, we recommend that barcoding data for Microgastrinae wasps should be critically analysed, also from a genus-specific point of view.

So far, barcoding has worked well for the majority of Microgastrinae species and has been an essential tool in advancing the study of Microgastrinae diversity (Smith et al. 2013; Fernandez-Triana et al. 2014c; Fagan-Jeffries et al. 2018). Cases of BIN-discrepancies, depending on the case, may or may not cause problems for identifying species by DNA barcoding. Still, barcoding works very well for a large proportion of species (90–99%) in various insect taxa (Hausmann et al. 2011a, b; Hendrich et al. 2015; Schmidt et al. 2015; Schmid-Egger et al. 2019; Raupach et al. 2020), and less well in others, such as Orthoptera (Hawlitschek et al. 2017). At this point, it is difficult to evaluate the level of agreement between molecular species hypotheses (in our case BINs) and morphological and biological species concepts in Microgastrinae. The ideal case would be a 1:1 match between molecular clusters (BINs) and morphological (or biological) species. Although we conclude that barcoding data needs to be interpreted carefully in Microgastrinae wasps and should be paired with other methods, it also made this study possible in the first place. DNA barcoding enabled us to tackle this dark taxon at such a scale in a short time by clustering large amounts of sequences linked to thousands of specimens. We could link our material to previously barcoded material in the databases that provided additional information about (1) species identification (often narrowing down possible identifications), (2) biological interactions (even if hosts were parasitised by solitary males – information that is otherwise potentially lost as males can usually not be identified by morphology alone), (3) geographical distribution (especially important as some species have wide distribution ranges and may not always be included in the keys traditionally used for Western Europe), (4) phenology, and (5) sexes not available in our material, etc. DNA barcoding gave us access to these different aspects of our integrative species concepts.

**Figure 52.**
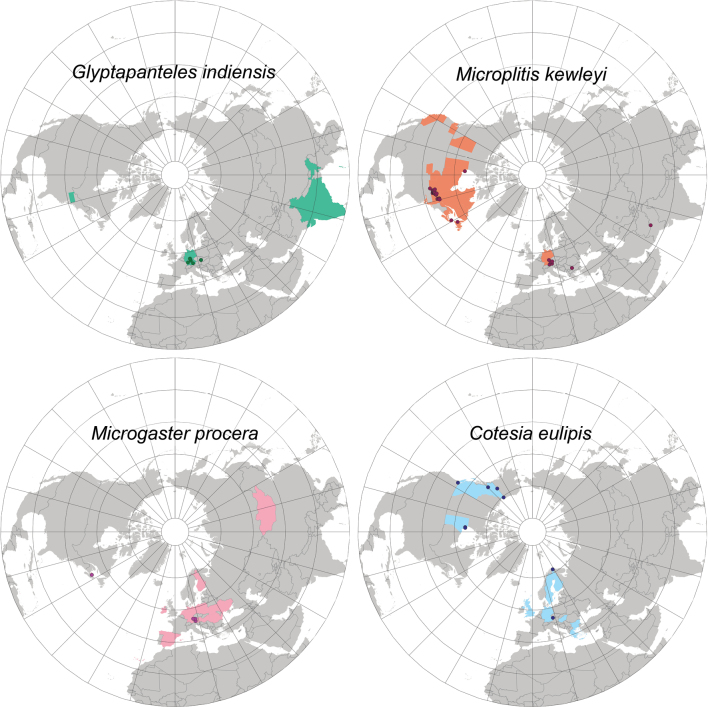
Neighbour-joining topology of the barcoding region of our dataset of the analysed species based on Kimura 2-parameter distances. Triangles show the relative number of individuals sampled (height) and sequence divergence (width). Pale blue colouration indicates species associated with BINs that have a maximum within-BIN distance > 2.2%. Dark blue colouration indicates species associated with BINs that have a higher within-BIN p-distance compared to NN p-distance. Orange colouration indicates species maximum intraspecific distances > 2.2% (cases of “BIN-sharing”). Numbers next to nodes represent non-parametric bootstrap values > 90% (1,000 replicates). Sequences shorter than 400 bp were excluded from this analysis. The aligned sequences and N-J topology can be reviewed in Suppl. materials 3 and 4.

Fig. 52 provides a Neighbour-Joining topology for our dataset, providing a first-glance impression of sample size and sequence divergence of the species treated here. In addition, our dataset is linked to the information about BIN distances of more than 65,000 sequences of Microgastrinae registered in the BOLD database, which is represented via colouration of the triangles: orange triangles indicate species with a minimum p-distance to the Nearest Neighbour < 2.2%, blue triangles indicate species that cluster in BINs with a maximum within-BIN distance > 2.2%. This N-J topology is not representative of any fauna as it only includes a few selected species per genus (here compared to the number of species reported for Germany): *Apanteles* (2/12), *Choeras* (2/9), *Cotesia* (9/62), *Deuterixys* (1/3), *Diolcogaster* (1/9), *Dolichogenidea* (3/45), *Glyptapanteles* (2/24), *Illidops* (2/4), *Microgaster* (6/32), *Microplitis* (3/39), *Pholetesor* (1/11), *Protapanteles* (1/12), and *Rasivalva* (1/3).

### ﻿Distribution in Europe and the Holarctic region

The known distribution in Europe of some species is significantly expanded: *Choerasciscaucasicus* was previously only known from Russia and Lithuania; in this case the new record from Germany expands this species’ range to the western part of Europe. *Microgasterarctostaphylica* and *Microgasternervosae*, two recently described species (Shaw 2012a, 2023) are recorded outside of the United Kingdom for the first time. *Microplitiscoactus* has been known to be distributed in the Nearctic, except for Iceland (PAL). Our record from Germany presents a major expansion of the known range of this species.

**Figure 53.**
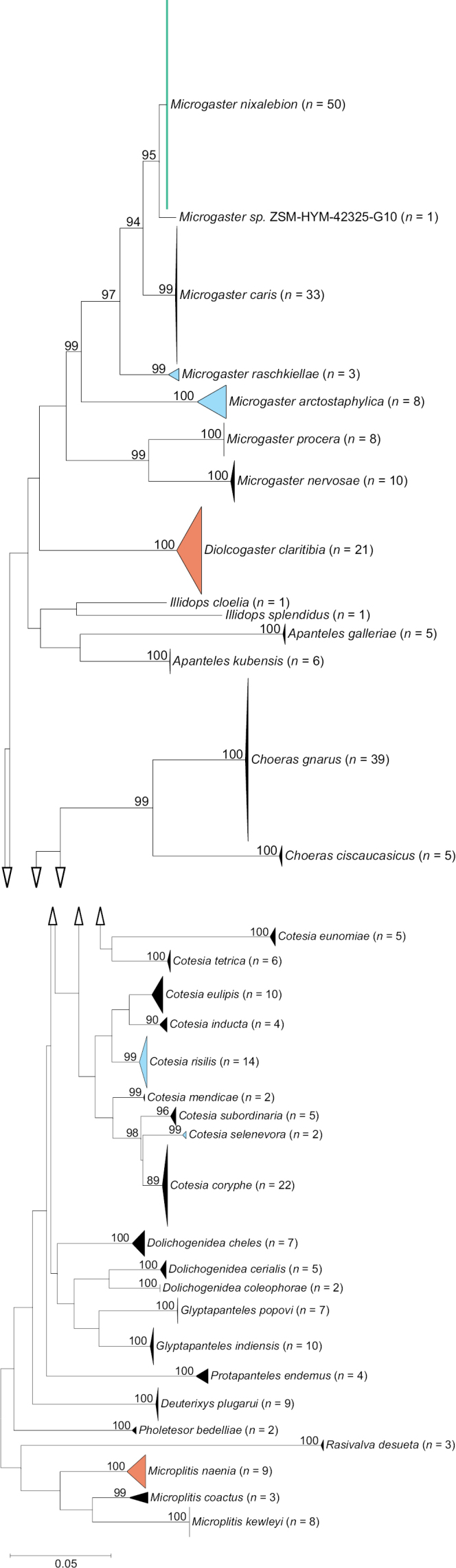
Distribution maps for the four newly recorded Holarctic species. Coloured countries/states show the known distribution as in Fernandez-Triana et al. (2020) with confirmed additional data from this study. Dots show sampling locations of the species from our dataset and mined public data from the matching BINs from BOLD. In these cases, the countries/states were not coloured because we were not able to confirm this by studying the specimens. The coordinates used for the distributions can be reviewed in Suppl. material 5.

A main result of this study is that four species were recorded for the first time for the Palaearctic (*Glyptapantelesindiensis*, *Microplitiskewleyi*) and Nearctic regions (*Cotesiaeulipis*, *Microgasterprocera*). According to the supplementary material of the world checklist published in 2020, only 5% (56) of the 1,178 Holarctic species of Microgastrinae are shared between the Nearctic and Palaearctic regions. Of these 56 species, 29 have an exclusively Holarctic distribution (Fernandez-Triana et al. 2020) whereas the remaining species have a wider distribution, partly due to parasitising pests or species otherwise spread by human activity (such as *Apantelesgalleriae* which parasitises wax moths commonly found in beehives). These relatively low percentages of shared species are misleading, as most studies available (identification keys and taxonomic revisions) usually cover only the Nearctic or the Palaearctic region, but rarely both (the exceptions are smaller genera which are revised at wider range (e.g., Papp 1986b; Whitfield and Oltra Moscardó 2004; Fernandez-Triana et al. 2014c; Fujie et al. 2021). With DNA barcoding and the use of international databases and collaborations, we now have efficient tools at our hands to make more of these transatlantic and worldwide connections. For some species discussed here (Fig. 53) we already know that their distribution exceeds even the Holarctic region: the molecular cluster that we consider to represent *Microplitiskewleyi* also has members in Bulgaria and Pakistan (which is part of the Oriental region following O’Hara et al. 2009). This is based on publicly available data and shows a much wider distribution range compared to what we formally report here (as we were not able to observe the specimens). *Glyptapantelesindiensis* is another more widely distributed species which was first described from the Oriental region. All four species in Fig. 53 show somewhat patchy distributions, which likely do not represent the actual range of these species, but rather point out sampling bias. Most sequences in the BOLD database are from either North America or Western Europe. For Microgastrinae specifically, the countries with the most entries are Costa Rica (25512), Canada (14256), Germany (5650), United States (2453), and Australia (1781). Other than these, Holarctic countries with most entries are Turkey (433), Norway (361), Japan (258), United Kingdom (257), and Sweden (243) (https://www.boldsystems.org/, accessed 18 Aug 2023). Accounting for previously known distribution ranges and historical data (represented by the coloured countries/states) somewhat mitigates this, but sampling is still a significant source of bias in the species distributions that might also influence our current species concepts. With more international collaborations and as DNA barcoding becoming more accessible in the future, sampling might hopefully become more equally distributed.

## Supplementary Material

XML Treatment for Apanteles galleriae

XML Treatment for Apanteles kubensis

XML Treatment for Choeras ciscaucasicus

XML Treatment for Choeras gnarus

XML Treatment for Cotesia coryphe

XML Treatment for Cotesia eunomiae

XML Treatment for Cotesia inducta

XML Treatment for Cotesia mendicae

XML Treatment for Cotesia risilis

XML Treatment for Cotesia selenevora

XML Treatment for Cotesia subordinaria

XML Treatment for Deuterixys plugarui

XML Treatment for Dolichogenidea cerialis

XML Treatment for Dolichogenidea cheles

XML Treatment for Dolichogenidea coleophorae

XML Treatment for Glyptapanteles indiensis

XML Treatment for Glyptapanteles popovi

XML Treatment for Illidops cloelia

XML Treatment for Illidops splendidus

XML Treatment for Microgaster arctostaphylica

XML Treatment for Microgaster caris

XML Treatment for Microgaster nervosae

XML Treatment for Microgaster nixalebion

XML Treatment for Microgaster raschkiellae

XML Treatment for Microplitis coactus

XML Treatment for Microplitis kewleyi

XML Treatment for Microplitis naenia

XML Treatment for Pholetesor bedelliae

XML Treatment for Protapanteles endemus

XML Treatment for Rasivalva desueta

XML Treatment for Cotesia eulipis

XML Treatment for Cotesia tetrica

XML Treatment for Diolcogaster claritibia

XML Treatment for Microgaster procera

## ﻿Additional information

### Author ORCIDs

Amelie Höcherl https://orcid.org/0009-0007-4211-7468

Mark R. Shaw https://orcid.org/0000-0002-6651-8801

Caroline Boudreault https://orcid.org/0000-0002-4511-2626

Dominik Rabl https://orcid.org/0000-0002-0613-7804

Michael J. Raupach https://orcid.org/0000-0001-8299-6697

Stefan Schmidt https://orcid.org/0000-0001-5751-8706

Viktor Baranov https://orcid.org/0000-0003-1893-3215

José Fernández-Triana https://orcid.org/0000-0003-0425-0309

### Data availability

All of the data that support the findings of this study are available in the main text or Supplementary Information.

## Supplementary materials

Supplementary material 1 Specimen Data Spreadsheet (BOLD) for DS-MCGNRECS Data type xlsx Explanation note This table includes all relevant data associated with specimens that are included in our BOLD dataset (341 specimens). https://binary.pensoft.net/file/965665This dataset is made available under the Open Database License (http://opendatacommons.org/licenses/odbl/1.0/). The Open Database License (ODbL) is a license agreement intended to allow users to freely share, modify, and use this Dataset while maintaining this same freedom for others, provided that the original source and author(s) are credited.Author: Amelie Höcherl

Supplementary material 2 COI-Sequences for DS-MCGNRECS Data type fas Explanation note Includes all COI sequences used in our interative species concepts. https://binary.pensoft.net/file/965666This dataset is made available under the Open Database License (http://opendatacommons.org/licenses/odbl/1.0/). The Open Database License (ODbL) is a license agreement intended to allow users to freely share, modify, and use this Dataset while maintaining this same freedom for others, provided that the original source and author(s) are credited.Author: Amelie Höcherl

Supplementary material 3 Alignment used for NJ-topology of DS-MCGNRECS Data type fasta Explanation note Includes the alignment and all sequences used for the NJ-topology in Fig. 52, sequences > 400 bp. https://binary.pensoft.net/file/965667This dataset is made available under the Open Database License (http://opendatacommons.org/licenses/odbl/1.0/). The Open Database License (ODbL) is a license agreement intended to allow users to freely share, modify, and use this Dataset while maintaining this same freedom for others, provided that the original source and author(s) are credited.Author: Amelie Höcherl

Supplementary material 4 NJ-topology of DS-MCGNRECS Data type nwk https://binary.pensoft.net/file/965668This dataset is made available under the Open Database License (http://opendatacommons.org/licenses/odbl/1.0/). The Open Database License (ODbL) is a license agreement intended to allow users to freely share, modify, and use this Dataset while maintaining this same freedom for others, provided that the original source and author(s) are credited.Author: Amelie Höcherl

Supplementary material 5 Coordinates for Holarctic distribution maps Data type csv Explanation note Includes the coordinates used for the distribution maps in Fig. 53. https://binary.pensoft.net/file/965669This dataset is made available under the Open Database License (http://opendatacommons.org/licenses/odbl/1.0/). The Open Database License (ODbL) is a license agreement intended to allow users to freely share, modify, and use this Dataset while maintaining this same freedom for others, provided that the original source and author(s) are credited.Author: Amelie Höcherl

Supplementary material 6 *Glyptapantelesindiensis* and related species sequences Data type fasta Explanation note All sequences used for NJ-topology in Fig. 24. https://binary.pensoft.net/file/965670This dataset is made available under the Open Database License (http://opendatacommons.org/licenses/odbl/1.0/). The Open Database License (ODbL) is a license agreement intended to allow users to freely share, modify, and use this Dataset while maintaining this same freedom for others, provided that the original source and author(s) are credited.Author: Amelie Höcherl

Supplementary material 7 *Glyptapantelesindiensis* and related species NJ-topology Data type nwk Explanation note NJ-topology in Fig. 24. https://binary.pensoft.net/file/965671This dataset is made available under the Open Database License (http://opendatacommons.org/licenses/odbl/1.0/). The Open Database License (ODbL) is a license agreement intended to allow users to freely share, modify, and use this Dataset while maintaining this same freedom for others, provided that the original source and author(s) are credited.Author: Amelie Höcherl

Supplementary material 8 BOLD:AAA6099 sequences Data type nexus Explanation note Sequences in BIN BOLD:AAA6099 used for haplotype network in Fig. 14 for *Cotesiarisilis*. https://binary.pensoft.net/file/965672This dataset is made available under the Open Database License (http://opendatacommons.org/licenses/odbl/1.0/). The Open Database License (ODbL) is a license agreement intended to allow users to freely share, modify, and use this Dataset while maintaining this same freedom for others, provided that the original source and author(s) are credited.Author: Amelie Höcherl

Supplementary material 9 BOLD:AAA6099 traits Data type txt Explanation note Amelie Höcherl Traits (countries) for BIN BOLD:AAA6099 used for haplotype network in Fig. 14 for *Cotesiarisilis*. https://binary.pensoft.net/file/965673This dataset is made available under the Open Database License (http://opendatacommons.org/licenses/odbl/1.0/). The Open Database License (ODbL) is a license agreement intended to allow users to freely share, modify, and use this Dataset while maintaining this same freedom for others, provided that the original source and author(s) are credited.Author: Amelie Höcherl

Supplementary material 10 BOLD:ABY6385 sequences Data type nexus Explanation note Sequences in BOLD:ABY6385 used for haplotype network in Fig. 34 for *Microgasternixalebion*. https://binary.pensoft.net/file/965674This dataset is made available under the Open Database License (http://opendatacommons.org/licenses/odbl/1.0/). The Open Database License (ODbL) is a license agreement intended to allow users to freely share, modify, and use this Dataset while maintaining this same freedom for others, provided that the original source and author(s) are credited.Author: Amelie Höcherl

Supplementary material 11 BOLD:ABY6385 traits Data type txt Explanation note Traits (hosts) for BIN BOLD:ABY6385 used for haplotype network in Fig. 34 for *Microgasternixalebion*. https://binary.pensoft.net/file/965675This dataset is made available under the Open Database License (http://opendatacommons.org/licenses/odbl/1.0/). The Open Database License (ODbL) is a license agreement intended to allow users to freely share, modify, and use this Dataset while maintaining this same freedom for others, provided that the original source and author(s) are credited.Author: Amelie Höcherl

Supplementary material 12 BOLD:AAC9130 sequences Data type nexus Explanation note Sequences in BIN BOLD:AAC9130 used for haplotype network in Fig. 36 for *Microgasterraschkiellae*. https://binary.pensoft.net/file/965676This dataset is made available under the Open Database License (http://opendatacommons.org/licenses/odbl/1.0/). The Open Database License (ODbL) is a license agreement intended to allow users to freely share, modify, and use this Dataset while maintaining this same freedom for others, provided that the original source and author(s) are credited.Author: Amelie Höcherl

Supplementary material 13 BOLD:AAC9130 traits Data type txt Explanation note Traits (hosts) for BIN BOLD:AAC9130 used for haplotype network in Fig. 36 for *Microgasterraschkiellae*. https://binary.pensoft.net/file/965677This dataset is made available under the Open Database License (http://opendatacommons.org/licenses/odbl/1.0/). The Open Database License (ODbL) is a license agreement intended to allow users to freely share, modify, and use this Dataset while maintaining this same freedom for others, provided that the original source and author(s) are credited.Author: Amelie Höcherl

Supplementary material 14 ASAP partition 1 of BOLD:AAA7143 Data type csv Explanation note Sequences downloaded 7^th^ of august, 2023, ASAP score 3.99. https://binary.pensoft.net/file/965678This dataset is made available under the Open Database License (http://opendatacommons.org/licenses/odbl/1.0/). The Open Database License (ODbL) is a license agreement intended to allow users to freely share, modify, and use this Dataset while maintaining this same freedom for others, provided that the original source and author(s) are credited.Author: Amelie Höcherl

Supplementary material 15 ASAP partition 2 of BOLD:AAA7143 Data type csv Explanation note Sequences downloaded 7^th^ of august, 2023, ASAP score 6.50. https://binary.pensoft.net/file/965679This dataset is made available under the Open Database License (http://opendatacommons.org/licenses/odbl/1.0/). The Open Database License (ODbL) is a license agreement intended to allow users to freely share, modify, and use this Dataset while maintaining this same freedom for others, provided that the original source and author(s) are credited.Author: Amelie Höcherl
